# Forward genetic screen using a gene-breaking trap approach identifies a novel role of *grin2bb*-associated RNA transcript (*grin2bbART*) in zebrafish heart function

**DOI:** 10.3389/fcell.2024.1339292

**Published:** 2024-03-08

**Authors:** Ramcharan Singh Angom, Adita Joshi, Ashok Patowary, Ambily Sivadas, Soundhar Ramasamy, Shamsudheen K. V., Kriti Kaushik, Ankit Sabharwal, Mukesh Kumar Lalwani, Subburaj K., Naresh Singh, Vinod Scaria, Sridhar Sivasubbu

**Affiliations:** ^1^ Genomics and Molecular Medicine, CSIR Institute of Genomics and Integrative Biology, Delhi, India; ^2^ Department of Biochemistry and Molecular Biology, Mayo Clinic College of Medicine and Science, Jacksonville, FL, United States; ^3^ GN Ramachandran Knowledge Center for Genome Informatics, Council of Scientific and Industrial Research, Institute of Genomics and Integrative Biology, Delhi, India; ^4^ Academy of Scientific and Innovative Research, Ghaziabad, India

**Keywords:** insertional mutagenesis, gene breaking trap, calcium homeostasis, grin2bb, grin2bbART, hypertrophy, arrhythmia, RNA sequencing

## Abstract

LncRNA-based control affects cardiac pathophysiologies like myocardial infarction, coronary artery disease, hypertrophy, and myotonic muscular dystrophy. This study used a gene-break transposon (GBT) to screen zebrafish (*Danio rerio*) for insertional mutagenesis. We identified three insertional mutants where the GBT captured a cardiac gene. One of the adult viable GBT mutants had bradycardia (heart arrhythmia) and enlarged cardiac chambers or hypertrophy; we named it “bigheart.” Bigheart mutant insertion maps to grin2bb or N-methyl D-aspartate receptor (NMDAR2B) gene intron 2 in reverse orientation. Rapid amplification of adjacent cDNA ends analysis suggested a new insertion site transcript in the intron 2 of grin2bb. Analysis of the RNA sequencing of wild-type zebrafish heart chambers revealed a possible new transcript at the insertion site. As this putative lncRNA transcript satisfies the canonical signatures, we called this transcript *grin2bb associated RNA transcript (grin2bbART)*. Using *in situ* hybridization, we confirmed localized *grin2bbART* expression in the heart, central nervous system, and muscles in the developing embryos and wild-type adult zebrafish atrium and bulbus arteriosus. The bigheart mutant had reduced Grin2bbART expression. We showed that bigheart gene trap insertion excision reversed cardiac-specific arrhythmia and atrial hypertrophy and restored *grin2bbART* expression. Morpholino-mediated antisense downregulation of *grin2bbART* in wild-type zebrafish embryos mimicked bigheart mutants; this suggests *grin2bbART* is linked to bigheart. Cardiovascular tissues use Grin2bb as a calcium-permeable ion channel. Calcium imaging experiments performed on bigheart mutants indicated calcium mishandling in the heart. The bigheart cardiac transcriptome showed differential expression of calcium homeostasis, cardiac remodeling, and contraction genes. Western blot analysis highlighted Camk2d1 and Hdac1 overexpression. We propose that altered calcium activity due to disruption of *grin2bbART*, a putative lncRNA in bigheart, altered the Camk2d-Hdac pathway, causing heart arrhythmia and hypertrophy in zebrafish.

## Introduction

Long non-coding RNAs (lncRNAs) affect transcriptional and post-transcriptional gene expression, cell differentiation, and tissue function (Hu et al., 2012). LncRNAs make up 80% of non-coding RNAs, although their roles are unknown (Gibb et al., 2011). LncRNAs spatio-temporal expression characteristics in embryonic and adult tissues make them functionally relevant (Morán et al., 2012; Kaushik et al., 2013; Ramos et al., 2023). Several studies have shown the role of lncRNAs in tissue/organ development and differentiation, including “Megamind” in the brain (Ulitsky et al., 2011), “Tie-1-AS” in vascular endothelium (Li et al., 2010), and “Fendrr” and “Braveheart” in heart development and function (Grote et al., 2013; Klattenhoff et al., 2013). Several recognized associations of lncRNAs in the regulation of tissue homeostasis (Ounzain et al., 2013; Shore and Rosen, 2014), stress response (Amaral et al., 2013), metabolism (Kornfeld and Brüning, 2014) and apoptosis (Rossi and Antonangeli, 2014) underscore their possible roles in diverse pathophysiological states affecting vital organs such as the heart. For example, lncRNA regulates Ca^2+^ homeostasis in cardiac tissue (Anderson et al., 2015).

Cardiac arrhythmia affects >2% of individuals in community-dwelling adults (Khurshid et al., 2018). Intracellular Ca^2+^ homeostasis is a key determining factor for initiating the rhythmic contraction and generation of synchronized beating patterns of the heart (Sejersted, 2011). In mammalian cardiomyocytes, Ca^2+^ initiates the calcium-induced calcium release (CICR) via *ryanodine* receptors (*RyR*) in the sarcoplasmic reticulum (SR) (Fabiato, 1983). The basic aspects of excitation-contraction coupling (ECC) via CICR are conserved in teleosts such as zebrafish (Llach et al., 2011). In zebrafish cardiomyocytes, SR-mediated Ca^2+^ release plays a minor role in ECC in the zebrafish heart (Bovo et al., 2013). LTCCs function as main gates for Ca^2+^ transient and mediate 80% of the Ca^2+^ entry during ECC in zebrafish cardiomyocytes (Bovo et al., 2013) in contrast to mammalian cardiomyocytes, where 70% Ca^2+^ transient is due to SR mediated release. Compared to human cardiomyocytes, contraction recedes faster in zebrafish (Zhang et al., 2011). Besides CICR, Na^+^-Ca^2+^-exchange plays a major role in ECC in the zebrafish heart (Nemtsas et al., 2010). Current knowledge of intracellular Ca^2+^ transients in zebrafish ECC is lacking. Thus, understanding zebrafish calcium handling systems is crucial to understanding vertebrate cardiac Ca^2+^-based ECC control evolution (Nemtsas et al., 2010).

Zebrafish have been explored to study cardiac diseases (Gut et al., 2017; González-Rosa, 2022; Zhang and Zhou, 2023) as their heart show strikingly similar cardiac physiology to humans (Bakkers, 2011). Adult zebrafish models for human cardiomyopathies have been successfully generated (Ding et al., 2020). Virus or transposon based Insertional mutagens such as GBT, coupled with phenotype based forward genetic screen are powerful tools for identifying new genes and transcripts in zebrafish (Clark et al., 2011). A recent study has reported a GBT based protein trap library in zebrafish and discovered essential genes for heart rhythm disorders (Ding et al., 2022).

We carried out a gene-breaking transposon (GBT)-based insertional mutagenesis screen as described in a earlier study (Sivasubbu et al., 2006) and identified 50 GBT trapped lines. One of the GBT lines displayed cardiac characteristics including, arrhythmia and an enlarged heart chamber phenotype, hence we named the gene trap insertional mutant as bigheart (*bh* ^*−/−*)^. The GBT insertion in bigheart fish maps to the intron 2 of *grin2bb* gene in reverse orientation in zebrafish. *Grin2bb* (*NR2B*) is one of the subunits of the NMDA receptor (NMDAR) and is described to form and function as a calcium-permeable ion channel (Strack et al., 2000; Koronyo-Hamaoui et al., 2007). The *Grin2b* subunit of NMDARs is reported to express in the heart tissue of several mammalian species (Gill et al., 1998; Seeber et al., 2000; Makhro et al., 2016). In rat, *Grin2b* subunit abundantly expresses in the heart tissue and forms a complex with ryanodine receptor *Ryr2* (Seeber et al., 2004). The cardiac NMDARs are reported to be actively involved in the regulation of heart rate (Makhro et al., 2016).

Using RNA sequencing data from previous studies (Singh et al., 2016), we observed an independent transcript at the gene trap insertion locus. We named this novel transcript as *grin2bb Associated RNA Transcript* (*grin2bbART*)*.* Further, we found that *grin2bbART* fulfills most of the characteristic non-coding signatures, and our data collectively advocates that *grin2bbART* might be a putative long non-coding RNA. We hypothesize that the gene trap-mediated disruption of the *grin2bbART* results in calcium flux aberrations, causing the *bigheart* phenotype. We propose that *grin2bbART* may have a potential role in cardiac rhythm function by regulating calcium flux in the zebrafish heart. Analyzing the patterns and specific distributions of coding and non-coding RNAs at the single-cell level during development will further provide insights into previously undiscovered regulatory processes of *grin2bb* and *grin2bbART* in cardiac development and may contribute to more effective therapeutic targeting strategies in the future (Ramos et al., 2023).

## Results

### Isolation and genetic characterization of the *Bigheart* mutation

**FIGURE 1:**
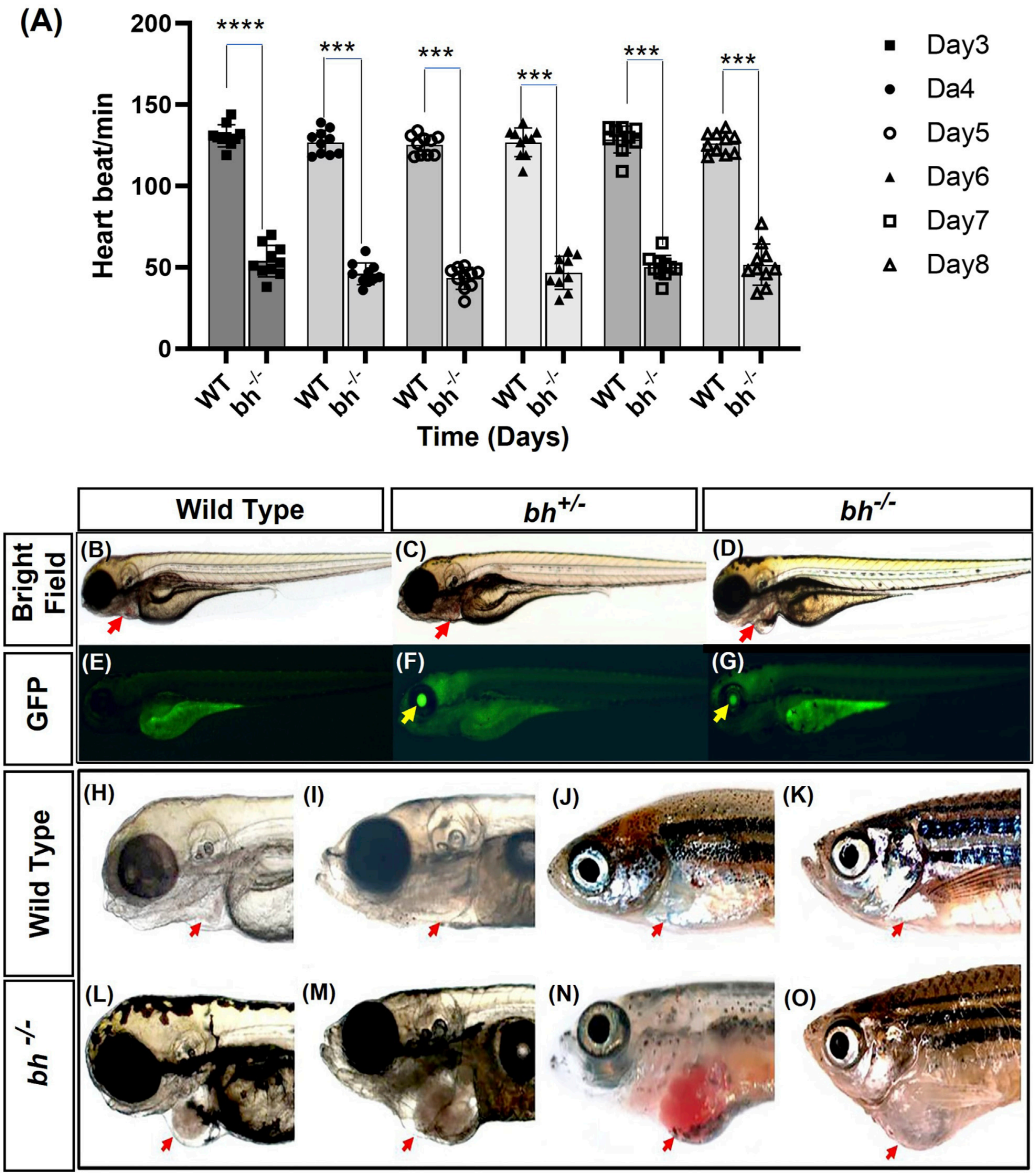
Heartbeat rate analysis and phenotypic characterization of *bigheart* mutant. **(A)** Heartbeat rate analysis in wild type (*bh* ^*+/+*^) and *bigheart* mutant fish (*bh* ^*−/−*^) fish. X-axis represents time in days, and Y-axis represents heartbeat per minute. **(B–O)** Phenotypic characterization of *bigheart* mutant. **(B–D)** represent bright field images of 3 dpf wild type (WT) embryos, heterozygous embryos (*bh* ^*+/−*^), and *bigheart* homozygous (*bh* ^*−/−*^) mutant embryos, respectively. **(E–G)** represent respective fluorescence images at 3 dpf. (Images were captured at ×2.5). Yellow arrowheads indicate GFP expression in the eye and red arrowheads indicate location of the heart. **(H,J,L,N)** represent WT fish at 3 dpf, 15 dpf, 90 dpf and 9 months. **(I,K,M,O)** represent corresponding images of the *bh* ^*−/−*^ fish at 3 dpf, 15 dpf, 90 dpf, and 9 months, respectively (***, *p* < 0.001, ****, *p* < 0.0001). The bars indicate the average values ± SD.

We performed an insertional mutagenesis screen in zebrafish using a Gene breaking Transposon (GBT) vector (Sivasubbu et al., 2006; Petzold et al., 2009) (Supplementary Figure S1). 50 GBT lines were generated, out of which 03 lines were cardiac mutants (Supplementary Table S1). We characterized one of the GBT lines that displayed a reduced heartbeat rate by 2 dpf (days post fertilization), followed by arrhythmia or irregular heartbeat (Figure 1A; Supplementary Movies SM1, SM2). In addition, we observed an enlargement of the heart in putative mutant embryos when compared to the age-matched wildtype embryos (Figures 1B–D; Supplementary Movies SM1–SM3). We named the mutation as “*Bigheart*” (*bh*). Quantitative analysis of the segregation of *bigheart* phenotype by pair mating of *bigheart* heterozygous fish (*bh* ^*+/−*^) was performed. A total of 631 embryos from four pair-wise crosses of *bh* ^*+/−*^ fish were screened for gene trap-linked green fluorescent protein (GFP) expression. We identified 449 (71%) embryos exhibiting GFP expression and 182 embryos (29%) without GFP expression, indicating segregation of the gene trap-linked GFP expression in a near Mendelian ratio (Figures 1B–G). Within the pool of 449 GFP-expressing embryos, we further screened for embryos that displayed *bigheart* phenotype. We noticed that 98 (16%) embryos displayed arrhythmia and an enlarged heart phenotype in the putative mutant *bh* ^*−/−*^ zebrafish (Figures 1D, G). Multiple matings between heterozygous (*bh* ^*+/−*^) fish and the wild-type fish resulted in half (50%) of the progeny displaying an identical gene trap-derived GFP expression, whereas the other half (50%) exhibited no detectable GFP expression. This data suggested that the *bigheart* line carries a single insertion.

### Irregular heartbeat in *bigheart* (*bh* ^*−/−*^) fish

We manually quantified the number of heart beats per minute from 3 dpf to 8 dpf in wildtype sibling and putative *bh* ^*−/−*^ mutant embryos. The mutant embryos displayed slow heartbeat rates starting at early 2 dpf. The heart in putative *bh* ^*−/−*^ embryos were observed to beat in the range of 36 ± 11.4 beats/min to 66 ± 11.4 beats/min as evaluated against the age-matched wild-type embryo, which displayed a count of 120 ± 6.6 beats/min to 150 ± 6.6 beats/min (Figure 1A).

**FIGURE 2:**
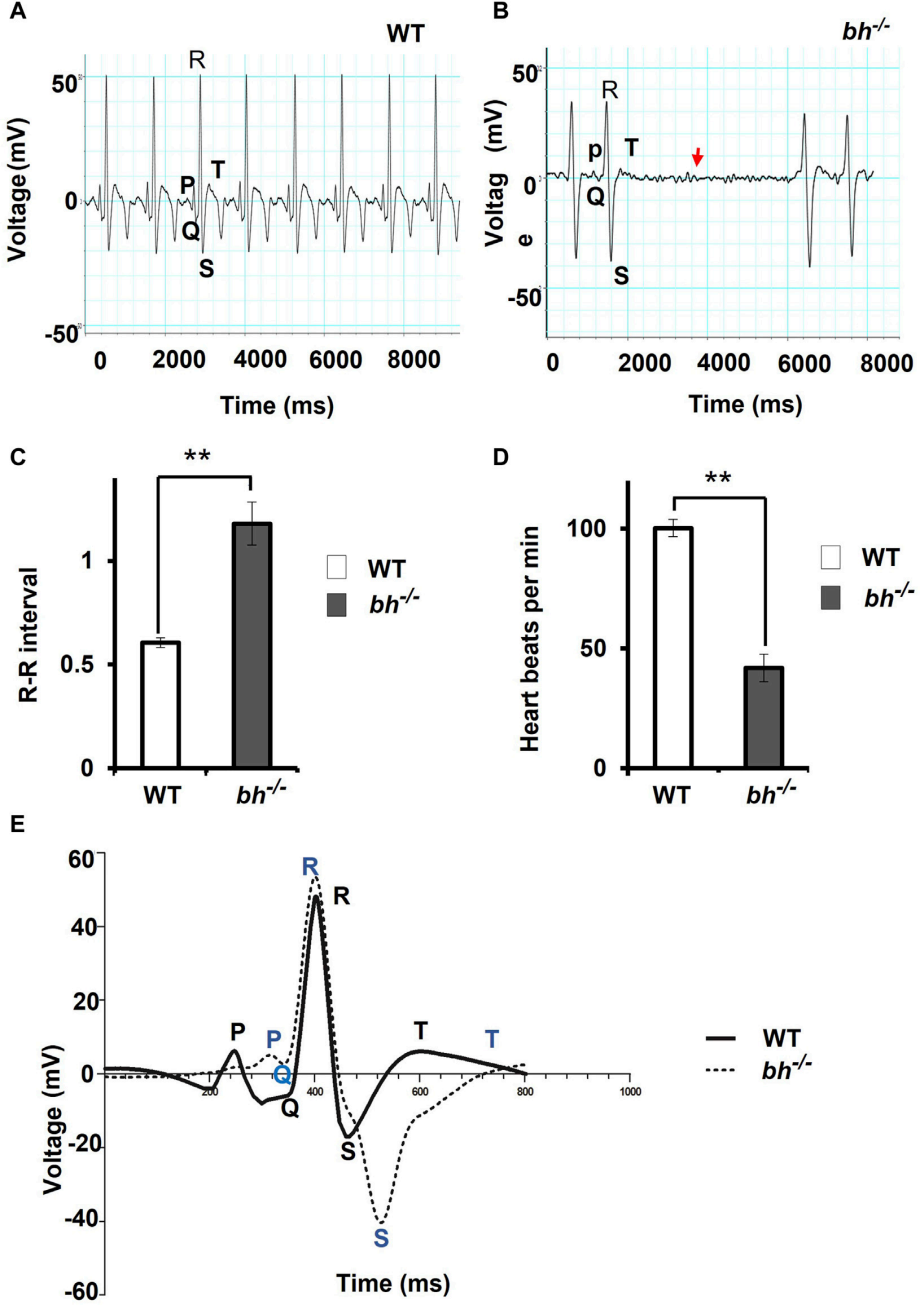
The electrocardiogram profile of *bigheart* fish indicates signs of arrhythmia. **(A)** Electrocardiogram recordings of wild-type (*bh* ^*+/+*^) zebrafish. X-axis represents time in milliseconds (ms), and Y-axis represents voltage in millivolts (mV). **(B)** Electrocardiogram recording of *bh* ^*−/−*^ fish, red arrowhead represents the absence of beats. **(C)** Graph representing the R-R interval in wild type and *bh* ^*−/−*^ fish. **(D)** Graph representing heart beat rate in adult wild type (*bh* ^*+/+*^) zebrafish and *bh* ^*−/−*^ fish. **(E)** Graph showing an overlay of wild type and *bh* ^*−/−*^ mutant ECG recordings (Regular line and dashed line represents the ECG profiles of wild type and *bh* ^*−/−*^ fish, respectively). P, Q, R, S and T represent regular ECG waves. (**, *p* < 0.01). The bars indicate the average values ± SD.

The majority of the putative *bh* ^*−/−*^ embryos displayed persistent arrhythmia and chamber enlargement and survived to adulthood (Figures 1H–O). To assess the heart function, we obtained electrocardiogram (ECG) profiles of the wild type fish and putative *bh* ^*−/−*^ fish at 9 months of age (Figure 2). The ECG profile of age-matched wild type *and* putative *bh* ^*−/−*^ animals exhibited major differences (Figures 2A, B). The R-R interval, which represents the time measurement between two adjacent R waves of a heartbeat, was discerned to be delayed in the putative *bh* ^*−/−*^ fish as compared to the wild-type (Figure 2C). The number of beats per minute was reduced by 60% in the putative *bh* ^*−/−*^ fish when compared to wild type (Figure 2D). The S wave, which signifies the end point of ventricular depolarization, exhibited a negative value for the putative *bh* ^*−/−*^ fish heart (Figure 2E). ECG recordings for the putative heterozygous *bh* ^*+/−*^ siblings revealed standard cardiac electrical profile. A significant observation in the 3 dpf putative *bh* ^*−/−*^ embryos was the heart ceased to beat for a time interval ranging from 10 s to a minute (Supplementary Movie SM3) and resumed the rhythm after the pause, exhibiting an arrhythmic condition. The quantitative measurements of cardiac electrical function in the putative *bh* ^*−/−*^ fish indicate that the gene trap insertion is associated with a distinctive arrhythmia phenotype.

### Cardiac chamber enlargement in *bigheart* (*bh* ^*−/−*^) fish

**FIGURE 3:**
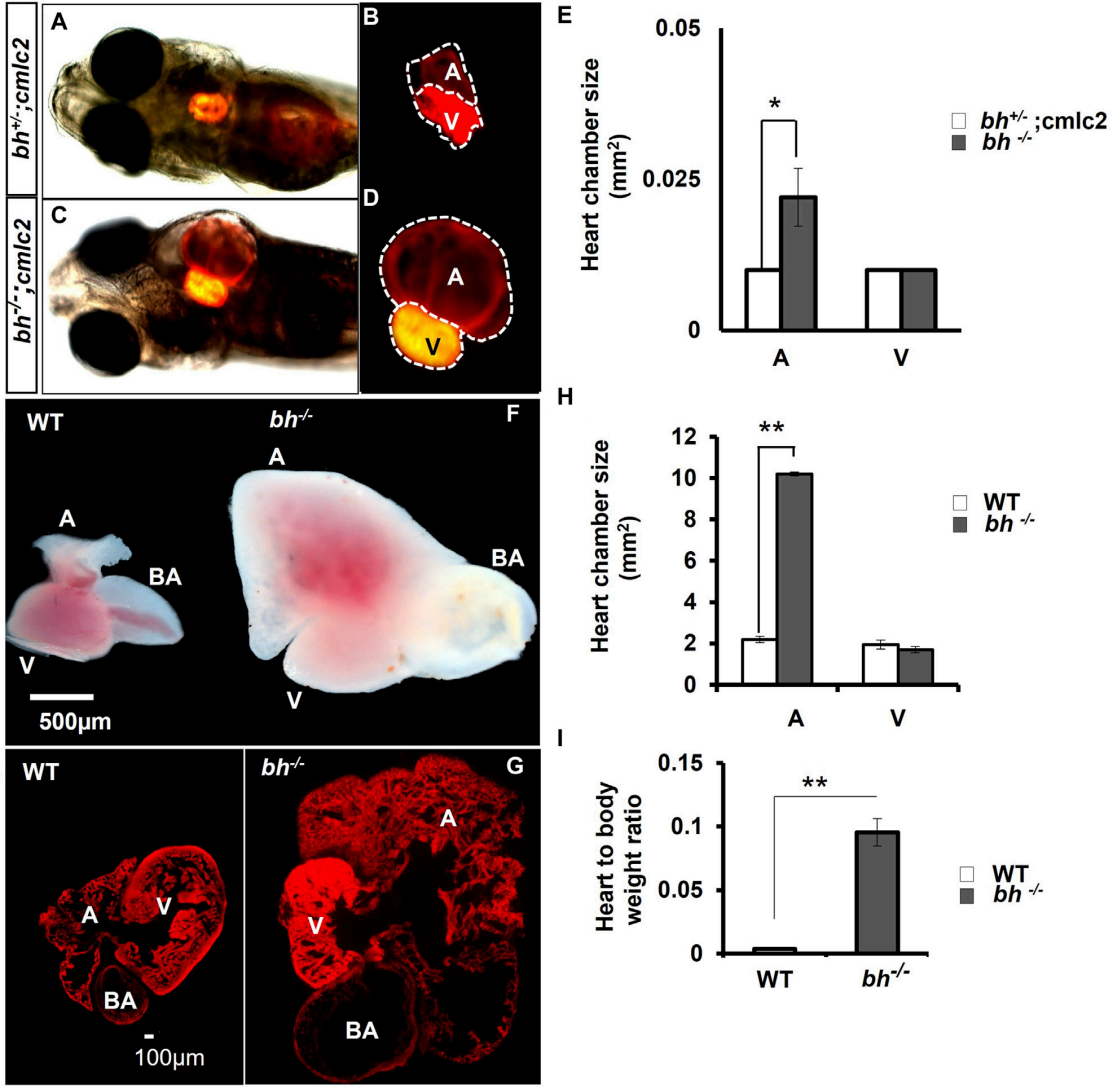
Anatomical study of *bh* ^*−/−*^ *;myl7: RFP* double transgenic reveals atrial enlargement in *bigheart*. **(A,B)** 5dpf double transgenic (*bh* ^*+/−*^; *myl7*) *fish* embryo with red fluorescence protein expression in the normal heterozygous siblings heart (*bh* ^*+/−*^). **(C,D)** *Bigheart* mutant (*bh* ^*−/−*^; *myl7*) embryo with red fluorescence protein expression in the heart. **(B,D)** Zoomed images of the heart in transgenic, *bh* ^*+/+*^; *myl7* and *bh* ^*−/−*^; *myl7* embryos (Images were captured at ×5 magnification using Zeiss Axio-observer 40 microscope). **(E)** Heart chamber size measurement in wild type and mutant embryos; the X-axis represents heart chambers (A: atrium; V: ventricle, BA: bulbus arteriosus); Y-axis represents the heart chamber size in mm^2^. **(F)** Dissected wildtype and mutant (bh^−/−^) 9 months old adult heart. **(G)** Heart tissue section of wild type and mutant (*bh* ^*−/−*^) stained with phalloidin texas red showing enlarged atrium and dilated bulbus arteriosus. **(H)** Graph representing the heart chamber size in WT and *bh* ^*−/−*^ fish corresponding to **(F)**. **(I)** Heart to body weight ratios for WT and *bh* ^*−/−*^ mutant fish indicating enlarged heart of the mutant as compared to WT. (A: atrium; V: ventricle; BA: bulbus arteriosus). (Images were captured at ×2.5) (and **, *p* < 0.01). The bar indicates the average values ± SD.

We generated double transgenic *bh* ^*−/−*^; *Tg* (*myl7:RFP*) fish by crossing the putative *bh* ^*+/−*^ fish with *Tg* (*myl7:RFP*) fish (Supplementary Figure S2). The *Tg* (*myl7:RFP*) line was generated in our laboratory as described in the Supplementary Material: extended method, (Generation of Myl7 transgenic line). The *Tg* (*myl7: RFP*) fish expresses red fluorescent protein (RFP) under the control of a heart-specific *myl7* gene promoter and thus aids easy morphological observation of cardiac chambers (Figures 3A, B). The putative mutant bh^−/−^ fish displayed an onset of heart enlargement at 2 dpf (Supplementary Movies SM2A–D), that becomes prominent at 3 dpf (Supplementary Movies SM3, SM4). We investigated the phenotype for the specific cardiac chamber(s) affected in *bh* ^*−/−*^;*Tg* (*myl7:RFP*) fish and observed distension of atrium in the putative *bh* ^*−/−*^ embryonic heart compared to the wildtype siblings at 5 dpf (Figures 3A–D and Supplementary Figures S2C, D). To measure the extent of atrial chamber distension, we quantified the increase in surface area by measuring the circumference of individual chambers in 5 dpf putative mutant *bh* ^*−/−*^ fish. The 5 dpf putative *bh* ^*−/−*^ embryos displayed two-fold enlargement in size of the atrial chamber compared to age-matched *Tg* (*myl7: RFP*) embryos (Figure 3E). Anatomical dissection of the putative *bh* ^*−/−*^ adult (9 months) heart further confirmed the enlargement of the atrium and outflow tract with no distinct change in the ventricular chamber (Figures 3F, G). Quantification of cardiac surface area in the heart of adult (9 months old) putative *bh* ^*−/−*^ fish revealed a five-fold enlargement of the atrium and a dilated *bulbus arteriosus* compared to age-matched wildtype (Figures 3G, H). Heart by body weight ratio, a standard analysis for assessing chamber enlargement, was calculated to be approximately ten-fold higher in *bh* ^*−/−*^ when judged against age-matched wild type fish (Figure 3I). O-dianisidine staining of 5 dpf larvae showed greater blood accumulation in the *bh* ^*−/−*^ as compared to age matched siblings (Supplementary Figures S3A–D). Chamber enlargement and the blood accumulation observed in the putative *bh* ^*−/−*^ fish gradually progressed with age as evaluated in the morphological analysis of the dissected heart (Figures 3F, G; Supplementary Figures S3E, F), and hematoxylin and eosin stained sections (Supplementary Figures S3G–J). The phalloidin staining of adult *bh* ^*−/−*^ fish heart tissue exhibited potential signs of hypertrophied muscle in the ventricle (Figure 3G; Supplementary Figure S4). Further, scanning electron Microscope (SEM) analysis in adult *bh* ^*−/−*^ revealed hypertrophied, disarrayed, overlapping, and abnormally branched cardiomyocytes in the ventricle (Supplementary Figure S5). Our data collectively uncovers an enlarged atrium and hypertrophic signatures in the cardiac ventricle of putative *bh* ^*−/−*^ fish.

### Gene trap insertion in the *Bigheart* line maps to the intron of zebrafish *grin2bb* subunit of NMDA receptor gene

**FIGURE 4:**
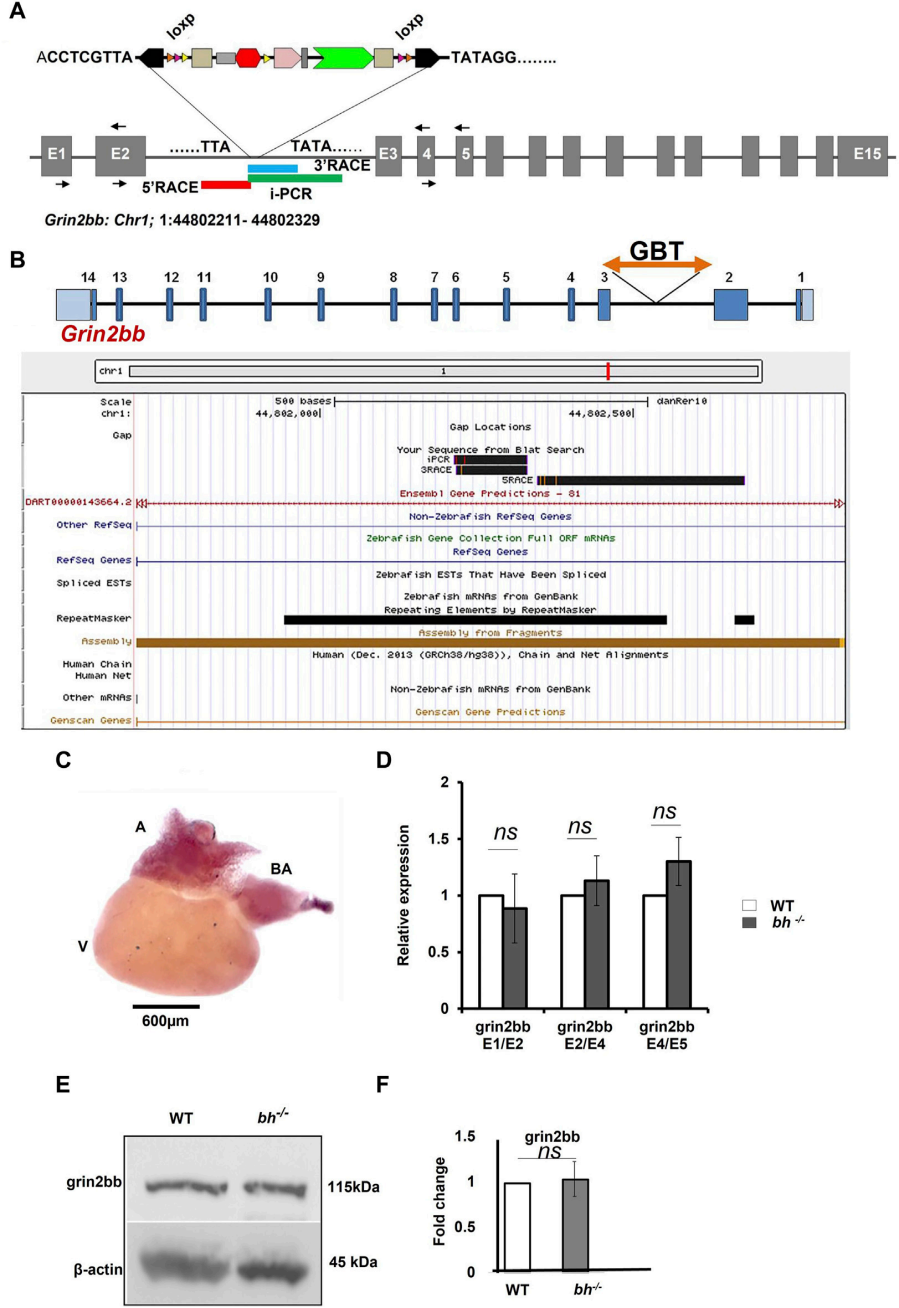
*grin2bb* transcript and protein levels are unaffected in *bigheart*. **(A)** pGBT-PX integration in bigheart maps to intron 2 of *grin2bb* gene located on the reverse strand of Chromosome 1. Box in red indicates the 5′RACE mapped region, blue represents the 3′ RACE mapped region, and the green represents the *i*PCR mapped region. **(B)** Screen shot of UCSC genome bowser showing the *grin2bb* transcript and the bigheart GBT insertion loci in the intron 2. **(C)** Expression profiling of *grin2bb* transcript in 9-month-old adult WT heart tissue using *in situ* hybridization. **(D)** qRT PCR showing relative expression of *grin2bb* partial transcript in sibling wildtype and *bh* ^*−/−*^ heart tissue. **(E)** Western blot analysis of *grin2bb* in WT and bh^−/−^ heart tissue. *β-actin* was used as internal control. **(F)** Quantification showing Grin2bb protein fold change in the WT and bh^−/−^. (black arrows in A indicate the regions where the primers were designed. The RT PCR and western blot experiments were repeated three times. The bar indicates the average values ± SD.

Inverse PCR (iPCR) analysis of the putative *bh* ^*−/−*^ fish mapped the gene breaking trap insertion to the second intron of zebrafish *glutamate receptor, ionotropic, N-methyl D aspartate 2B* (*grin2bb*: Gene ID: 559976) gene located on the reverse strand of chromosome 1 (Figures 4A, B; Supplementary Figure S6). We analyzed the insertion locus affected in the *bh* ^*−/−*^ fish using 3′and 5′ RACE (Rapid amplification of the complementary cDNA ends). The PCR products obtained from 3′RACE (118 bp) and 5′RACE (453 bp) analysis mapped to the intron 2 of *grin2bb* gene and overlapped with the iPCR sequence that was obtained previously (Figures 4A, B and Supplementary Figures S7, S8).

### Antisense morpholino-mediated Grin2bb knockdown does not display a bigheart phenotype

Our preliminary RNA seq analysis shows that the *grin2bb* transcript is downregulated (fold change = −1.3, Supplementary Table S3). Further, we examined the expression of the *grin2bb* transcript in the wild type adult zebrafish. A 280 bp probe was designed using primers mapping to exon1 and 2 of the *grin2bb* gene. The *grin2bb* transcript expressed uniformly in the entire atrium and *bulbus arteriosus* (BA) in the heart of 9 months old wild type zebrafish (Figure 4C). Next, we investigated if changes in the *grin2bb* gene transcript or protein contribute to the cardiac specific defects observed in the *bh* ^*−/−*^ fish. We measured the *grin2bb* transcript levels in the heart tissue of *bh* ^*−/−*^ as well as wild type fish using qRT-PCR (Figure 4D). We could amplify PCR products spanning splice events between exon 1 and 2; exon 2 and 4; exon 4 and 5 from the *grin2bb* transcript in the heart tissue of *bh* ^*−/−*^, as well as age, match wild type fish. We did not observe any significant difference in the *grin2bb* transcript levels in the cardiac tissue between the *bh* ^*−/−*^ *and* age-matched wild-type fish. Using Western blot analysis, we measured Grin2bb protein levels in the heart tissue of *bh* ^*−/−*^ and the wild-type fish. Grin2bb protein expression in the heart tissue of *bh* ^*−/−*^ fish was comparable to that of wild-type fish (Figures 4E, F). Next, we injected wild-type zebrafish embryos with both translation blocking (ATG) and splice site-blocking morpholino oligonucleotides (MO) that would target exon 2 and exon 3 of the grin2bb transcript (MO sequences are described in Method). Interestingly, the splice MO-injected embryos did not yield the *bh* ^*−/−*^ phenotype (Supplementary Figure S9), but the Grin2bb translation blocking MO at a higher doses of 6 nL and 100 µM did showed a severe cardiac defect (Supplementary Figure S9) but did not resemble the *bh* ^*−/−*^ phenotype, suggesting that the grin2bb protein-coding gene may not be associated with the phenotype observed in the *bh* ^*−/−*^ fish.

### Gene trap insertion in the *bigheart* fish maps to a putative RNA transcript in the intron 2 of zebrafish *grin2bb* subunit of NMDA receptor gene

In our RT PCR analysis and western data grin2bb was not showing any significant, we next focussed on the antisense transcript. We next investigated the nature of the transcript obtained using RACE analysis at the insertion site in *bh* ^*−/−*^ fish. We obtained a partial transcript with a sequence length of 550 bp using RACE. We speculated that the 550 bp sequence may represent a putative novel unrecognized transcript transcribed from the *grin2bb* gene locus.

**FIGURE 5:**
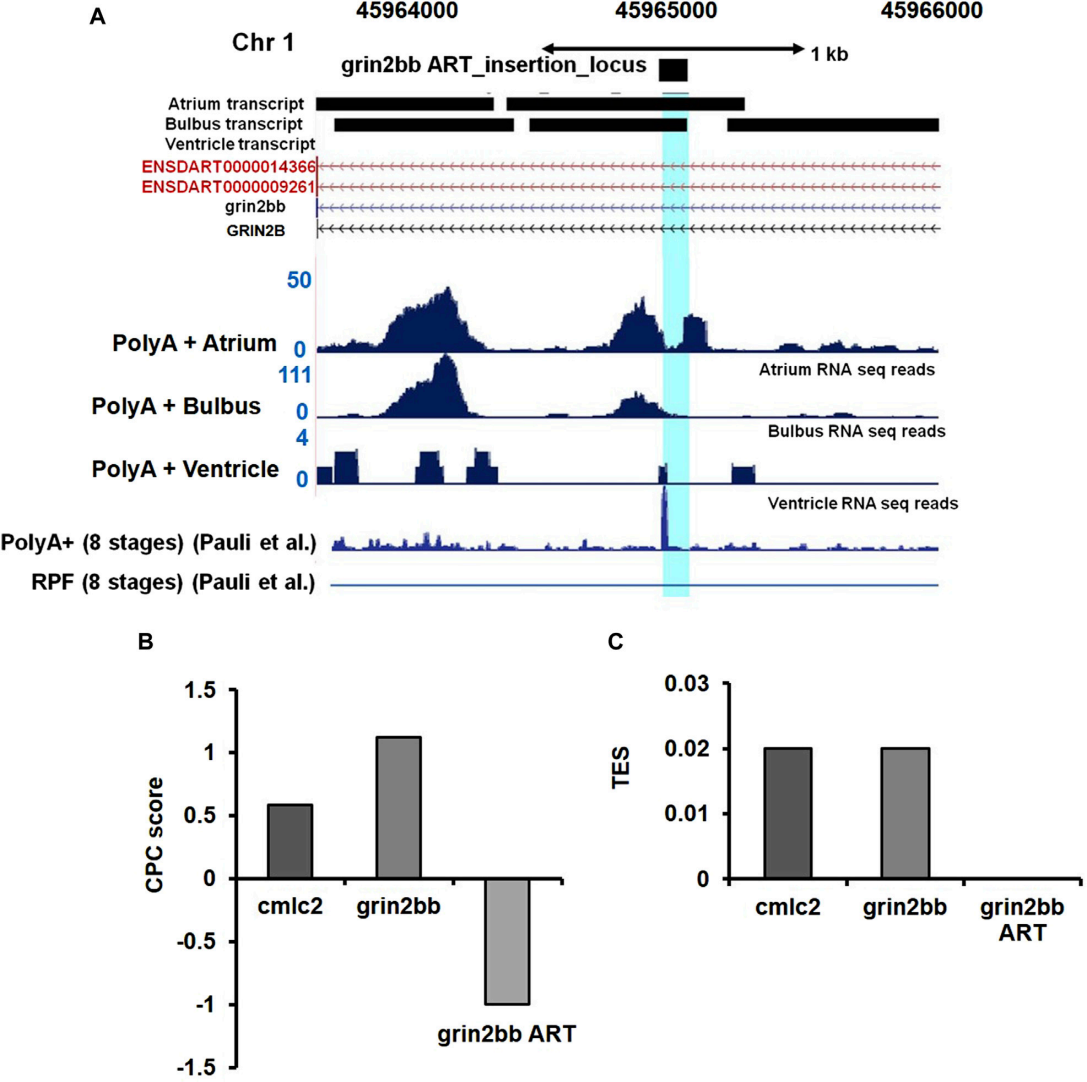
Ultra-deep RNA sequencing of WT heart suggests the presence of unannotated transcript in *i*PCR mapped region. **(A)** Snapshot displaying the *grin2bbART* locus across the three cardiac chambers in zebrafish. RNA sequencing and ribosomal profiling reads have been aligned and mapped onto the *grin2bbART* locus. **(B)** Graph representing coding potential score of *grin2bbART* as well as *grin2bb* and *myl7* transcripts. **(C)** Translation efficiency score (TES) score of *grin2bbART*, *grin2bb,* and *myl7* transcripts.

To validate our speculation, we analyzed high-resolution RNA sequencing data (RNA-seq) of the three cardiac chambers in zebrafish heart *viz;* atrium, ventricle, and *bulbus arteriosus* (Singh et al., 2016). The RNA-seq data set from this study was further processed to identify lncRNA transcripts using a computational pipeline previously developed in our laboratory (Kaushik et al., 2013). Analysis of RNA-seq read coverage around the gene trap insertion site suggested active transcriptional activity. The RNA-seq data from cardiac chambers revealed the presence of transcripts at the gene trap insertion site and suggested transcript enrichment in the atrium and *bulbus arteriosus* (Figure 5A). Trace transcript signature was detected in the ventricular chamber (Figure 5A). RNA-seq data analysis strongly supports the possibility that the gene trap integration in the *bigheart* fish line disrupts a putative novel transcript resulting in the *bigheart* phenotype. Henceforth, we refer to this putative novel transcript as “*grin2bb Associated RNA Transcript*” (*grin2bbART*).

### *Grin2bbART* encodes for a potential long non-coding RNA

We further examined the Ribo-Seq data from published zebrafish studies (Chew et al., 2013). Ribo-Seq read coverage in the region around the insertion site was found to be absent, suggesting a compelling evidence for the lack of coding potential of the *grin2bbART*. Previous studies have also supported the presence of polyadenylation signatures at the insertion site (Pauli et al., 2012) (Figure 5A). We applied “coding potential calculator” software calculations and predicted a negative score for *grin2bbART*, indicating a non-coding potential (Figure 5B). We calculated the translational efficiency scores (TES) for *grin2bb ART* with *myl7* transcript as a positive control (Figure 5C). The TES scores indicated the absence of ribosomes on *grin2bbART* whereas *myl7* and *grin2bb* were found to be translated. Thus, *grin2bbART* is not translated in wild-type zebrafish heart. The analysis of chamber-specific cardiac transcriptome and information obtained from investigation of previously reported studies makes it provocative to argue that grin2bbART encodes for a potential long non-coding RNA that is transcribed from the intron of grin2bb gene and is expressed in the atrium and bulbus arteriosus of the wild type zebrafish heart.

### *Grin2bbART* has a chamber-specific expression in zebrafish heart

**FIGURE 6:**
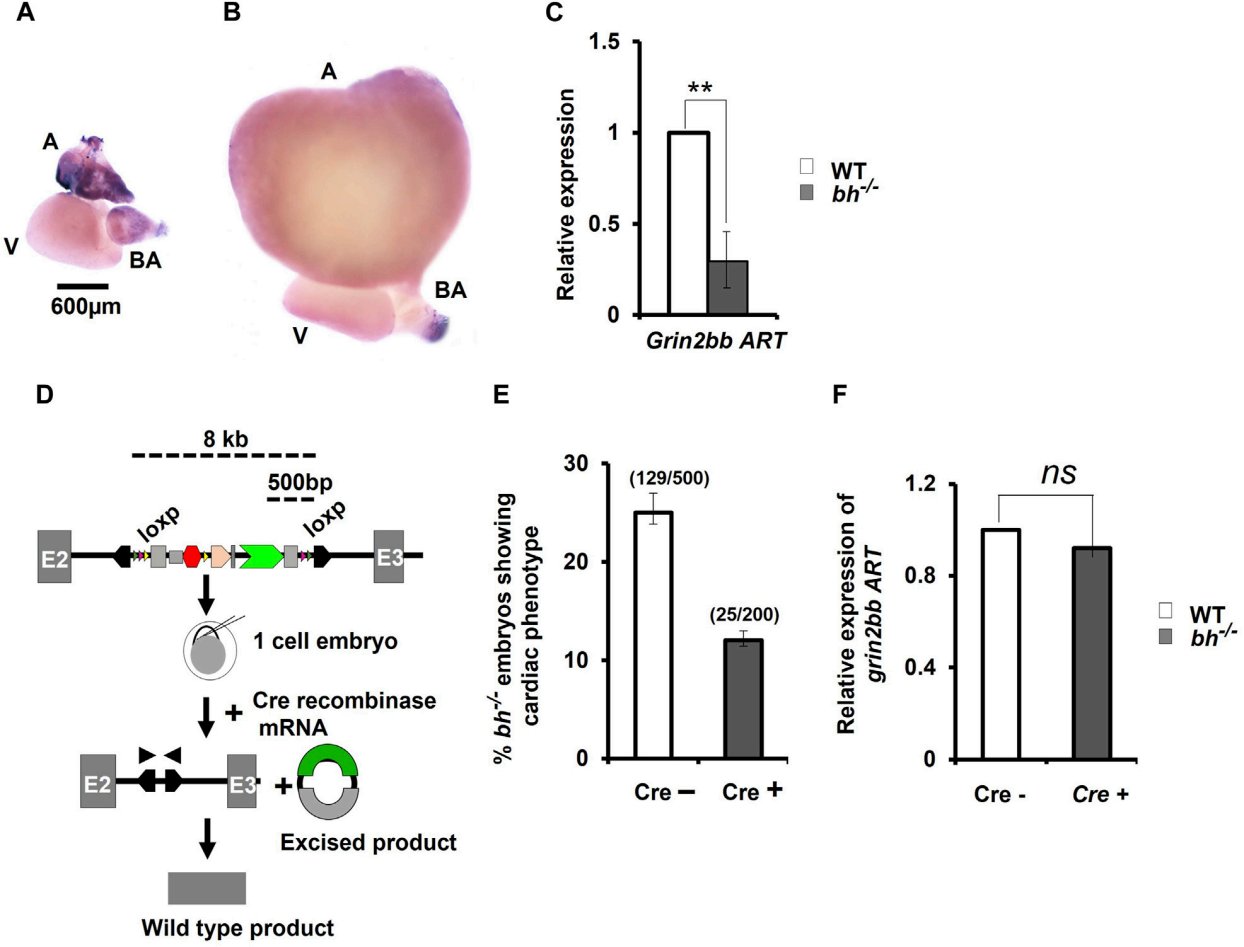
*Cre*-recombinase restores wild type expression of *grin2bbART* in *bigheart* mutant. **(A)** *Expression profiling of grin2bbART transcript in adult zebrafish using whole mount in situ hybridization* **(B)** *grin2bbART* expression in *bh* ^*−/−*^ heart tissue (A: atrium; V: ventricle; BA: bulbus arteriosus) **(C)** qRT PCR analysis showing the relative expression of *grin2bbART* in WT and *bh* ^*−/−*^ heart tissues. **(D)** Schematic depicting *Cre-recombinase* mediated gene trap reversion in *bigheart* mutant. Triangles in pink represent the *loxP* sites flanking the gene trap. By supplying *Cre recombinase* mRNA, the mutagenicity cassettes and 3′ exon trap are excised. **(E)** *Cre recombinase* reverts the *bigheart* phenotype to wild type, approximately in 50% of the injected embryos, compared to non-injected control. **(F)** Relative expression of *grin2bbART* in *Cre recombinase* injected WT and *bh* ^*−/−*^ embryos. Black arrowhead shows positions of primers of the PCR analysis of Cre recombinase injected and non-injected *bh* ^*−/−*^ embryos. *N* = 5 heart samples were analyzed in each group for the *in-situ* experiment. The RT-PCR experiments were repeated three times in triplicate reactions. (**, *p* < 0.01). The bars indicate the average values ± SD.

**FIGURE 7:**
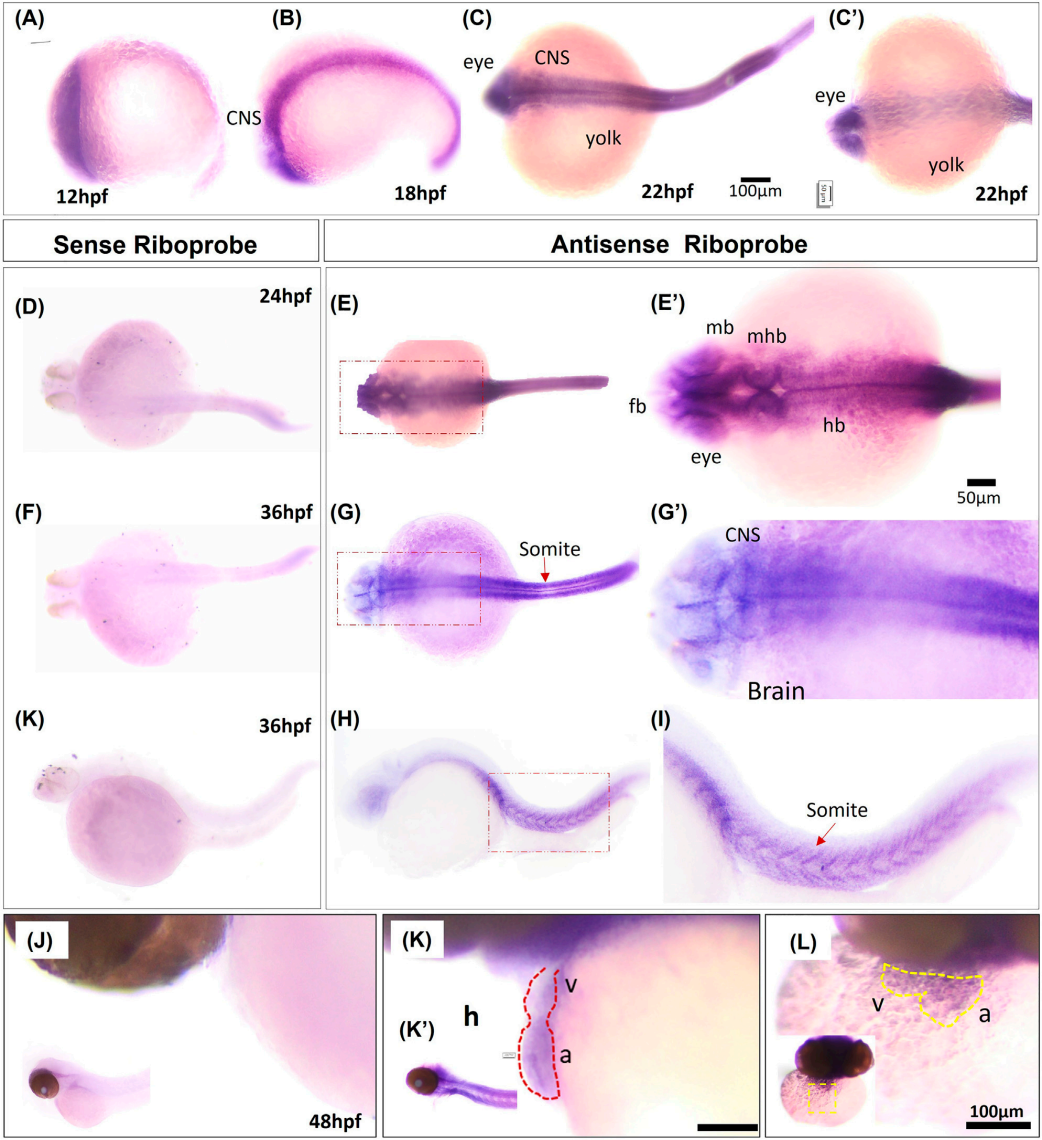
Whole mount *in situ* hybridization of grin2bbART mRNA in zebrafish embryos. **(A–C)** Representative embryos showing the expression of *grin2bbART* mRNA probe at **(A)** 12 hpf, **(B)** 18 hpf, **(C)** 22 hpf dorsal view and **(C′)**. 22 hpf ventral view. **(D,F,H)** grin2bbART sense probe showing the absence of grin2bbART signal at 24 hpf, 36 hpf. **(E)** Dorsal view of 24 hpf showing grin2bbART mRNA expression in the CNS. **(E′)** enlarged image showing the region in red dotted box in **(E)**. **(G)** Dorsal view of 36 hpf embryo showing grin2bbARTexpression in the CNS, somites and myotomes. **(G′)** enlarged image showing the region in red dotted box in **(G)**. **(I,I′)** grin2bbART expression in the somites. I′ shows the enlarged view of red box in **(I)**. **(J)** Lateral view of the 48 hpf embryos anterior region probed with the sense riboprobe showing the absence of grin2bbART expression. **(K)** Lateral view showing grin2bbART expression in the brain and heart of 2 dpf embryo. K′ shows expression in the CNS. **(L)** Ventral view of the anterion head region of 48 hpf embryo showing grin2bbART expression in the heart. fb, forebrain; mb, mid brain; hb, hind brain; mhb, mid brain hin brain boundary; h, heart; V, ventricle; a; atrium; CNS, Central Nervous system. The experiment was repeated atleast two times and *n* = 10 animals were analyzed per time points.

To confirm RNA-seq results for *grin2bbART*, we performed whole mount *in situ* hybridization (WISH) to analyze the distribution of *grin2bbART* across cardiac chambers in adult zebrafish hearts. A 250 bp probe was designed upstream of the gene trap insertion site in the region to which *grin2bbART* was mapped. *Grin2bbART* expression was detected in the atrium and *bulbus arteriosus* in the heart of nine-month-old wild type zebrafish (Figure 6A). The expression was observed to be higher in the atrium than in the BA. However, the expression was nearly absent in the ventricular chamber (Figure 6A). The *in situ* expression data suggested a chamber-restricted expression of *grin2bbART* in the wild type adult heart. The expression of *grin2bbART* was observed to be at its minimum in the *bh* ^−/−^ mutant fish heart with trace expression in the atrium and BA (Figure 6B). We carried out a qRT-PCR analysis to measure the expression levels of *grin2bbART* in wild type and *bh* ^*−/−*^ mutant hearts and noticed a four-fold reduction in the expression of *grin2bbART* in the *bh* ^*−/−*^ mutant fish (*p* < 0.01) (Figure 6C). The *in situ* hybridization and qRT–PCR data demonstrates that the *grin2bbART* is downregulated in the *bigheart* mutant fish. We previously examined and confirmed the expression of *grin2bb* gene in the wild type zebrafish heart tissue using RNA sequencing, RT-PCR, and ISH (Figures 4B, C; Supplementary Table S3). Taken together, the expression profiles of *grin2bb* and *grin2bbART*, we conclude that *grin2bb* and *grin2bbART* exhibit overlapping expression domains in the adult zebrafish heart chambers. As the phenotype of cardiac arrhythmia and chamber enlargement arose as early as 2 dpf, it raises the question of how *grin2bbART* is expressed during development. To further study the expression of *grin2bbART* in the zebrafish development, we performed whole mount *in situ* hybridization (WISH) to analyze the distribution of *grin2bbART* across 12 hpf, 18 hpf, 22 hpf, 24 hpf, 36 hpf, 48 hpf, and 72 hpf of development. As shown in Figures 7A–C′ the antisense probe showed the expressions in the CNS, eyes, and the somites. The expression was detected in the CNS and somites or myotomes at 24 hpf, 36 hpf, 48 hpf, and 72 hpf (Figures 7E, E′, G, G′, I, I′ and Supplementary Figures S10B–G). The grin2bbART expression was also observed in the pectoral fins (Supplementary Figure S9F) in 72 hpf, and in the heart at 48 dpf and 72 dpf (Figures 7K, K′, L and Supplementary Figures S9B, C). The expression was not detected in case of the embryos labelled with the sense probe (Figures 7D, F, H, J; Supplementary Figure S10A).

### *Cre recombinase*-mediated excision of the gene trap reverts phenotype and restores *grin2bbART* expression in *bigheart* fish

We performed *Cre* reversion assay to study the effects of gene trap excision on the phenotype observed in the putative *bh* ^*−/−*^ mutant fish (Figure 6D). Pair-wise breeding of heterozygous *bh* ^*+/−*^ adult fish produced embryos that display the phenotype characterized by cardiac arrhythmia and hypertrophy. Injection of *Cre recombinase* (*Cre*) into the embryos resulting from pair-wise breeding of *bh* ^*+/−*^ heterozygous fish, reverted the cardiac phenotype in 50% of the animals (Figure 6E). PCR analysis of the *bh* ^*−/−*^; *Cre+* embryos was performed using primers specific to GFP and Tol2 IR (Supplementary Figure S10). Non-injected *bh* ^*−/−*^; *Cre-*embryos were used as controls. The expected PCR product was not obtained in case of the *bh* ^*−/−*^; *Cre+* embryos as detected in the case of control *bh* ^*−/−*^; *Cre-*embryos, indicating *cre-*mediated excision of the gene trap. We next measured the expression levels of *grin2bbART* in the *bh* ^*−/−*^; *Cre+* embryos and observed that the transcript levels for *grin2bbART* are comparable to that of WT;*Cre* ^*-*^ embryos (Figure 6F). The *Cre recombinase*-based reversion assay thus restored the wild type expression of *grin2bbART* while also abolishing the cardiac phenotype in *bigheart* mutant fish. Our results confirm that the putative *bh* ^*−/−*^ mutant fish carries a single copy of the gene trap vector that disrupts the putative novel transcript, grin2bbART, linked with the *bigheart* phenotype.

### *Grin2bbART* specific antisense morpholino phenocopies the *bigheart* mutation

**FIGURE 8:**
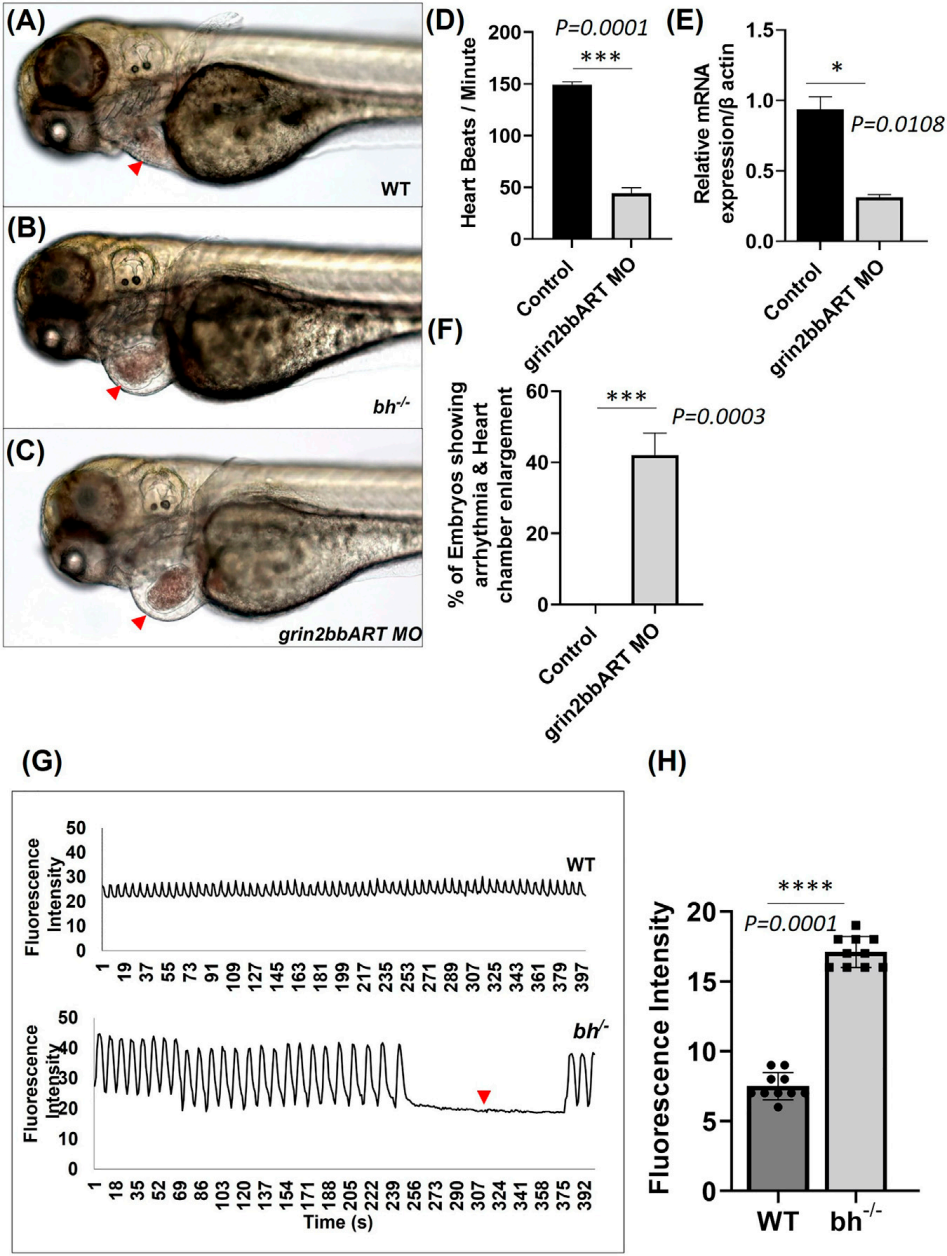
Big heart mutants displayed calcium mishandling and morpholino based knockdown of *grin2bbART* phenocopies the cardiac phenotype of *bigheart.* **(A)** Wild type zebrafish embryo at 3 dpf, **(B)** *bigheart* mutant at 3 dpf, **(C)** *grin2bbART* morphant at 3 dpf. **(D)** Graph representing heartbeat rate in the *grin2bbART* MO injected embryos. NIC, Non-injected control. Red arrowheads mark the heart. (Images were captured at ×5 magnification). **(E)** Ralative mRNA expression of grin2bbART after MO mediated grin2bbART knockdown. **(F)** Graph representing the percentage of embryos showing arrhythmia and chamber enlargement in the *grin2bbART* MO injected embryos. **(G)** Graph showing calcium signals in wildtype and *bh* ^*−/−*^ age-matched zebrafish. X-axis represents time in seconds. The Y-axis represents fluorescence intensity. The arrow highlights the signals that indicate the absence of calcium signal and heartbeat in *bh* ^*−/−*^ compared to WT. **(H)** Graph showing the fluorescence intensity in WT and *bh* ^*−/−*^ (*N* = 10 zebrafish were studied for heart analysis in each group). For calcium imaging *N* = 10, 2 dpf zebrafish embryos were analyzed. **, *p* < 0.01, ***, *p* < 0.001 and ****, *p* < 0.0001. The realtime RT PCR experiment was repeated atleast two time. The bars indicates the average values ± SD.

To find the function of *grin2bbART in wild-type zebrafish,* we performed antisense morpholino-mediated downregulation of *grin2bbART*. Wild type one-cell zebrafish embryos were injected with a cocktail of antisense morpholino oligonucleotides (MO) designed specifically for the 550 bp *grin2bbART* sequence. *Grin2bbART* MO-injected animals displayed cardiac chamber enlargement as observed in the putative *bh* ^*−/−*^ embryos (Figures 8A–C). The heartbeat rate in the *grin2bbART MO* injected animals was recorded to be ∼40 ± 11.2 beats/min while the wild type fish recorded a heartbeat rate of approximately 150–160 ± 6.6 beats/min (*p* = 0.0001, Figure 8D). About 45% of the *grin2bbART MO* injected animals displayed arrhythmia similar to that observed in the putative *bh* ^*−/−*^ fish (*p* = 0.0003) (Figure 8F). Real time RT PCR analysis confirmed the morpholino mediated *grin2bbART* knockdown in the morphants (Figure 8E). The grin2bbART antisense MO cocktail injected embryos displayed significant reduction (∼60%) in the mRNA expression of *grin2bbART* as compared to the control MO injected embryos (*p* = 0.0108) (Figure 8E). Thus, transient downregulation of the *grin2bbART* in the wild type zebrafish embryos produced phenocopies of the chamber enlargement and arrhythmia phenotype of the putative *bh* ^*−/−*^ mutant fish (Supplementary Movie SM5).

### *Bigheart* fish shows altered calcium handling

The genomic association of *grin2bbART* with *grin2bb* protein-coding gene, a subunit of a calcium ion transport receptor channel, led us to probe calcium activity in the *bh* ^*−/−*^ mutant embryos. We designed our experiment on two key rationales; the first being overlapping expression profiles of *grin2bbART* and *grin2bb* in wild-type zebrafish heart, and the second- Ca^2+^ handling defects are associated with arrhythmia (Pogwizd et al., 2001; van Oort et al., 2010; Gardner et al., 2015), as seen in putative *bh* ^*−/−*^ fish. Thus, we conjectured that Ca^2+^ handling might be affected in the putative *bh* ^*−/−*^ mutant fish. To monitor calcium transients *in vivo*, 2 dpf stage wild type and *bh* ^*−/−*^ embryos were injected with Calcium Green-1 Dextran. High-speed two-dimensional confocal calcium imaging was used to analyze calcium transients in wild-type and *bh* ^*−/−*^ mutant hearts (Supplementary Movies SM6, SM7). Each heartbeat was accompanied by a synchronized wave of calcium-induced fluorescence in the wild-type embryos. However, in the *bh* ^*−/−*^ embryos, an unsynchronized fluorescence signal was observed (Figures 8G, H). Further, no calcium signals were recorded for the time interval whenever the heartbeat pauses in the *bh* ^*−/−*^ mutants as indicated by the red arrowhead (Figure 8G). The data demonstrates abnormal calcium transients in the heart of *bh* ^*−/−*^ *embryos* and the absence of calcium-mediated fluorescence signal in the absence of heartbeat. Thus, it appears that the arrhythmic phenotype of the *bigheart* mutant fish may be associated with alterations in Ca^2+^ handling.

### *Bigheart* cardiac transcriptome revealed differential expression of Ca^2+^ homeostasis genes

**FIGURE 9:**
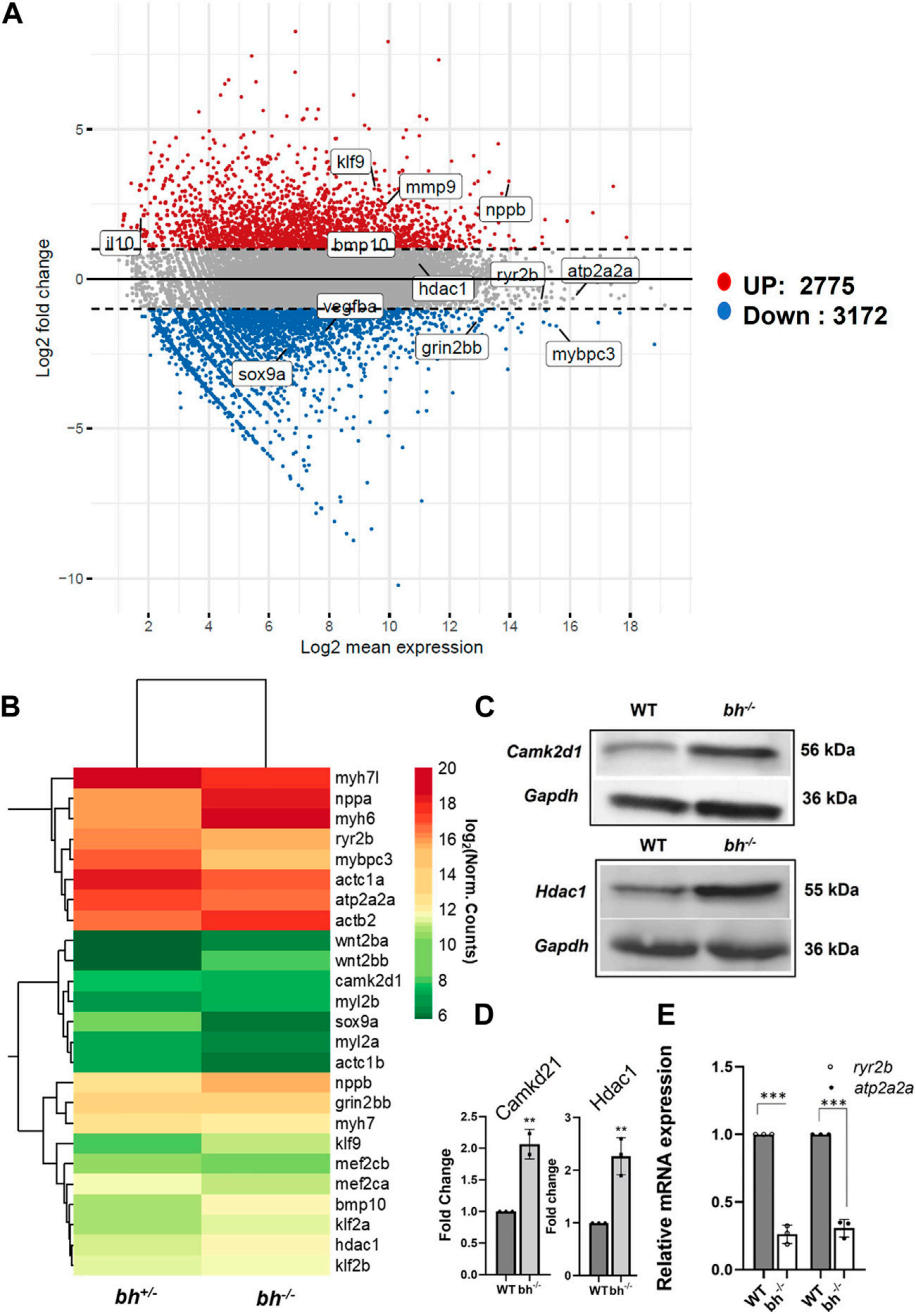
Transcriptome analysis of bigheart mutant. **(A)** MA-plot for differential expression analysis in RNA-seq of *bh* ^*+/−*^ and *bh* ^*−/−*^, x axis indicates the normalized mean expression (Log2 mean expression) and the y axis indicates the log 2 fold change (Red dots: upregulated genes and the blue dots: downregulated genes) (Log_2_ normal count). **(B)** Heat map showing differential expression of selected cardiac genes in *bh* ^*+/−*^ and *bh* ^*−/−*^ heart tissue (Log_2_ normal count). **(C)** Western blot analysis of calcium handling gene, Camk2d1 and cardiac remodeling gene, Hdac1 in adult heart tissue. **(D)** Quantification of Western blot in **(C)**. **(E)** mRNA expression of calcium handling genes ryr2b and atp2a2a in *bh* ^*+/−*^ and *bh* ^*−/−*^ heart. RT PCR analyses were performed three times in triplicates. **p < 0.01 and ***p < 0.001. The bars indicate the average values ± SD.

We used RNA sequencing to explore the effects of grin2bbART downregulation on the cardiac transcriptome in the bigheart heterozygous (*bh* ^*+/−*^) and homozygous (*bh* ^*−/−*^) mutant fish. The sequencing reads from the *bh* ^*+/−*^ and *bh* ^*−/−*^ mutant heart tissue were subjected to the transcript quantification (*N* = 57193) with Salmon tool using Ensembl gene annotations (Danio_rerio.GRCz11.111.gtf). We identified 5,947 genes (Figure 9A) including 76 well characterized cardiac genes (Supplementary Figure S12) that showed a 2-fold differential expression and normalized counts >0 in both samples across *bh* ^*+/−*^ and *bh* ^*−/−*^ mutant heart tissues. Overall, 3,172 genes were downregulated, and 2,775 were upregulated in *bh* ^*−/−*^ compared with *bh* ^*+/−*^ (Figure 9A). GO and KEGG pathway analysis revealed that major genes involved in calcium homeostasis were differentially expressed in the putative mutant heart tissues (Supplementary Figures S14, 15; Supplementary Tables S4–S7). Western blot analysis of Camk2d1 and other genes that are implicated in inducing hypertrophy, such as Hdac1, was performed on the protein lysates obtained from WT and *bh* ^*−/−*^ heart tissues. Camk2d1 protein levels were found to be elevated two-fold in the *bh* ^*−/−*^ mutant heart (*p* < 0.01) (Figures 9C, D). Similarly, Hdac1 levels were detected to be two-fold higher in the *bh* ^*−/−*^ mutant heart (*p* < 0.01) (Figures 9C, D). We validated the RNA sequencing-derived expression status of well-characterized cardiac marker genes (Figure 9B; Supplementary Figure S12) using qRT-PCR. We noticed the transcript levels of the key marker genes involved in Ca^2+^ homeostasis such as *ryanodine receptor* (*ryr2b*) and *atp2a2a* (*serca2a*) to be 8 fold diminished in the *bh* ^*−/−*^ mutant heart as compared to wild type (*p* < 0.001) (Figure 9B). In addition, we successfully validated the RNA-seq based gene expression findings using qRT-PCR for about twenty-four (24) cardiac specific gene transcripts (Figure 9E; Supplementary Figure S16). We expected that intracellular Ca^2+^ disturbances, as seen in the *bh* ^*−/−*^ mutant, would finally be manifested as altered protein expression of calcium handling genes. Further, elevated myocardial Matrix metalloproteinase-9 (MMP-9), a zinc-dependent endopeptidase that governs pathological cardiac remodeling processes like fibrosis and inflammation, is connected to ventricular arrhythmia (Weng et al., 2016). Our cardiac transcriptom in bigheart displayed an increased mmp9 expression by 2.5-fold when compared to the age matched heterozygotes siblings (Supplementary Figure S13B). This results suggest that mmp9 may be involved with bigheart phenotype.

### *Bigheart* transcriptome displays cardiac stress signatures

In addition to uncovering aberrations in calcium-handling genes, RNA seq analysis of cardiac tissue in the big heart mutant revealed altered expressions of cardiac remodeling genes (Figures 9A–C; Supplementary Figures S12, S13). Many cardiac remodeling genes such as *nppa*, *nppb, myh6, bmp10* exhibited increased transcript levels. ANF and BNP are important biomarkers in clinical cardiology (Levin et al., 1998; Volpe et al., 2014; Kerkelä et al., 2015). Two paralogous genes, nppa and nppb, encode natriuretic peptide hormones. The heart muscle largely expresses both genes during embryonic and fetal phases, although nppa expression is dramatically reduced in the ventricles after birth. The ventricular myocardium substantially upregulates nppa and nppb under cardiac stress. Additionally, natriuretic peptides are involved in cardiac hypertrophy, fibrosis, angiogenesis, cardiomyocyte proliferation, and viability (Man et al., 2018). *The atria and ventricles express nppa and nppb during development* (Zeller et al., 1987; Chien et al., 1991; Cameron et al., 1996), *Nppa marks differentiating myocardium and is a sensitive marker for congenital heart malformations* (Christoffels et al., 2000; Bruneau, 2011). Both genes are expressed in the heart after birth, although ventricular nppa expression is significantly downregulated (Bloch et al., 1986; Cameron et al., 1996). During hypertrophy and heart failure, nppa and nppb are reactivated in ventricular cardiomyocytes (Chien et al., 1991; Goetze et al., 2020; Man et al., 2021). Other key remodeling genes that displayed about a two-fold increase in expression include *mef2c* and *hdac1*. A group of remodeling genes that displayed decreased expression include *myh7, mybpc3, vmhc* and *vmhcl*. Sarcomeric genes exhibiting decreased expression were *mylz3, myl6, myh, actb2, actc1a, actc1b, and troponin (ttna, ttnc).* Further analysis showed that, the bigheart mutant displayed differential expression of cardiac-specific genes (Supplementary Figures S12, S13). Further RNA sequencing analysis shows altered expression of essential transcription factors such *tbx2a, tbx2b,* and upregulation of *klf9* (Figure 9B; Supplementary Figure S12). We found considerable downregulation of *tbx2a* and *tbx2b* in *bh* ^*−/−*^. *Tbx2* inhibits cardiac differentiation to create the AVC (Sedletcaia and Evans, 2011). Both *tbx2* genes in the zebrafish genome are needed to produce the AVC. Chamber abnormalities caused by the depletion of both gene products result in an enlarged atrium and a smaller ventricle, which decreases ventricular cardiomyocyte proliferation (Sedletcaia and Evans, 2011). The phenotype matches cardiac growth factor expression alterations. The Krüppel-like factor 9 (KLF9) is a transcriptional factor which regulate oxidative stress (Zucker et al., 2014). A recent study shows that *Klf9* was induced in ischemic cardiomyocytes and promotes the injury by elevating reactive oxygen species in the cardiomyocytes (Yan et al., 2019).

### Bigheart transcriptome shows upregulated inflammation and proliferation markers

Increasing evidence suggests that inflammation is crucial to many cardiovascular disorders, including cardiomyopathies. Indeed, inflammation can activate molecular pathways that cause cardiomyocyte enlargement, extracellular matrix buildup, and microvascular dysfunction (Lillo et al., 2023). Bigheart transcriptome shows overexpression of various cytokines, including *il1β* (2.83), *il4* (1.55), *il6* (2.43), *il10* (2.14), *il11a* (4.72), and chemokines (*ccl19*, *cxcl18b* (2.05), *cxcl8a* (2.93), chemokine receptors, *cxcr3* (4.13), *cxcr4* (2.32), *cxcr1* (1.18) (Supplementary Figure S12B; Supplementary Tables S5, S6). The bigheart transcriptom also displayed increased expression of cell cycle genes such as, *pcna* and *mki67* indicative of a proliferative state (Supplementary Figure S13B).

## Discussion

The importance of lncRNAs in cardiovascular disease is rising (Ounzain et al., 2015). Multiple studies have identified mammalian lncRNA repertoire associated with several cardiac pathophysiologies such as hypertrophy (Han et al., 2014; Viereck et al., 2016; Wang et al., 2016), ventricular septal defects (Song et al., 2013), myocardial infarction (Ounzain et al., 2015; Li et al., 2019), cardiac aging (Wang et al., 2015), and coronary heart disease (Broadbent et al., 2008; Rahimi et al., 2018; Lin and Bao, 2021). LncRNAs regulate epigenetic control of cardiac organogenesis and show discrete differences in expression between embryonic, developing, and adult hearts (Dorn and Matkovich, 2015). Several lncRNA transcripts with cardiac-specific roles have been identified, such as Kcnq1ot1 in ion channel activity (Korostowski et al., 2012), MIAT(myocardial infarction-associated transcript) in myocardial infarction (Ishii et al., 2006), ANRIL in atherosclerosis (Holdt et al., 2010), Carl (cardiac apoptosis-related lncRNA) in regulating cell death in cardiomyocytes (Wang et al., 2014b), DMK1 in myotonic muscular dystrophy (Mahadevan et al., 2006; Yadava et al., 2008) and Pvt1 in hypertrophy (Yu et al., 2015). Interestingly, the exact mechanism of action of these lncRNAs in the respective conditions is not entirely resolved.

This study reveals that transcript emanating as antisense to *grin2bb*, which we call *grin2bbART,* is also crucial for cardiac development through calcium homeostasis. While defining *grin2bbART* as an anti-sense non-coding transcript and how it regulates cardiac development requires further study. Collective data from this study and published data on RNA sequencing (Singh et al., 2016) confirm transcriptional activity at the insertion site at the *grin2bb* locus. *Grin2bbART* fulfills the criteria for a prototypical lncRNA transcript. Reduced levels of *Grin2bbART* expression and *Cre*-mediated restoration of *grin2bbART* transcript level in *bigheart* further support the link between the *grin2bbART* disruption and *bigheart* mutation. Downregulation of *grin2bbART* is directly related to the *bigheart* phenotype as *grin2bbART* MO injections in wild-type embryos produced a cardiac phenotype similar to that observed in the *bigheart* mutant. Though we have shown that grin2bb mRNA (qRT-PCR) and proteins do not show significant changes, it is difficult to rule out the possibility that functional changes in the Grin2bb protein occurred during the general event. Therefore, future studies should focus on the interplay of the Grin2bb protein coding gene and the associated RNA transcript (grin2bbART).

*Grin2bbART* is expressed in the zebrafish heart and is enriched explicitly in the atrium and *bulbus arteriosus in the adult*. Our group’s previous study has described well-represented gene expression diversity between the three cardiac chambers (atrium, ventricle, and bulbus arteriosus). We showed that the number of genes expressed in the atrium (5,951) and that in bulbus arteriosus (5,823) was equivalent; however, the ventricle expressed a somewhat larger repertoire (6,359). We observed that a subset of genes overlay between two chambers (Singh et al., 2016). We conjecture that this subset of genes predominantly has similar or complementary functions across more than one chamber. We found that the zebrafish atrium displayed a representation of genes involved in lipid transport, muscle contraction, and calcium ion transport. The ventricle showed enriched expression in a broader spectrum of genes, including transport proteins, receptor molecules, kinases and heat shock proteins apart from contractile proteins. The *bulbus arteriosus* majorly displayed contractile genes and calcium-binding proteins. It occurs that specific enrichment of these protein-coding genes in a particular chamber and differences in gene expression govern the key functions among different cardiac chambers (Singh et al., 2016). Thus, we believe that *grin2bbART* has a chamber specific role as noted by its expression being confined to the atrium and bulbus arteriosus.

The *bigheart* mutant displays typical arrhythmia and chamber enlargement in the atria and bulbus arteriosus. Atrium enlargement in *bigheart* is unique and different from other existing hypertrophy models. A prolonged R-R interval in the *bigheart* putative mutant signifies bradycardia, and a negative S wave indicates hypertrophic cardiac tissue. SEM analysis of the cardiac ventricle suggests disorganization of myofibrils and hypertrophy in the *bh* ^*−/−*^ fish. Staining of the heart section with (phalloidin a f-actin, a Z disc sarcomeric protein confirms enlarged atrium and the muscular disarray and hypertrophy in the ventricle. The *bh* ^*−/−*^ embryos had normal touch stimuli response, indicating unaffected neuromuscular networks. The above data suggest that *bigheart* mutation recapitulates pathological atrial dilation, ventricular hypertrophy, and arrhythmia characteristics in mammals (Makhro et al., 2016).

Notably grin2bbART downregulation in bigheart results in aberrations in cardiac Ca^2+^ activity as assessed by calcium green dextran assay. The significant decrease in the transcript levels of *ryr2* and *atp2a2a* (*serca*) in *bigheart*, suggests either disruption or alteration of intracellular Ca^2+^ homeostasis. Decreased expression of *serca* has been documented in heart failure and hypertrophy (Mercadier et al., 1990; Arai et al., 1993). Exposure of zebrafish to phenanthrene results in calcium handling defects leading to arrhythmia via *serca* downregulation (Zhang et al., 2013). Calcium expulsion perturbations in zebrafish *tremblor* mutants have been associated with atrial chamber arrhythmia (Ebert et al., 2005). A previous study reported that lncRNAs (MLN micropeptide) regulate and impinge upon SERCA function to block Ca^2+^ uptake into the SR (Bazzini et al., 2014). The reduced levels of a*tp2a2a* (*serca*) in the *bh* ^*−/−*^ fish present a possibility of *atp2a2a* being regulated by the *grin2bbART* function. In a scenario when *grin2bbART* is downregulated, an altered function of *atp2a2a* may result in aberrant calcium activity and lead to Ca^2+-^ mediated cardiac arrhythmia in *bigheart* fish. However, the possibility of *ryr2* contributing to defects seen in *bigheart* is minimal as zebrafish *ryr2* has a low sensitivity to CICR (Bovo et al., 2013).

Intracellular calcium influx is the prime requirement for *Camk2d* activation, altered calcium activity in the *bh* ^*−/−*^ mutant leads to Camk2d over-expression. An increased expression of Camk2d has previously been associated with ventricular hypertrophy in humans (Hoch et al., 1999; Zhang et al., 2020) and cardiomyopathy in mice (Zhang et al., 2003a; Zhang et al., 2005). Thus, the increase in *camk2d* transcript levels and its protein is consistent with the hypertrophy observed in *bh* ^*−/−*^ fish. Hdac1 is activated via phosphorylation by Camk2d (Zhang et al., 2020). Thus, an increase in Camk2d levels in the *bh* ^*−/−*^ mutant leads to elevated levels of active Hdac1 protein. Several classes of HDACs have been associated with cardiac hypertrophy (Zhang et al., 2012), and Hdac inhibition has been shown to reverse hypertrophic conditions and arrhythmia (Liu et al., 2008; Morales et al., 2016). Further, *mef2* is a transcription factor that regulates average cardiomyocyte growth but, when activated by Camk2d, induces hypertrophic conditions (Kolodziejczyk et al., 1999; Passier et al., 2000). Thus, it is evident that upregulation of *camk2d-hdac1-mef2* pathway leads to chamber enlargement, and arrhythmia in *bigheart* and *grin2bbART* is associated with this pathway. Arrhythmia and the cardiac chamber enlargement in *bigheart* may be a result of altered calcium handling, possibly via the Camk2d-Hdac1 pathway.

Calcium homeostasis in the heart is tightly regulated by the action of multiple ion channels, signaling pathways, and proteins. Key calcium calcium-handling genes such as ryanodine receptor (ryr) and sarco-endoplasmic reticulum Ca-ATPase (serca) are crucial in maintaining calcium homeostasis in cardiac cells. Alteration in either of these genes has been reported to result in calcium dysregulation, leading to contractile dysfunction of cardiomyocytes (Eisner and Trafford, 2002; Ebert et al., 2005). Camk2d activation is previously known to promote phosphorylation-dependent deactivation of Ryr, thus resulting in Ca^2+^ aberrations (Maier, 2005; Ling et al., 2009). Camk2d promotes hdac1 action via phosphorylation (Anderson, 2009; Zhang et al., 2020). Both Camk2d and Hdac1 are previously reported to regulate cardiac hypertrophy (Zhang et al., 2013; Morales et al., 2016; Zhang et al., 2020). Upregulation of MMP9 in Cardiac arrhythmi and hypertrophy is known (Weng et al., 2016; Li et al., 2019). In mice, myocardial MMP-9 inhibition modulates calcium homeostasis and reduces calcium leakage to prevent ventricular arrhythmia (Weng et al., 2016) and its overexpression mediates gap junction (GJ) electrical uncoupling induced by Cx43 decreases in rat (Li et al., 2019). Our data leads us to hypothesize that disruption of grin2bbART in *bigheart* mutant results in calcium mishandling in cardiomyocytes. Altered Ca^2+^ levels upregulate Camk2d, thus driving an augmented ryr deactivation and promoting Hdac1 action. Increased activity of the Hdac1 protein initiates transcription of downstream hypertrophic factors such as mef2 in the nucleus, which drives a gene expression cascade leading to arrhythmia and hypertrophy in the *bigheart* mutant. Further, given the well-studied Ca^2+^ permeable function of *grin2bb* gene, it would be reasonable to hypothesize that grin*2bb* and *grin2bbART* might show a potential co-regulation to influence intracellular calcium homeostasis in cardiomyocytes. However, this model requires further experimental evaluation.

Differential expression of cardiac specific genes, including the remodeling genes such as *nppa, nppb, myh6* in the *bigheart* cardiac transcriptome, indicates the onset of a fetal gene program to induce an adaptive physiological response to cope with the perturbed cardiac function. Hypertrophic adaptive response due to mutations or alterations in the expression of key sarcomeric proteins such as *myh7, mybpc3, tnnt2, tpm1, myl2,* and *acta* have been characterized (Tardiff, 2005; Caleshu et al., 2011). *Mybpc3* downregulation has previously been modeled in zebrafish to recapitulate human hypertrophy phenotypes (Chen et al., 2013). *Mybpc3* mutations are associated with familial hypertrophic cardiomyopathy in humans (Carrier et al., 1997; Carrier et al., 2015), fibrosis in pigs (Zou et al., 2022), and hypertrophy in mice (Schlossarek et al., 2012; Judge et al., 2015). *Myl2* is a sarcomeric gene involved in calcium-dependent cardiac muscle contraction (Sheikh et al., 2015). Notably, the downregulation of grin2bbART in the *bigheart* fish led to the abnormal expression of the cardiac remodeling genes and critical transcription factors, including *tbx2a, tbx2b,* and *klf2, klf5,* and *klf9,* and resulted in hypertrophy. Increasing evidence suggests that inflammation is crucial to many cardiovascular disorders, including cardiomyopathies. Indeed, inflammation can activate molecular pathways that cause cardiomyocyte enlargement, extracellular matrix buildup, and microvascular dysfunction. Growing data suggests that systemic inflammation may be a critical pathophysiologic factor in cardiac disease progression, affecting phenotypic severity and clinical prognosis, including heart failure (Lillo et al., 2023). Studies indicate elevated levels of cytokines like TNF-α, hs-CRP, and inflammatory interleukins (e.g., IL-1β, IL-1RA, IL-6, IL-10, circulating monocyte chemoattractant protein resulted in a chronic low-grade inflammatory state in hypertrophic cardiomyopathy (HCM) (Kuusisto et al., 2012). In line with these, our data also suggest a possible role of inflammation in the bigheart phenotype. Inflammatory cytokines may also be involved in arrhythmogenic cardiomyopathy and other disorders (Lazzerini et al., 2017; Lazzerini et al., 2022). It is well recognized that inflammatory cytokines, particularly TNF, IL-1, and IL-6, cause arrhythmias through direct cardiac activity and indirect systemic alterations. (Lazzerini et al., 2017). Direct effects include heart structural and electrical remodeling. Myofibroblast-driven extracellular matrix production by cytokines can cause structural remodeling over weeks or months (Lazzerini et al., 2017). These processes lengthen action potential duration or corrected QT (QTc) interval, increase ectopic firing, and delay or heterogeneously propagate electric impulses in the working and conducting myocardium. This causes triggered, re-entry-driven tachyarrhythmias, bradyarrhythmias, and conduction abnormalities (Lazzerini et al., 2017). Despite extensive research on the involvement of ncRNAs in cardiovascular diseases (Quiat and Olson, 2013; Bang et al., 2014; Amaral et al., 2013; Hermans-Beijnsberger et al., 2018), our understanding of the expression and function of lncRNAs in heart development remains limited (Devaux et al., 2015). Examining the expression patterns of grin2bbART at the single-cell level can reveal novel biological functions associated with various cell populations. Therefore, accurately quantifying the levels of grin2bbART in individual cells is crucial for identifying cell-type specificities and determining their functions at the single-cell level, as recounted by Chen et al. (2013) (Chen et al., 2019). Exploring the other affected genes will also unveil new *bh* ^*−/−*^ partners. Deciphering precise lncRNA function is a constant struggle, and an obvious conjecture that tethers a lncRNA to a function is the regulation of its neighboring protein-coding genes (Dinger et al., 2009; Guil and Esteller, 2012). Given the genomic association of *grin2bbART*, it is appropriate to propose that *grin2bbART* might be involved in the post-transcriptional and translational processes associated with the zebrafish *grin2bb* (*NMDAR2B*) gene. The role of NMDAR has been previously implicated in brain function, but its function in the heart remains elusive. Recent findings reported the expression of *NMDAR2B* in the rat heart (Seeber et al., 2004; Makhro et al., 2016) and in regulating the heartbeat (Makhro et al., 2016). Since NMDARs are permeable to Ca^2+^, it is reasonable to assume that NMDARs may have a key role in heart function. NMDAR-mediated Ca^2+^ influx worsens myocardial damage by ischemia and reperfusion-mediated necrosis and apoptosis (Liu et al., 2017). A recent study has suggested cardiac NMDARs may be a therapeutic focus in ischemia and reperfusion injury (Govoruskina et al., 2020). In zebrafish, *NMDAR2B* paralog (*grin2bb*) is reported to be expressed at low levels in the heart (Cox et al., 2005). Although evidence of NMDAR function in the heart, its exact role and mechanism of action remains obscure in zebrafish. We demonstrate that *grin2bbART*, a putative RNA transcript, is transcribed from the intron of *NMDAR2B* (*grin2bb*), and is involved in calcium mishandling, which led to hypertrophic response and arrhythmia in zebrafish heart. LncRNAs acting as regulators of hypertrophy, such as Chrf (cardiac hypertrophy-related factor) (Wang et al., 2014a; Han et al., 2014) and Pvt1 (Yu et al., 2015) have been recently discovered. The deep sequencing approach has uncovered several lncRNA linked to cardiac diseases (Yang et al., 2014). We used this approach to identify and annotate *grin2bbART*, a putative RNA transcript that is expressed in the zebrafish heart. We hypothesize that *grin2bbART* has a potential role in zebrafish heart function via regulating calcium flux possibly generated by the zebrafish *grin2bb* (*NMDAR2B*) gene. CICR is primarily believed to be dependent upon *ryr* or IP3 receptors. However, deviations from this have been reported wherein other factors or proteins contribute to CICR. The sarcolemma calcium entry and process of CICR to induce contraction is ambiguous and shows species-specific differences among teleost hearts. This study uncovers the possibility that *grin2bbART-grin2bb* interactions may be another undiscovered mode of CICR in zebrafish cardiomyocytes and presents a template to dissect these mechanisms in detail.

## Materials and methods

### Ethics statement

All the fish experiments were performed following the guidelines and the recommendations laid down by the CSIR- Institute of Genomics and Integrative Biology, India (CSIR-IGIB). The Institutional Animal Ethics Committee of CSIR-IGIB approved all protocols used in the study. Utmost care was taken to distress the animals minimally.

### GBT vector microinjection, gene trapping, and screening of pGBT-PX lines

Zebrafish were housed at the CSIR-Institute of Genomics and Integrative Biology, following standard husbandry practices (Westerfield, 2000). The pGBT-PX vector (Genbank accession No. HQ335166; Supplementary Figure S1) described in the present study was generated by subcloning the pT2/PAT6 GBT (Sivasubbu et al., 2006) into a mini tol2 vector (Balciunas et al., 2006). Tol2 transposase mRNA was synthesized *in vitro* using the Ambion mMESSAGE mMACHINE T3 Kit (Thermo Fisher Scientific, Texas, United States; AM1348), from the construct pDB600 (Balciunas et al., 2006). pDB600 was cleaved with *XbaI* and transcribed with T3 polymerase to produce Tol2 mRNA.

Fertilized zebrafish eggs at one cell stage were co-injected with 8.3 pg/nL of plasmid DNA and 25 pg of tol2 transposase mRNA as standardized previously (Hyatt and Ekker, 1999) Injected founder (F_0_) embryos were scored for green fluorescent protein (GFP) reporter expression at 24 h post fertilization (hpf), and only the GFP expressing embryos were raised to adulthood. F_0_ founder fishes were crossed to wild-type zebrafish to test the germline transmission rates by observing GFP reporter expression in F_1_ embryos from 1 day post fertilization (dpf) to 5 dpf (Supplementary Figure S2). As per the vector design, a consistent GFP expression is visible only when the trap vector gets inserted into an endogenous transcriptional unit and the GFP transcript is stabilized due to poly-adenylation by 3′ sequences of the endogenous gene or transcript. Adult GFP expressing F_1_ pGBT-PX heterozygous fish were incrossed to generate F_2_ embryos. The embryos were observed carefully from 1dpf to 5 dpf for phenotypes using a simple visual screen to score for any gross abnormalities. GFP-expressing F_2_ embryos were observed for phenotypic changes in the heart, blood circulation, body axis length, pigmentation, craniofacial morphology, brain size and shape, eye size, swim bladder, and motility. The observed phenotypes, if any, were recorded.

### Microscopy

To inhibit pigment formation, embryos were treated with phenyl thiourea (PTU) (0.003%). Embryos were anesthetized using 4 mg/mL Tricaine methanesulfonate (Sigma, A5040) and mounted in 0.8%–1% low melting agarose gel (Biorad, 161-3111) in embryo water for imaging. GFP expression was observed, and fish were imaged using an upright Zeiss Axioscope 40 fluorescent microscope (Carl Zeiss, Germany). Image processing was done using Zeiss AxioVision 4.6 and Adobe Photoshop CS software.

### Electrocardiogram recordings

Electrocardiogram (ECG) was recorded for wild-type (*bh* ^*+/+*^) and mutant (*bh* ^*−/−*^) zebrafish aged 9 months (Milan et al., 2006). ECG recordings were obtained by inserting two needle electrodes (AD Instruments, Animal Bioamp) through the ventral epidermis. Fish were perfused orally to support continuous hydration and oxygenation. Motion artifacts were eliminated with a paralytic dose of tuberculin (Life technologies). The use of a perfusion system facilitated stable recording for >6 h. Data analysis was done using AD Instrument LabChart software. *N* = 10 fish were analyzed in each group.

### Heartbeat rate measurement

Embryos at 2 dpf to 8 dpf were anesthetized using 4 mg/mL Tricaine methanesulfonate (Sigma, A5040). Video micrographs of heart rate in embryos were recorded by observation under a dissecting microscope (Zeiss). The number of beats per minute was counted for an interval of one minute using a Sony video camcorder for each individual fish. *N* = 10 embryos were analyzed per group.

### Hematoxylin-eosin and phalloidin staining

Hearts dissected from 9 months old wild type (*bh* ^*+/+*^) and mutant (*bh* ^*−/−*^) were fixed in 4% paraformaldehyde, embedded in paraffin, and cross sections were obtained. The sections were stained with hematoxylin and eosin (H&E) (Fisher Scientific) using a standard protocol. Heart tissue sections were observed under an inverted light microscope (Zeiss Axioscope 40) at a magnification of ×5, and details were documented using ×40 magnification. The cytoskeletal structure of heart tissue was observed using Invitrogen™ Texas Red™-X Phalloidin (Thermofisher Scientific), a stain that specifically stains F-actin filaments. Hearts were dissected from nine-month-old adult wild type and *bh* ^*−/−*^ mutant zebrafish as described previously (Singh et al., 2016) and fixed in 4% paraformaldehyde for overnight at 4°C. The heart tissue was processed as per the following protocol: 1X PBS wash for 1 min × 3 times; 0.1% TBST for 5 min × 3 times; 1X PBS wash for 5 min × 2 times. The tissues were incubated with PBS with 1% BSA for 5 min -pre-staining. A total of 5 µL of 6.6 µM of phalloidin conjugated with texas red in methanol was added to 200 µL of detection solution. Tissues were incubated in the dark for 20 min and were washed with 1X PBS × 3 times for 5 min each. Stained tissues were observed using an inverted light microscope (Zeiss Axioscope 40).

### Inverse PCR (*i*PCR)

Inverse PCR was performed as described previously (Balciunas et al., 2006; Hermanson et al., 2004). DNA from caudal fins of adult fish or from whole single embryos was isolated in 400 µL of DNA extraction buffer (10 mM Tris pH 8.2,10 mM EDTA, 200 mM NaCl, 0.5% SDS) containing 100 mg/mL proteinase K for 10 h at 55°C. Proteinase K was inactivated by incubating the samples at 65°C for 10 min. DNA was purified with phenol-chloroform, and the pellet was obtained by isopropanol precipitation. DNA pellets obtained were washed with 70% ethanol and re-suspended in 30 µL of TE buffer. Following set of forward primers were designed against the GFP sequences of the trap vector: **GFP-RACE** (5″GAG​AGA​CCA​CAT​GGT​CCT​TCT​TG-3′) and **GFP-NEST** (5′-CAG​CTG​CTG​GGA​TTA​CAC​AT-3′). The reverse primers used were: **SBB_537** (5′ATC​ACC​TTC​ACC​CTC​TCC​ACT​GAC-3′) and **SSB_536** (5′AAC​AAG​AAT​TGG​GAC​AAC​TCC​AGT​G-3′). PCR products were separated on a 1.2% agarose gel. Individual fragments were sliced from the gel and purified using the QIA quick gel extraction kit (QIAGEN) and sequenced using GFP nest forward and reverse primers.

### 5′ RACE and 3′ RACE

5′ and 3′ RACE was performed using 5′ RACE System (Invitrogen, United States) and 3′RACE System (Invitrogen, United States) kits, following the manufacturer’s protocol. Unique bands from the nested PCR of 5′ and 3′ RACE was gel-extracted and cloned in pCR 2.1-TOPO vector (Invitrogen, United States), and Sanger sequencing was performed on it using M13 primers. Total RNA was isolated from 20 to 25, 3dpf zebrafish embryos using Trizol reagent (Invitrogen). First-strand cDNA was synthesized using 5 µg of total RNA in a 20 µL reaction using superscript reverse transcriptase (Invitrogen). 3′ RACE-PCR was performed in a 50 µL reaction using nested primers. The primary step of 3′ RACE- PCR was performed using GFP primers, **GFP-RACE** (5′-GAG​AGA​CCA​CAT​GGT​CCT​TCT​TG- 3′) and the universal adapter primer–**AP** (5′-GGC​CAC​GCG​TCG​ACT​AGT​ACT​TTT​TTT​TTT​TTT​TTT​T-3′) (Invitrogen) with 2 µL of the cDNA as template. The following PCR conditions were used: initial denaturation at 94°C for 3 min followed by 94°C for 1 min, 65°C for 2 min (with subtraction of 0.5°C/cycle), 72°C for 1 min (with an addition of 2 s/cycle) for 30 cycles followed by 94°C for 1 min, 52°C for 2 min, 72°C for 2 min for 10 cycles with a final extension at 72°C for 10 min. For the nested reaction, 2 µL of 3′ RACE-PCR product of the primary step was used as the template. The following primers were used–**GFP-NEST** (5′ CAG​CTG​GGA​TTA​CAC​AT-3′) and abridged universal adapter primer (**AUAP**) 5′-GGC​CAC​GCG​TCG​ACT​AGT​AC-3′). The PCR conditions were as follows: initial denaturation of 94°C for 3 min followed by 94°C for 1 min, 60°C for 1 min, and 72°C for 1 min for 24 cycles followed by a final extension at 72°C for 10 min.

Trapped sequences obtained from iPCR and RACE were analyzed for identity using BLAT against the zebrafish reference genome (ZV9) and NCBI databases. The BLASTN was conducted against publicly available UCSC databases (http://genome.ucsc.edu/) and Ensembl (http://www.ensembl.rg/danio_rerio/). Genomic sequences bearing >90% identity to the trapped sequence were considered significant matches.

### Identification and annotation of long non-coding RNAs

We utilized the cardiac transcriptome data generated in our laboratory (Singh et al., 2016) to identify cardiac-specific long non-coding RNA (lncRNA) transcripts. We used a previously published computational analysis method for the identification of potential lncRNA transcripts (Kaushik et al., 2013). Transcripts of length more than 200 nt, lacking coding potential and with an ORF length of less than 30 amino acids (Kaushik et al., 2013), were considered as potential lncRNAs. In house Perl scripts were used to assess the length of the transcripts. Get ORF was utilized to acquire a length of ORFs, and coding potential was evaluated using a Coding potential calculator (CPC). CPC values for non-coding RNA transcripts were calculated. PhyloCSF and HMMER were employed to further confirm the coding potential of the transcripts. Transcripts that fulfilled all the above parameters for non-coding potential were considered as putative long non-coding RNAs.

### Ribosome profiling of *grin2bb* gene locus

Ribosome profiling data from NCBI SRA obtained from eight zebrafish developmental stages (2–4 cells, 256 cells, 1,000 cells, dome, shield, bud, 28 hpf and 5 dpf) (Chew et al., 2013). Adapters were trimmed (Ingolia et al., 2011) using FASTX clipper, a part of FASTX-Toolkit (http://hannonlab.cshl.edu/fastx_toolkit/). Reads mapping to rRNAs were removed after aligning the trimmed reads to zebrafish rRNA sequences downloaded from SILVA database (Quast et al., 2013) using Bowtie2 (Langmead and Salzberg, 2012). Finally, we isolated 235 million high-quality reads by placing a read length filter of 27–32 nt as described (Chew et al., 2013). These high-quality reads were then mapped using Tophat 2 (Kim et al., 2013) to the *de novo* transcriptome assembly. Further, we downloaded RNA-Seq reads for eight zebrafish developmental stages (2–4 cells, 1,000 cells, dome, shield, bud, 28 hpf, 2 dpf and 5 dpf) (Pauli et al., 2012) from NCBI SRA. After adapter and quality trimming using Trimmomatic (Bolger et al., 2014) and SolexaQA (Cox et al., 2010), the reads were mapped to the Zv9 reference genome using Tophat 2 (Kim et al., 2013). Translation Efficiency Score (TES) for the transcripts were calculated using BED Tools (Quinlan, 2014) by obtaining the Riboseq and RNA-seq read counts overlapping each transcript. We then formulated TES for each transcript as the ratio of its Ribo-seq read count to RNA-Seq mapped read count.

### qRT-PCR

Real-time quantitative PCR (qRT-PCR) was performed to analyze the transcript levels and the mRNA level of various genes including grin2bb and grin2bbART, in *bigheart* mutant embryos and morphants. 250 ng of total RNA in a 10 µL reaction using the Roche Diagnostics LightCycler 480 SYBR Green I Master (Roche Applied Sciences, Sigma) was amplified using the following primers: Forward primer (5′-ATC​AGG​AGG​CCA​TCG​CCC​AGA​TAT​TG-3′) and reverse primer (5′-GTG GTG ACG ATG GAG AAA ATG TACCA -3′) that were designed within the exons (Exon 2 and 3) flanking the insertion locus in the *grin2bb* gene. As a control, the zebrafish *β-actin* partial gene transcript was amplified using the following primers *β-actin* forward (5′-CTC​TTC​CAG​CCT​TCC​TTC​CT-3′) and *β-actin* reverse (5′CTT​CTG​CAT​ACG​GTC​AGC​AA- 3′). Reverse transcription was performed at 42°C for 30 min. Denaturation was performed at 95°C for 2 min, followed by 40 amplification cycles (Light cycler LC 480, Roche Diagnostics, Germany). The relative levels of specific transcripts in the original pool of RNA were estimated using the methods described (Winer et al., 1999; Livak and Schmittgen, 2001). The primers used for the *grin2bbART* and other genes are mentioned in Supplementary Table S2.

### Morpholino and mRNA injections

#### Antisense oligonucleotide morpholino injections

All antisense oligonucleotide morpholino sequences (MO) used in this study were obtained from Gene Tools, United States. MOs targeting translation and transcription were designed. The MO sequences used in the study are:

*grin2bbART* MO1*:* 5′- ACT​ACT​GAC​CTA​TAT​AAC​GAG​GTA​T- 3′;

*grin2bbART MO*2: 5′- AAT​GCA​GCC​ATA​CAG​TGT​ACG​TAC​T- 3′;

*grin2bbATG* MO: 5′- AAC​ATT​GCC​AGC​CCA​ACT​CCC​ATT​G- 3′;

*grin2bb* Splice MO1: 5′- ATC​ATA​CCT​TGG​CAG​CCA​TGA​TCA- 3′ and

*grin2bb* Splice MO2: 5′- GAA​CAT​GGA​ATG​GTC​ATC​CTG​CAA​G- 3′.

One-cell stage wild-type zebrafish embryos were injected in two doses: 3 nL and 6 nL of 100 µM solution of the MO. The injected embryos were evaluated for specific phenotypes.

#### Cre recombinase mRNA injections

50 pg of *Cre recombinase* mRNA was injected into one-cell stage embryos resulting from the pair wise breeding of *bh* ^*+/−*^ heterozygous fish for excision of the gene trap. The injected embryos were scored for the *Cre recombinase-*mediated reversion of the *bigheart* phenotype using PCR and phenotypic analysis.

### Calcium imaging

2 dpf zebrafish embryos obtained from pair-wise breeding of heterozygotes *bh* ^*+/−*^ fishes were anesthetized as described above and injected with 2 nL of a 250 μM stock of calcium green-1 dextran (Molecular Probes). Hearts of 2 dpf *bh* ^*−/−*^ mutants and their wild-type siblings injected with calcium green-1 dextran were imaged. High speed two-dimensional calcium images were captured at a rate of 30 Hz using a Zeiss laser scanning confocal imaging system using a ×20 objective lens at an excitation peak of 488 nm, and emission at 510 nm. Data was collected at a speed of 12 frames per sec. The relative fluorescence intensities (Y-axis) from consecutive frames of images (X-axis) were plotted in graphs where maximum fluorescence intensity of each data set was considered 100%. The fluorescence intensity (Calcium measurements) of individual hearts was analyzed by Zeiss confocal microscope ZEN2 software.

### Whole mount *in situ* hybridization (WISH)

Para-formaldehyde fixed embryos were processed for *in situ* hybridization according to standard zebrafish protocols (http://zfin.org/ZFIN/Methods/ThisseProtocol.html). The *grin2bb* gene sequences were amplified from cDNA by PCR using forward primer 5′-ATC​AGG​AGG​CCA​TCG​CCC​AGA​TAT​TG-3′ and reverse primer 5′-GTG​GTG​ACG​ATG​GAG​AAA​ATG​TAC​CA -3′ and cloned into Topo TA vector (Invitrogen, United States). The clone was linearized with *SpeI* (sense) and Not1 (antisense) restriction enzyme, and digoxigenin (DIG) labeled *in situ* probes was generated by *in vitro* transcription with T7 and T3 polymerases using the Roche’s DIG RNA Labeling kit (Millipore Sigma).

### Transcriptome analysis

#### RNA isolation and sequencing

Hearts were dissected out from the heterozygous (*bh* ^*+/−*^) and mutant (*bh* ^*−/−*^) adult (1 year old, male and female) zebrafish (*N* = 15) under a dissecting microscope (Zeiss Axioscope 40 microscope, Carl Zeiss, Germany). The heart tissue was carefully removed by inserting iris scissors and cutting around the interface. Utmost care was taken to prevent contamination and to obtain pure homogeneous samples. Dissected heart samples were rinsed with PBS and immediately flash-frozen in liquid nitrogen. Total RNA was isolated using a modified-step purification protocol by homogenization using a pellet pestles cordless motor (Z359971 SIGMA) in trizol (Invitrogen, United States) followed by purification using phenol-chloroform method as per standard protocol. RNA quality was assessed as previously described (Kaushik et al., 2013). RNA libraries were prepared and sequenced according to the manufacturer’s instructions for the TruSeq RNA sample prep Kit v2 (Illumina Inc., United States). The raw transcriptome sequences have been submitted to SRA with accession numbers SRX1158743: zebrafish Bigheart homozygous cardiac mutant (*bh* ^*−/−*^)*,* SRX1158742: zebrafish Bigheart heterozygous cardiac mutant (*bh* ^*+/−*^) transcriptome.

#### Transcript quantification and differential expression analysis

The RNA sequencing reads were used for transcript quantification by Salmon tool (Patro et al., 2017) using gene annotations from the Ensembl database (Danio_rerio.GRCz11.111.gtf). The R-package Tximport was used to convert the transcript quantifications to gene quantifications (Soneson et al., 2015). Differentially expressed genes were identified using R package DESeq2 (Love et al., 2014) with an absolute value of a log2(FC) > 1 and normalized counts >0 in both samples. Heatmaps were plotted using R package, pheatmap. Gene ontology and KEGG pathway enrichment analysis were performed using DAVID (Sherman et al., 2022) and visualized using GOplot R package (Walter et al., 2015). The relative expression of the key genes involved in cardiac function was assayed using qRT-PCR. A list of primers is given in Supplementary Table S2.

### Western blotting

Freshly isolated heart tissues (*N* = 10) from wildtype and *bh* ^*−/−*^ mutant fish were homogenized in 1X NP40 cell lysis buffer (Invitrogen, Thermo Scientific) supplemented with protease inhibitors. Homogenate was centrifuged at 14,000 g for 20 min. Protein concentration in the supernatant was estimated using the standard BCA method (Smith et al., 1987), and after that, the protein sample was processed for SDS-PAGE. ECL (Enhanced Chemiluminescence) reagent (Millipore) was used to evaluate the protein signal for NMDAR2B (Abcam, Anti-NMDAR2B antibody, ab65783), Camk2d [Abcam, Anti-CaMKII delta antibody (ab181052), and Hdac1 (Neo Bio Labs). Gapdh (Neo BioLabs) and Actin-beta (Cell Signaling Technology, β-Actin (8H10D10) Mouse mAb #3700] were used as loading controls for the experiment.

### Phalloidin staining

For phalloidin staining, the heart tissue was fixed in 4% paraformaldehyde overnight (4°C), followed by immersion in a 30% sucrose solution for one to 2 hours. The sections (5 µm) were cut using a cryotome. Rhodamine/Alexa-594 conjugated phalloidin (1:50; Molecular Probes) was used to stain the actin filaments.

### Scanning electron microscopy (SEM)

SEM analysis was performed in ventricle tissue isolated from the wildtype and the mutant (*bh* ^*−/−*^) zebrafish following the previous protocol (Hu et al., 2001; Sun et al., 2009) and imaged at the CSIR-IGIB, Electron Microscopy Core Facility.

### Statistics

Data are represented as mean ± SD of dependent samples. The comparisons were statistically tested by Student’s t-test and *Two way Annova* on Graphpad Prism 9. The *p*-values of <0.05 were considered significant. Number of times the experiment is performed is 3 or otherwise mentioned.

## Data Availability

The datasets presented in this study can be found in online repositories. The names of the repository/repositories and accession number(s) can be found in the article/Supplementary Material.
