# Supplementary material for: Forward genetic screen using a gene-breaking trap approach identifies a novel role of grin2bb-associated RNA transcript (grin2bbART) in zebrafish heart function
Source: Front Cell Dev Biol. 2024 Mar 8;12:1339292. doi: 10.3389/fcell.2024.1339292 (PMC10964321; doi:10.3389/fcell.2024.1339292)
Supplement: Supplementary file 1 [file DataSheet2.PDF]

## Cloning and generation of *myl7 t*ransgenic line

The 278 bp promoter region of myl7 was PCR amplified using zebrafish genomic DNA (Fig 1) using primer SSB_P1210 and SSB_P951 containing BglII cloning RE site (Huang et al., 2003). Subsequently, the PCR-amplified product was initially cloned into the TOPO-TA vector (TOPO™ TA Cloning™ Kit), ThermoFisher Scientific. The sequences of the cloned product were confirmed through Sanger sequencing. Finally, the verified myl7 TOPO-TA vector was used to clone the myl7 promoter sequence using BglII RE sites upstream of the RFP gene into a mini-Tol2 vector. To generate a stable myl7 transgenic line, the *myl7*:RFP Tol2 vector and Tol2 transposase mRNA, as described earlier, were injected into the one-cell stage of zebrafish. The embryos were raised to adulthood and crossed to wild type to identify the germline transgenesis.

**Forward Primer (SSB_P1210)**: AAAAGATCTGCGAATTCCGGCCGCTAAAT
**Reverse Primer (SSB_P951)**: AAAAGATCTGCAGGTTTAAACGAATTCGC
(BglII RE sites are underlined in the primers)

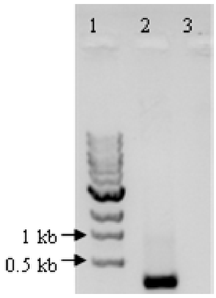


| Lane | Sample | Expected | Observed |
|---|---|---|---|
| 1 | 1 kb ladder | | |
| 2 | Cmlc2 | 278 | Below 0.5 kb |

**Fig1**.: Gel picture showing 278 bp PCR amplification in lane 2 of cmlc2 minimal promoter.

### Sequencing results for Cmlc2/pSS536

Following sequencing results showed that the 278 bp cmlc-2 promoter region was cloned in the TOPO-TA vector. The sequencing was done using universal T7 and T3 primers.

> LM304_T7-1.ab1

GAGCAATAGCGAGTGTAGTACTTGAGTAATTTTACTTGATTACTGTACTTAAGTATTATTTTTGGGGATTTTTACTTT
ACTTGAGTACAATTAAAAATCAATACTTTTACTTTTACTTAATTACATTTTTTTAGAAAAAAAAGTACTTTTTACTCC
TTACAATTTTATTTACAGTCAAAAAGTACTTATTTTTTGGAGATCACTTCATTCTATTTTCCCTTGCTATTACCAAAC
CAATTGAATTGCGCTGATGCCCAGTTTAATTTAAAT**AGATCT**GCAGGTTTAAACGAATTCGCCCTTCATCCCTCAAAT

CTCTCATTCACGTCCCCCTCCCCATCTGCACACTTTATCTCATTTTCCACCCTGCTGGAATCTGAGCACTTGTGCAGT
TATCAGGGCTCCTGTATTTAGGAGGCTCTGGGTGTCCATGTAGGGGACGAACAGAAACACTGCAGACCTTTATAGAAG
AACAAATGATAAGAGTCCTCATACATAAAGACTCCATTAGAAACGTCAGTGACCCAGGAGCCCAGACCAACAGCAAAG
CAGACAGTGAACATGGTGAGTAGACAAAGCAAGGGCGAATTCCGGCCGCTAAAT**AGATCT**GGCCA**TCTAGA**GCGGCCG
CGCGCACTAGTGAATTCCATGGCCAGCTCCGAGGATGTCATCAAAGAGTTTATGAGATTTAAGGTCAAGATGGAGGGA
AGCGTCAACGGACACGAGTTCGAGATTGAGGGAGAAGGAGAAGGCCGGCCTTACGAGGGCACACAAACCGCTAAGCTC
AAGGTCACAAAAGGAGGACCCCTCCCCTTCTCCTGGGATATTCTGAGCCCTCAGTTCCAGTACGGAAGCAAAGCCTAT
GTTAAACACCCTGCCGACATCCCTGACTATCTGAAGCTCTCCTTCCCTGAAGGCTTCAAGTGGGAGAGATTCATGAAC
TTCGAGGACGGAGGCGTGGTGACAGTCACACAAGATAGCACCCTCCAGGACGGAGAGTTTATTTATAAGTGAAACTCA
GAGGACCAACTTCCCCTCCGATGGCCCTGTCATGCAAAAAAACATGGGATGGCAGCTCCACCGAAAGATGTATCCTGA
AGATGCGCTCTGAAGGCGAATTAAATGAGACTGAAACTCAAGACGGAGGACTACCGATGCGAGTCAAACA

- LM304_p1195.ab1

GGGATTGTCGTTGAGCTTCCTCCATCTTGACCTTAAATCTCATAAACTCTTTGATGACATCCTCGGAGCTGGCCATGG
AATTCACTAGTGCGCGCGGCCGC**TCTAGA**TGGCC**AGATCT**ATTTAGCGGCCGGAATTCGCCCTTGCTTTGTCTACTCA
CCATGTTCACTGTCTGCTTTGCTGTTGGTCTGGGCTCCTGGGTCACTGACGTTTCTAATGGAGTCTTTATGTATGAGG
ACTCTTATCATTTGTTCTTCTATAAAGGTCTGCAGTGTTTCTGTTCGTCCCCTACATGGACACCCAGAGCCTCCTAAA
TACAGGAGCCCTGATAACTGCACAAGTGCTCAGATTCCAGCAGGGTGGAAAATGAGATAAAGTGTGCAGATGGGGAGG
GGGACGTGAATGAGAGATTTGAGGGATGAAGGGCGAATTCGTTTAAACCTGC**AGATCT**ATTTAAATTAAACTGGGCAT
CAGCGCAATTCAATTGGTTTGGTAATAGCAAGGGAAAATAGAATGAAGTGATCTCCAAAAAATAAGTACTTTTTGACT
GTAAATAAAATTGTAAGGAGTAAAAAGTACTTTTTTTTCTAAAAAAATGTAATTAAGTAAAAGTAAAAGTATTGATTT
TTAATTGTACTCAAGTAAAGTAAAAATCCCCAAAAATAATACTTAAGTACAGTAATCAAGTAAAATTACTCAAGTACT
TTACACCTCTGGGCCCAATTCGCCCTATAGTGAGTCGTATTACAATTCACTGGCCGTCGTTTTACAACGTCGTGACTG
GGAAAACCCTGGCGTTACCCAACTTAATCGCCTTGCAGCACATCCCCCTTTCGCCAGCTGGCGTAATAGCGAAGAAGG
CCCGCACCGATCGCCCTTCCCAACAGTTGCGCAGCCTTGAATGGCGAATGGACGCGCCCTGTAGCGGGCGCATTAAGC
GCGCGGGTGTGGTGGTTTACGCGCAGCGTGAACGCTACACTTTGCAGCGCCCTAGCGCCCGCTCATTCGCTTTCCTTC
CCTCCTTTCTCGCCACGTCGCAGGCTTCCCGGTCAGCTCTAATCGGGGCTCCCTTAGTCGATTATGCTACCGACCTTG
ACAAAAACCTGAATAAGGTTAATGGTCAGCAGGTGAGATG

Huang, C.J., Tu, C.T., Hsiao, C.D., Hsieh, F.J., and Tsai, H.J. (2003). Germ-line transmission of a myocardium-specific GFP transgene reveals critical regulatory elements in the cardiac myosin light chain 2 promoter of zebrafish. *Dev Dyn* 228(1)**,** 30-40. doi: 10.1002/dvdy.10356.
