# Supplementary material for: Forward genetic screen using a gene-breaking trap approach identifies a novel role of grin2bb-associated RNA transcript (grin2bbART) in zebrafish heart function
Source: Front Cell Dev Biol. 2024 Mar 8;12:1339292. doi: 10.3389/fcell.2024.1339292 (PMC10964321; doi:10.3389/fcell.2024.1339292)
Supplement: Supplementary file 5 [file Table1.pdf]

Table 1. Molecular loci trapped by the pGBT-PX insertion in the zebrafish genome

Sl no	Line name	Chromosome number	Integration location	Gene Symbol	Gene / Transcript name	Gene function	ENSDADRD ID	Human ortholog gene	Integration Mapping Method
**CLASS 1**									
1	GBT-PX-66F1	Chr 23	exon 1	edem2	ER degradation enhancer, mannosidase alpha-like 2	Calcium ion binding	ENSDART00000033774	EDEM2	NGS
2	GBT-PX-29F1	Chr24	exon 2	si:ch211-37e10.2	si:ch211-37e10.2	Calcium ion binding	ENSDART00000156419		NGS
3	GBT-PX-65F1 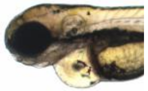	Chr3	exon 2	zgc:77407/helicase with zinc finger	helicase with zinc finger	Regulation of transcription	ENSDARG00000055089	HELZ	3'Race
4	GBT-PX-18F1	Chr8	exon	rho	rhodopsin	G -protein coupled receptor	ENSDARG0000002193	RHO	3'Race
**CLASS 2**									
5	GBT-PX-52F1	Chr16	intron 1	me1	malic enzyme 1, NADP(+)-dependent, cytosolic	Malate metabolic process	ENSDARG00000053215	ME1	NGS
6	GBT-PX-44F1	chr13	intron 1	slc24a3	solute carrier family 24 (sodium/potassium/calcium exchanger), member 3	transmembrane transport	ENSDART00000146227	SLC24A3	NGS
7	GBT-PX-22F1	Chr14	intron1	htra2	HtrA serine peptidase 2	Unannotated	ENSDART00000099626	HTRA2	NGS
8	GBT-PX-32F1	Chr 4	intron 1	nap1l1	nap1l1	Nucleosome assembly	ENSDART00000011882	NAP1L1	NGS/ iPCR/ 3'RACE

Sl no	Line name	Chromosome number	Integration location	Gene Symbol	Gene / Transcript name	Gene function	ENSDADRD ID	Human ortholog gene	Integration Mapping Method
9	GBT-PX-15F1	Chr 7	intron1	cyp2r1-003	cytochrome P450, family 2, subfamily R, polypeptide 1	Oxidation-reduction process	ENSDART00000149496	CYP2R1	NGS
10	GBT-PX-66F1	Chr15, Chr 13	intron 1	rxfp2a, atrnl1a	relaxin/insulin-like family peptide receptor 2a, attractin-like 1a	G-protein coupled receptor signaling pathway, Unannotated	ENSDARG00000019660, ENSDARG00000077188	RXFP2, ATRNL1	NGS
11	GBT-PX-69F1	Chr9	intron 2	myo16	myosine XVI	ATP binding	ENSDART00000109288	MYO16	NGS
12	GBT-PX10F1 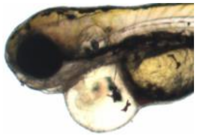	Chr 14, Chr 24	intron2, intron1,	sytl4, kcnb2	synaptotagmin-like 4, potassium voltage-gated channel, Shab-related subfamily, member 2	Exocytosis, Rab GTPase binding, Ion channel	ENSDARG00000102433 , ENSDARG00000088842	KCNB2	3'Race
13	GBT-PX-62F1	Chr 7, Chr 11	intron 2, intron 1	FRMD5, agpat2	Novel protein coding, intergenic, 1-acylglycerol-3-phosphate O-acyltransferase 2 (lysophosphatidic acid acyltransferase, beta)	Unannotated, Lipid metabolic process	ENSDART00000110409, ENSDART00000098983	AGPAT2	NGS
14	GBT-PX-64F1	Chr10, Chr14	intron 1, intron 3	FP236598.1, macrod1	FP236598.1, MACRO domain containing 1	Unannotated	ENSDARG00000089527, ENSDARG00000029609	MACROD1	NGS

Sl no	Line name	Chromosome number	Integration location	Gene Symbol	Gene / Transcript name	Gene function	ENSDADRD ID	Human ortholog gene	Integration Mapping Method
15	GBT-PX-49F1	Chr23	intron 10	cntn3a	contactin 3a, tandem duplicate 1	Unannotated	ENSDARG00000062880	CNTN3	NGS
16	GBT-PX-19F1	Chr22	intron 11	armc9	armadillo repeat containing 9		ENSDART00000125991	ARMC9	NGS
17	GBT-PX-51F1	Chr 16	intron11	fam188a, intergenic	family with sequence similarity 188, member A	Apoptotic process	ENSDARDRP00000059034	FAM188A	3' Race / NGS
18	GBT-PX-40F1	Chr 1	intron 12	nfixa	nuclear factor I/Xa	Skeletal muscle fiber development	ENSDARG00000043226	NFIX	NGS
19	GBT-PX-34F1	Chr20	intron 13	ino80	INO80 complex subunit,	ATP binding	ENSDART00000128806	INO80	NGS/ iPCR
20	GBT-PX-43F1	Chr 9, Chr 17,	intron 6, intron 1	mamdc1, ildr2	MAM domain containing 1, immunoglobulin-like domain containing receptor 2	Endoderm development	ENSDARG00000024017, ENSDARG00000014180	ILDR2	NGS
21	GBT-PX-37F1	Chr 17	intron 5	ttc27	tetratricopeptide repeat domain 27	Unannotated	ENSDARG00000007918	TTC27	NGS
22	GBT-PX-42F1	Chr15	intron 38	nbeab	neurobeachin b	Unannotated	ENSDARG00000010158	NBEA	NGS/ 3'RACE
23	GBT-PX-46F1	Chr7	intron 42	focad	focadhesin	Unannotated	ENSDART00000037846	FOCAD	NGS
24	GBT-PX-63F1	ZV9_NA895, Chr23, Chr 1,	intron 3, intron 6	spryd3, klf3	SPRY domain containing 3, Kruppel-like factor 3 (basic)	Unannotated, Erythrocyte maturation, chromatin binding	ENSDARG00000075799, ENSDARG00000015495	SPRYD3, KLF3	NGS

Sl no	Line name	Chromosome number	Integration location	Gene Symbol	Gene / Transcript name	Gene function	ENSDADRD ID	Human ortholog gene	Integration Mapping Method
25	GBT-PX-54F1	Chr 19, Chr 20, Chr1	intron 14, intron 1, intron 7	bai2, si:dkey-273g18.4, arfip1	adhesion G protein-coupled receptor B2, si:dkey-273g18.4, ADP-ribosylation factor interacting protein 1 (arfaptin 1)	G-protein coupled receptor signaling pathway, Regulation of protein secretion	ENSDARG00000025667, ENSDARG00000097778, ENSDARG00000070055	ADGRB2,ARFIP1	NGS
26	GBT-PX-65F1	Chr 13	intron 4	mrarch8	membrane-associated ring finger (C3HC4) 8	Protein ubiquitination, zinc ion binding	ENSDARG00000062489	MRACH8	NGS
27	GBT-PX-56F1	Chr 1, Chr 25, ZV9_NA503	intron1, intron 9	SGCZ, sergef	sarcoglycan, zeta, secretion regulating guanine nucleotide exchange factor	Unannotated, Unannotated	ENSDARG00000090659, ENSDARG00000070986		NGS
28	GBT-PX-53F1	Chr 8, Chr 4,	intron 3, intron 10	slc12a5b, si:dkey-264f17.2	solute carrier family 12 (potassium/chloride transporter), member 5b, si:dkey-264f17.2	Posterior lateral line neuromast development, Unannotated	ENSDARG00000078187, ENSDARG00000087499	SLC12A5	NGS

Sl no	Line name	Chromosome number	Integration location	Gene Symbol	Gene / Transcript name	Gene function	ENSDADRD ID	Human ortholog gene	Integration Mapping Method
29	GBT-PX-17F1	Chr5	intron 9	tmem161b	transmembrane protein 161B	Unannotated	ENSDARG00000055989	TMEM161B	NGS
30	GBT-PX-24F1 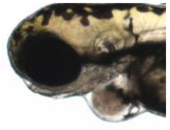	Chr1	intron 2	grin2bb-001	glutamate receptor, ionotropic, N-methyl D-aspartate 2B, genome duplicate b	Ion transport	ENSDARG00000030376	GRIN2B	NGS/ 3'RACE/ iPCR
31	GBT-PX-36F1	Chr15, Chr16,	intron 13, intron 2	dhx40, RFTN1	DEAH (Asp-Glu-Ala-His) box polypeptide 40, raftlin, lipid raft linker 1	RNA processing, and ATP binding , Unannotated	ENSDARG00000041586, ENSDARG00000086499	DHX40	NGS
32	GBT-PX-27F1	Chr16,	intron 12	cct3	chaperonin containing TCP1, subunit 3 (gamma)	Embryonic eye morphogenesis	ENSDARG00000016173	CCT3	NGS
33	GBT-PX-39F1	Chr21, Chr2, Chr14,	intron 1, intron 6, intron 3	chrdl2,hcn2, dmbx1a	chordin-like 2, hyperpolarization activated cyclic nucleotide gated potassium channel 2, diencephalon/mesencephalon homeobox 1a,	Dorsal / ventral pattern formation, Unannotated, Regulation of neurogenesis	ENSDARG00000078916, ENSDARG00000090115, ENSDARG00000009922	CHRDL2, HCN2, DMBX1	NGS
34	GBT-PX-25F1	Chr8	intron 15	itga8	integrin, alpha 8	Cell adhesion	ENSDARG00000078717	ITGA8	NGS

Sl no	Line name	Chromosome number	Integration location	Gene Symbol	Gene / Transcript name	Gene function	ENSDADRD ID	Human ortholog gene	Integration Mapping Method
**CLASS 3**									
35	GBT-PX-21F1	Chr13, Chr14, Chr20	intergenic						NGS
36	GBT-PX-16F1	Chr6	intergenic						NGS
37	GBT-PX-14F1	Chr21,	intergenic						NGS
38	GBT-PX-62F1	Chr 10	intergenic						NGS
39	GBT-PX-16F1	Chr15	intergenic						NGS
40	GBT-PX-22F1	Chr 22, Chr 19	intergenic						NGS
41	GBT-PX-12F1	Chr 7	intergenic						NGS
42	GBT-PX-18F1	Chr7	intergenic						NGS
43	GBT-PX-25F1	Chr14, Chr16, Chr10, Chr16	Intergenic						NGS
44	GBT-PX-23F1	Chr6	intergenic						NGS
45	GBT-PX-41F1	Chr5	intergenic						NGS
46	GBT-PX-58F1	Chr 6	intergenic						NGS
47	GBT-PX-22F1	Chr10, Chr 1, Chr17	intergenic						NGS
48	GBT-PX-39F1	Chr4, Chr6	intergenic						NGS
49	GBT-PX-27F1	Chr15	intergenic						NGS
50	GBT-PX-17F1	Chr25, Chr22	intergenic						NGS
