# Supplementary material for: Forward genetic screen using a gene-breaking trap approach identifies a novel role of grin2bb-associated RNA transcript (grin2bbART) in zebrafish heart function
Source: Front Cell Dev Biol. 2024 Mar 8;12:1339292. doi: 10.3389/fcell.2024.1339292 (PMC10964321; doi:10.3389/fcell.2024.1339292)
Supplement: Supplementary file 6 [file Table2.PDF]

Table 2. List of primers used in this study

S.No	Oligo Name	Forward Sequence	Reverse Sequence
1	acta1	TACTTCAGGACTCACGAGA	TCAGGATTTTACCTGGTGA
2	acta1b	ATGTGTGACGACGAGGAGAC	TGCTCTGAGCCTCATCACC
3	atp2a2a	ATCGGTAAGATCCGTGATGA	CACGGCGATCTTGAAGTAGT
4	b-actin	CTCTTCCAGCCTTCCTTCCT	ATCTTCATGGTGGAAGGAGCA
5	grin2bb ART	CCTTATGTGCCTTTACTCACTTAC	CTACAACAAATGATTGACTGAC
6	gsk3b	AGATGGCAGTAAAGTGACAC	GTAACGCAGACGAACAATGT
7	hand2	GTATTCAGGGCTGTACGAAGGT	TCGACCATTCGCACTACGGA
8	hdac1	GTCGGGAATTACTACTATGGTCAG	GATGGAGCGGAGGAACTTGAT
9	mhy6	TGAAGACCTGAGAAGGCAAC	CAGTTCCTCGGTTCTCTGAA
10	mybpc3	TCAACCAGGATGCCAGAGCCAACTA	CTTCAGGTGCGACTCCATCCGAT
11	myl2b	GCCGGCTGAACGTCAAACAGG	CTTCTCTCCAAACATGGTGAGGA
12	mylz3	ACTGCTGACCAGATTGAGGA	CAGGAAAGCCTCAAAGTCAA
13	nppa	AGCTTGCTGCAGCAGTTTGAG	GTGTGTCAAATCCATCCGAGG
14	ryr2b	CGAGACAAAGCTCAGGATGT	TTCGTGAGGGACTCTTTCAG
15	serca 2a	AGTCAATATGGGAGCTTGTCA	CTGTCAGAATAGACTGGTCCA
16	tnnt2a	ATGTCAGACAACGAAGAAGTGG	GAAGTGCAGACTGGTGAGCGTCT
17	tnnt1	AGTGAGCTTCGATCAGCGTCT	TCAGATGGCAGAGTTTGGAG
18	vmhc	GCTTCCTTTACAGTTACAGTCTTTC	GCGATGCTGAAATGTCTGTT
19	bmp4	AGGGAAGAAGAAAGCGTCGG	TAGTCGAAGCTGACGTGCTG
20	vmhcl	GCGATGCTGAAATGTCTGTT	CAGTCACAGTCTTGCCTCCT
