# Supplementary material for: Forward genetic screen using a gene-breaking trap approach identifies a novel role of grin2bb-associated RNA transcript (grin2bbART) in zebrafish heart function
Source: Front Cell Dev Biol. 2024 Mar 8;12:1339292. doi: 10.3389/fcell.2024.1339292 (PMC10964321; doi:10.3389/fcell.2024.1339292)
Supplement: Supplementary file 7 [file DataSheet3.PDF]

Forward genetic screen using a gene breaking trap approach identify a novel role of grin2bb associated RNA transcript (grin2bbART) in zebrafish heart function.

Authors: Ramcharan Singh Angom[1,4], Adita Joshi[1,], Ashok patowary[1,a], Ambily Sivadash[2,b], Soundhar Ramssay[1,c], Shamsudheen K V[1, 2 ,3], Kriti Kaushik[1, 3, d], Ankit Sabarwal[1,5, e], Mukesh Kumar Lalwani[1, f], Subburaj K[1], Naresh Singh[1], Vinod Scaria[1, 2, 3], and Sridhar Sivasubbu[1, 3*]

[1]Genomics and Molecular Medicine, CSIR Institute of Genomics and Integrative Biology, Mathura Road, Delhi 110025, India

[2]GN Ramachandran Knowledge Center for Genome Informatics, CSIR Institute of Genomics and Integrative Biology, Mathura Road, Delhi 110025, India

[3]Academy of Scientific and Innovative Research, Ghaziabad, India

[4]Department of Biochemistry and Molecular Biology, Mayo Clinic College of Medicine and Science, Jacksonville, FL 32224

Current Address:

[a] University of California, Los Angeles

[b] St Johns Research Institute, Bengaluru Area, India

[c] Weill Cornell Medicine, New York

[d] Department of Biochemistry, All India Institute of Medical Sciences, Delhi, India.

[e] Department of Pediatrics, Dell Medical School, University of Texas, Austin.

[f] Radcliffe Department of Medicine, University of Oxford

Address of Correspondence

Dr. Sridhar Sivasubbu, Ph.D

Senior Principal Scientist

CSIR Institute of Genomics and Integrative Biology

Sukhdev Vihar, Mathura Road,

New Delhi-110 025

Tel:91-11-29879106

e-mail: sridhar@gmail.com

Fig S1

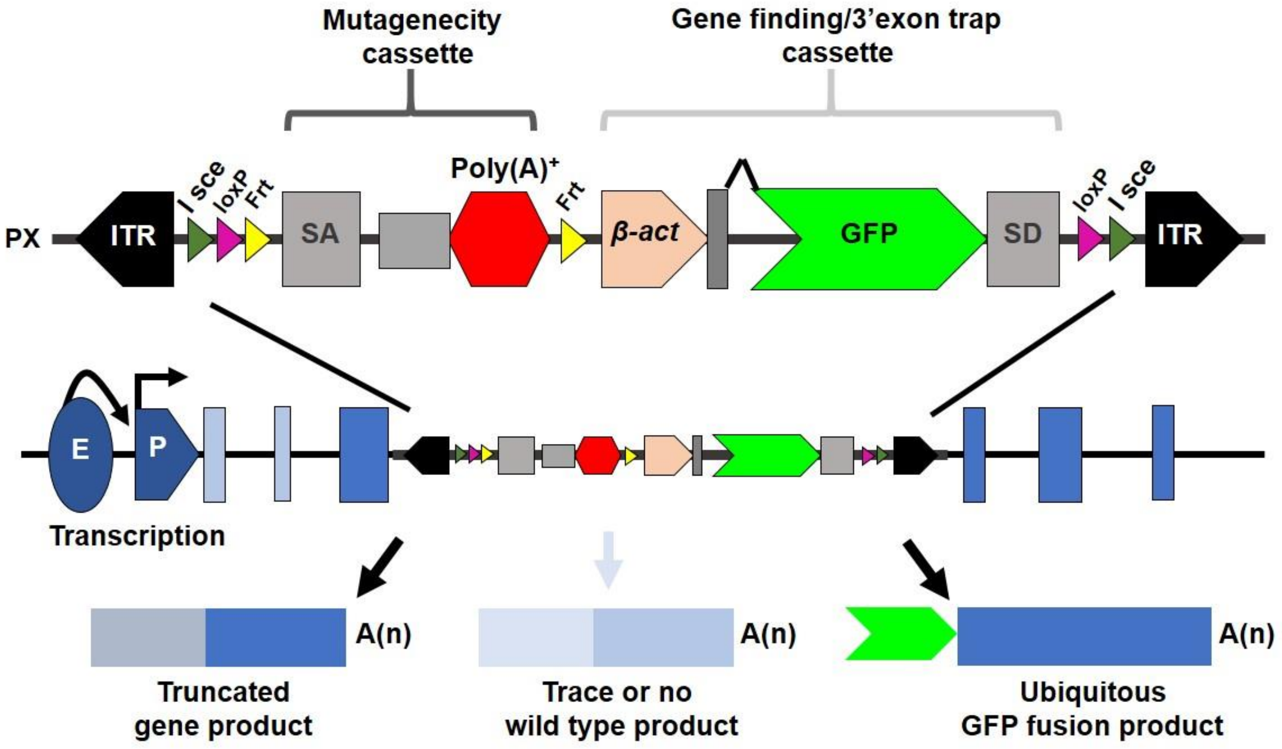

Figure S1. **Schematics of pGBT-PX gene trap vector and its integration into the genome**. Schematic representation of the pGBT-PX vector is shown. ITR: inverted terminal repeats, SA: splice acceptor, Poly(A)+: polyadenylation signal with extra transcriptional terminator and putative border element from ocean pout fish, β-act: carp beta-actin enhancer, promoter, noncoding exon, and intron 1 sequences, GFP: green fluorescent protein, SD: splice donor, Isce: intron encoding rare endonuclease recognition site, loxP: pair of short sequences for Cre mediated recombination event, Frt: FLP Recombination Target site for Flp recombinase mediated integration , E:enhancer of endogenous gene, P:promoter of endogenous gene Insertion of PX vector into the intron of a hypothetical endogenous gene is illustrated. Transcription from the ubiquitously active carp β-actin gene promoter produces a fusion transcript of the vector derived GFP sequence and the endogenous downstream exons. This fusion transcript is stabilized by the endogenously present poly(A) signal, resulting in ubiquitous GFP expression.

Fig S2

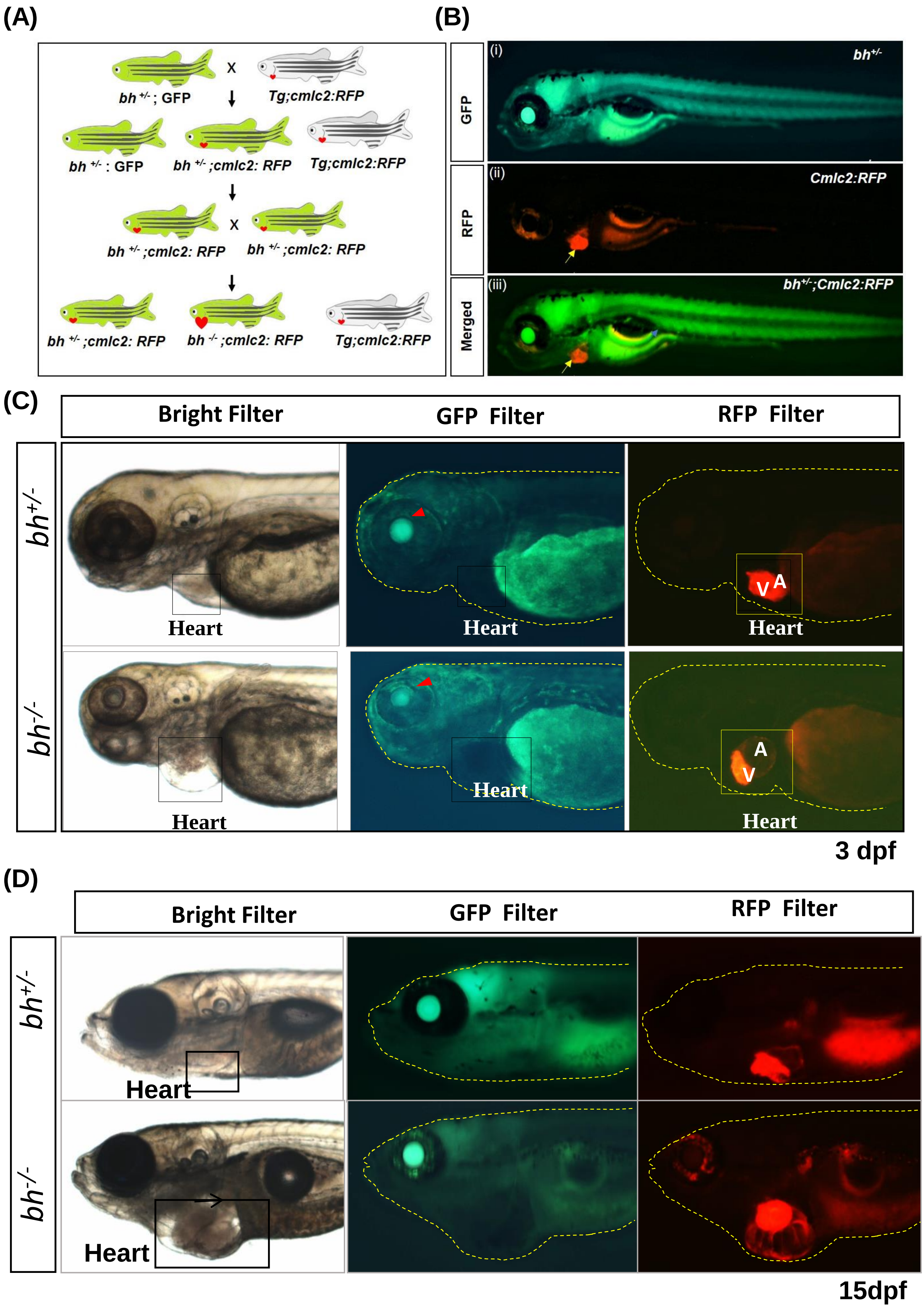
(A)
bh +/- ; GFP
x
Tg;cmlc2:RFP
bh +/- : GFP
bh +/- ;cmlc2: RFP
Tg;cmlc2:RFP
bh +/- ;cmlc2: RFP
x
bh +/- ;cmlc2: RFP
bh +/- ;cmlc2: RFP
bh -/- ;cmlc2: RFP
Tg;cmlc2:RFP
(B)
GFP
RFP
Merged
(i)
bh+/-
(ii)
Cmlc2:RFP
(iii)
bh+/-;Cmlc2:RFP
(C)
Bright Filter
GFP Filter
RFP Filter
bh+/-
bh-/-
Heart
V
A
3 dpf
(D)
Bright Filter
GFP Filter
RFP Filter
bh+/-
bh-/-
Heart
15dpf

Figure S2. Generation of *bh-/-*;cmlc2: RFP double transgenic fish. (A) Schematic showing the generation of double transgenic fish: Putative bh+/- fishes were crossed with Tg(cmcl2:RFP) fishes. Progenies expressing both GFP and RFP were selected and grown to adulthood. These fishes were intercrossed to generate double transgenic bh-/-;cmlc2:RFP fishes. (B) Representative image showing (i) GFP expression in bh+/- (ii) RFP expression in Tg:cmlc2 and (iii) double transgenic fish expressing both GFP and RFP. (C) Representative image showing the double transgenic heterozygotes and the homozygote mutant at 3 dpf. (D) Representative image showing the double transgenic heterozygotes and the homozygote mutant at 15 dpf. Yellow arrowhead indicates the heart in B. Red arrowhead in C indicates GFP expression in the eyes. A: Atrium, V : Ventricle.

Fig S3

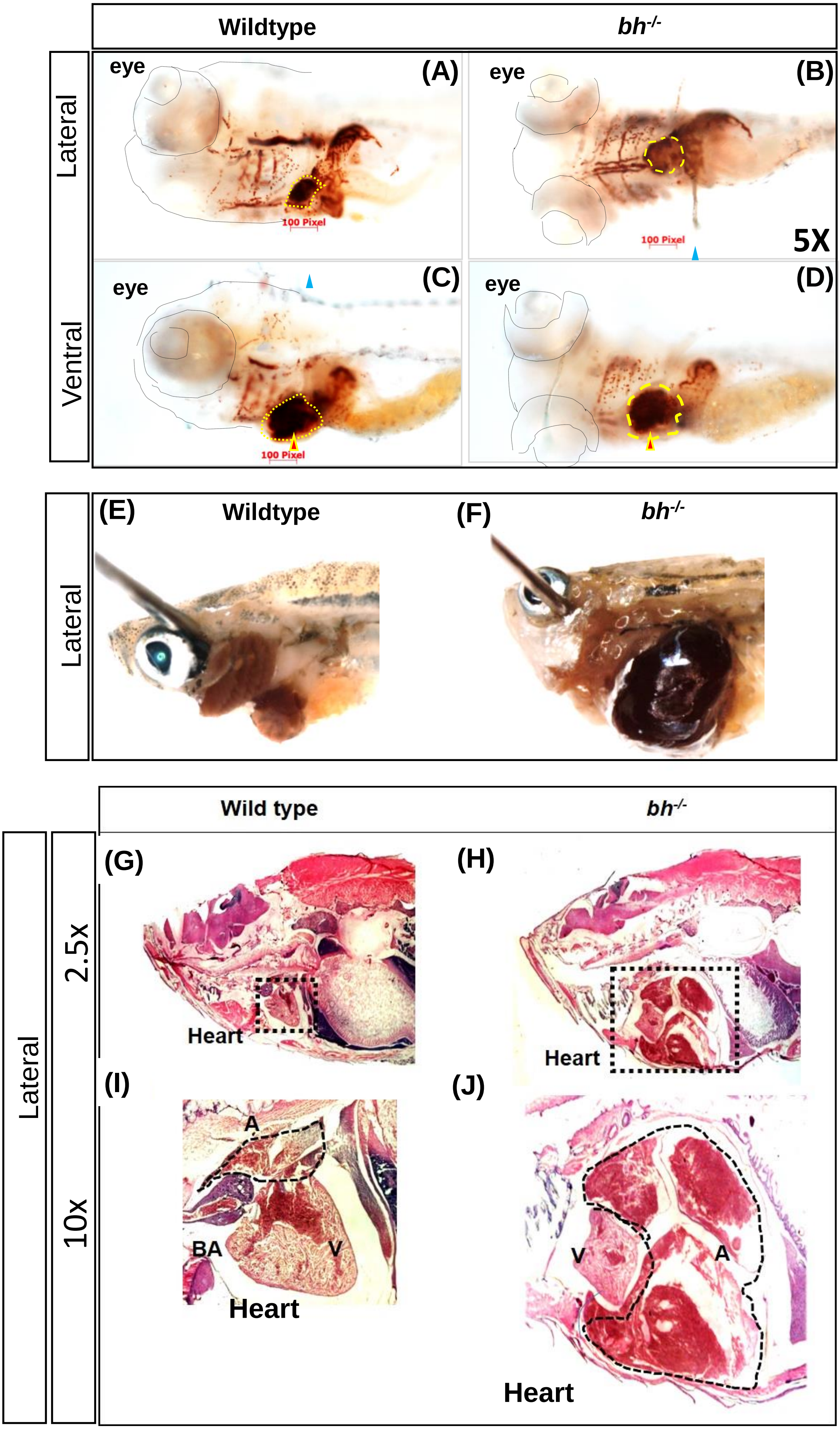

Wildtype
bh-/-
Lateral
Ventral
eye
(A)
(B)
(C)
(D)
100 Pixel
5X
(E)
(F)
Wild type
2.5x
10x
(G)
(H)
(I)
(J)
Heart
A
V
BA

Figure S3. Anatomical analysis of wild type and *bh-/-* heart revealed signs of blood accumulations and hypertrophy in the mutant. (A-D) Representative image of o-dianisidine staining of 5 dpf larvae showing the greater accumulation of blood in heart of bh-/- (B and D) which is almost twice the volume of wt siblings (A-B). Representative image of adult dissected heart showing the greater accumulation of blood in heart of bh-/- (F) as compared to the age matched wt (E). (G-J) Histological analysis of heart tissue of nine-month-old WT (G and I) and bh-/- fish (H and J) by hematoxylin- eosin (HE) staining. (G and H) Images were captured at 2.5X, (I and J) magnified images. Yellow dashed line in A-D represents the heart. The Black dashed line in I and J denotes the atrium.

Fig S4

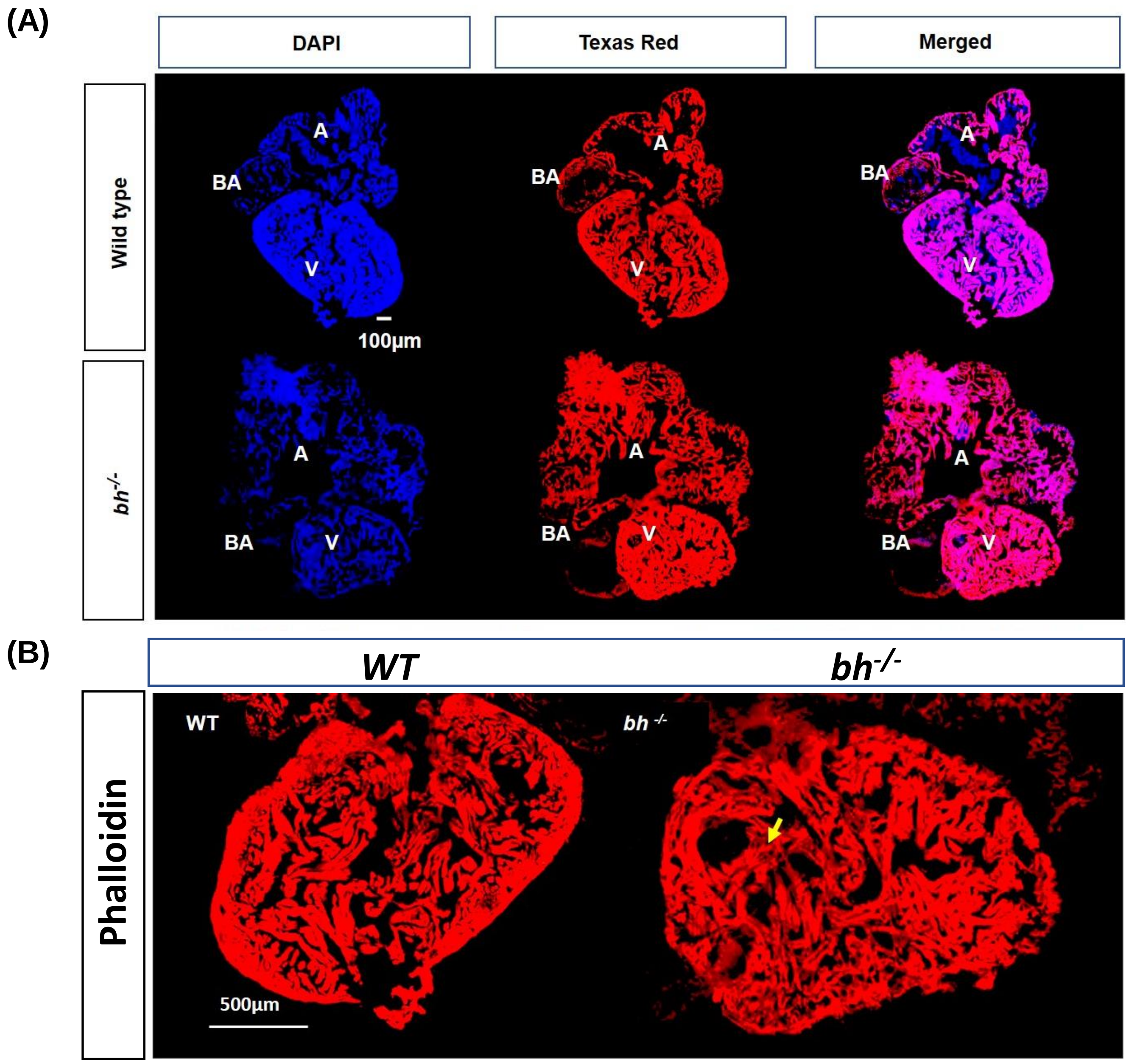

Figure S4. Phalloidin staining of wild type and bh-/- heart. (A) Nine- month old wild type zebrafish heart and bh-/- heart stained with phalloidin dye conjugated with Texas red (image captured at 2.5X). (B) Zoomed images showing a normal arrangement of ventricular cardiomyocytes in WT and muscular disarray in bh-/- fish. The images were captured using a confocal microscope (Zeiss).

Fig S5

WT *bh-/-*

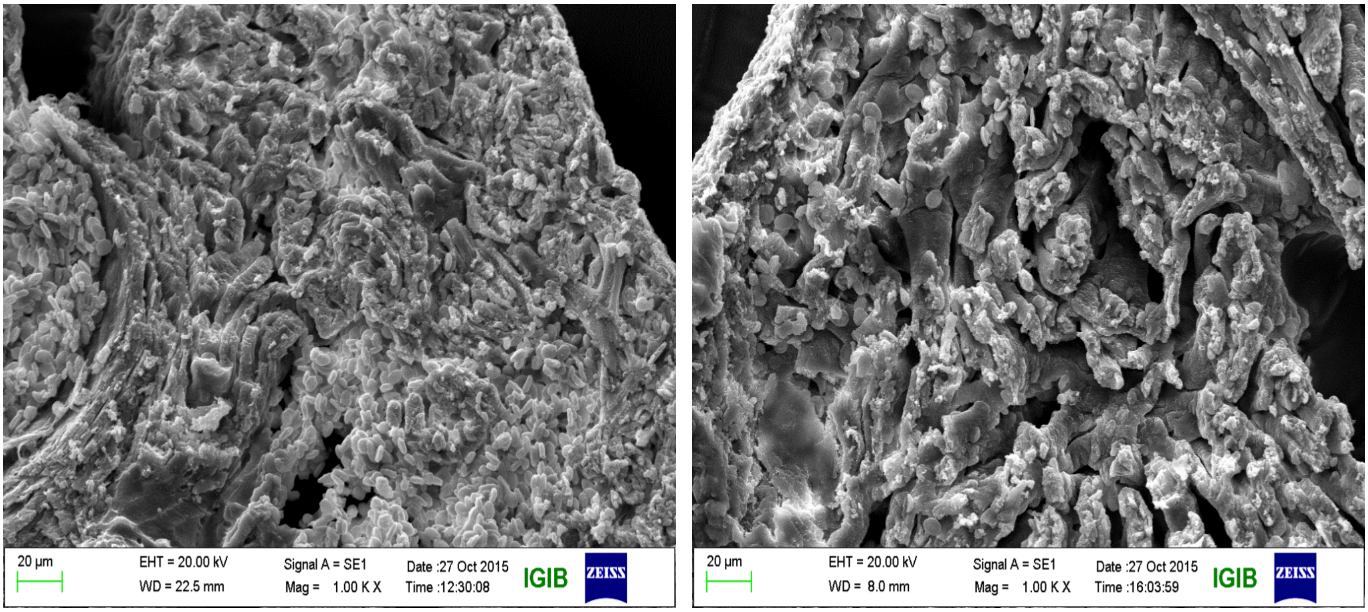

Figure S5. SEM analysis of the wild type and bh-/- heart. (A) SEM image of wildtype fish heart showing the normal arrangement of cardiac muscle and (B) SEM image of bh-/- fish heart showing muscular disarray and thickening.

Fig S6

Inverse PCR

(A)

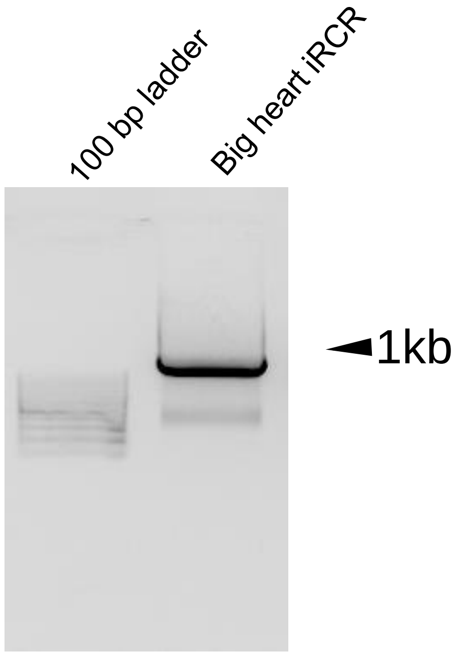

(B)

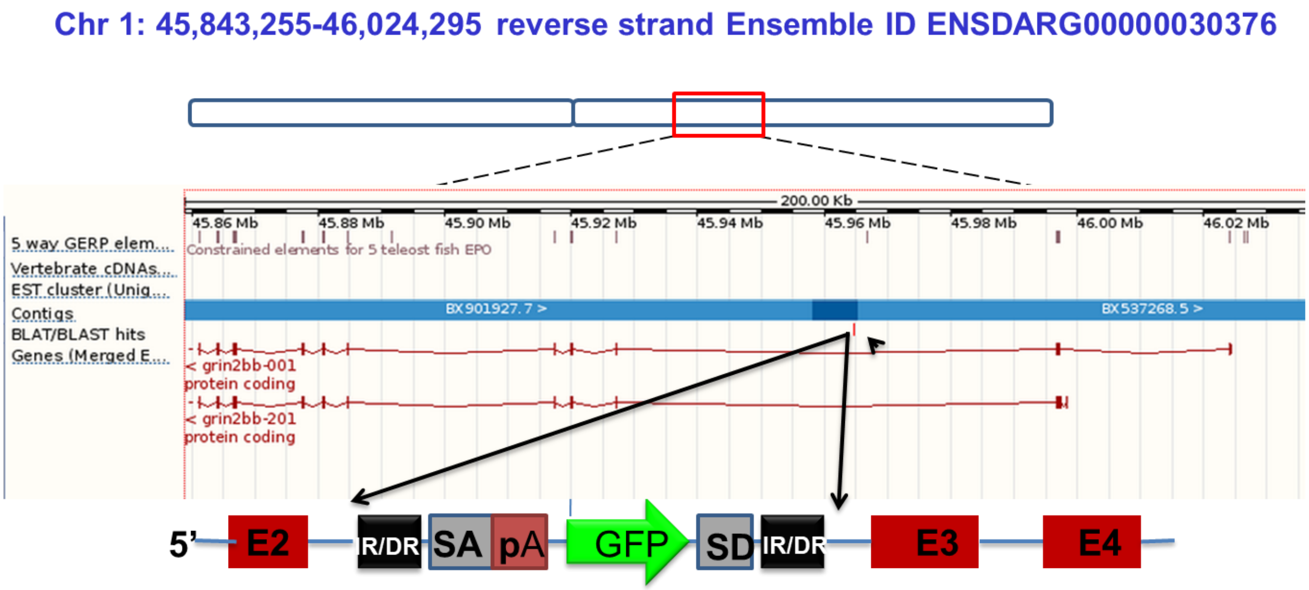

Fig S6. Inverse PCR sequence mapped to the intron 2 of grin2bb gene. (**A**). DNA agarose gel showing the inverse PCR product run on 1 % agarose gel. **(B)**. Ensemble blast result showing the location of the iPCR sequence on intone 2 of the grin2bb gene.

https://www.ensembl.org/Danio_rerio/Gene/Summary

Fig S7

(A)

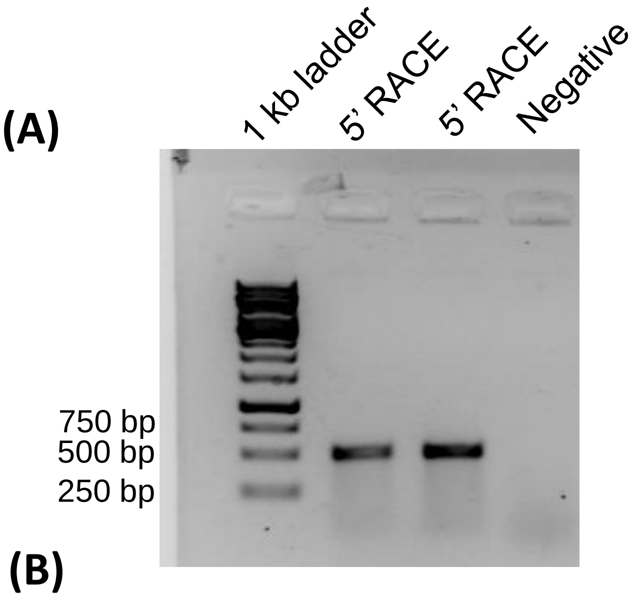

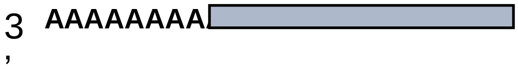

(B)

```
241  GCCCTGTGACGTCCACGGGCCACTCGTGCACCGTTTGTGTCTCGTGGCCAGGGGGATTTC   300
301  CTTCTGTTGCTCCAGTACTGCCGAGAGGCGTCTGATATTTCCATAACATCAAACCACCCC   360
361  GCGTCAAAGGGGTTCTTTTTTATTTTTTCCCCCCCGGGGTGAATGGGGGGGGGGGGGGGG   420
421  GGATTTTATATTATTATATAGCCATTTTGAATTTTATGAAAAGAAATTAAGGAAACTTTT   480
481  ATGCTTTGTAGATTCACATTCAATCCAGTGCTTTGTAATTATTTGTGAAATCTACTCACA   540
541  ACTGCAACACCGGTTCATTTGGAAAACGTAGCCCCATATACATTTCTGGAGATCACAAAT   600
601  TAAGTAGCCAGAAGTACGTACACTGTATGGCTGCATTTGTCTTTAAAATGAACGGGCGGT   660
661  ATGATGATGTTCCTTATGTGCCTTTACTCACTTACTGCTTACCTCTGTACGGCTCCGTAA   720
721  AGCTTCTGTACGGCTTTTCCGGCTGTTATCCAGTTGTCCTTACCTTGGTT             770
```

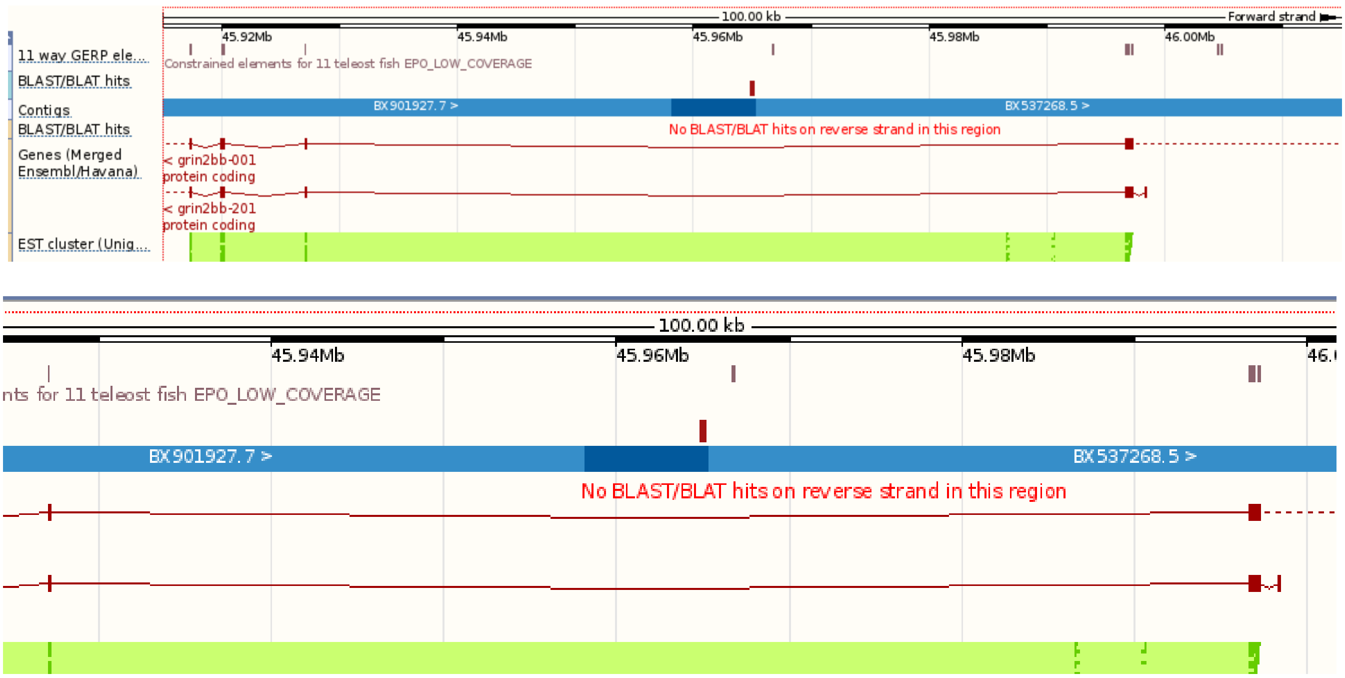

Genomic Location	Orientation	Length	Score	E-val	%ID
1:45964835-45965164 [Sequence]	Forward	333	611.0	2.8e-175	99.10

https://www.ensembl.org/Danio_rerio/Gene/Summary

Fig S7. 5' RACE product mapped to the intron 2 of grin2bb gene. (A). 5'RACE product run on 1.5% DNA agarose gel. (**B**). Ensemble blast result showing the location of the 5'RACE sequence on introne 2 of grin2bb gene.

Fig S8

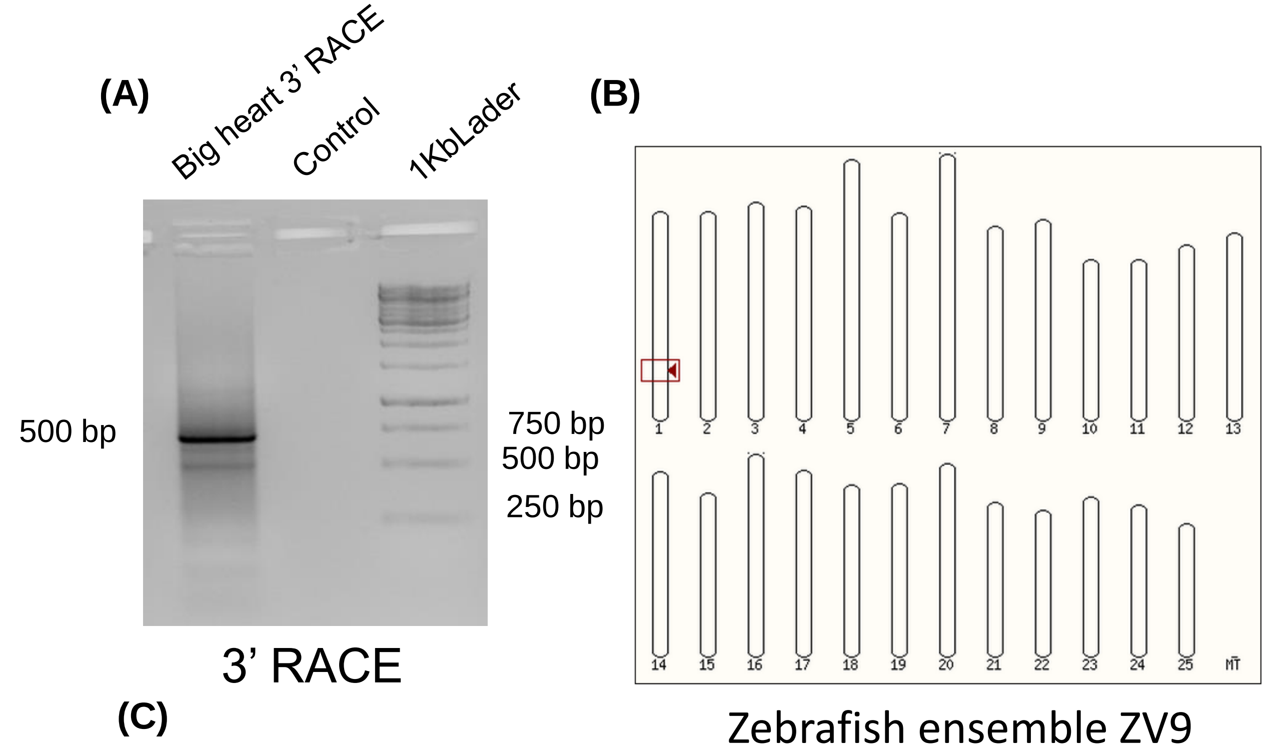

(C)

Query			Chromosome				Stats			
Start	End	Ori	Name	Start	End	Ori	Score	E-val	%ID	Length
1	118	-	Chr:1	45964700	45964817	+	549	6.8e-89	95.76	118

```
AGATCACAAATTAAGTAGCCAGAAGTACGTACACTGTATGGCTGCATTTGTCTTTAAAAT
GAACGGGCGGTATGATGATGTTCCTTATGTGCCTTTACTCACTTACTGCTTACCTCTGTA
CGGCTCCGTAAAGCTCTGTACGGCTTTTCCGCTGTTATCAGTTTGACCAATACCTCGTTA
TATAGGTCAGTAGTCTTGAAACGCAGAGAGTTCACTACAAATGGACTTCTTTGAGTCTGT
TGAAGAATGGTTCCAGAAAGCAGCTTTAAGAAAATCTAAGCCAAAAAAAAAAAAAAAAAC
AAGTAAATAACATGTTGAAAATGAGGTAAAATCTGAAACTAAGGTAAACATCAGGCAAAG
GCTTTGCTTTTTGGGTGGGTGGTTCAATCTGTGATTATAAAAAACATTATTAGTTGGGTT
```

Links	Query			Chromosome				Stats			
	Start	End	Ori	Name	Start	End	Ori	Score	E-val	%ID	Length
[A] [G] [C]	1	109	-	Chr:1	45964707	45964815	+	529	2.9e-85	99.08	109
[A] [G] [C]	51	83	-	Chr:13	44180355	44180387	+	158	1.9e-19	96.97	33
[A] [G] [C]	48	81	+	Chr:2	15121998	15122031	+	158	1.9e-19	97.06	34
[A] [G] [C]	48	81	-	Chr:5	50228388	50228421	+	158	1.9e-19	97.06	34

Fig S8. 3' RACE product mapped to intron 2 of grin2bb gene: (**A**). DNA agarose gel showing the 3'RACE PCR product. (**B and C)** Ensemble blast result of the 3'RACE sequence.

https://www.ensembl.org/Danio_rerio/Gene/Summary

Fig S9

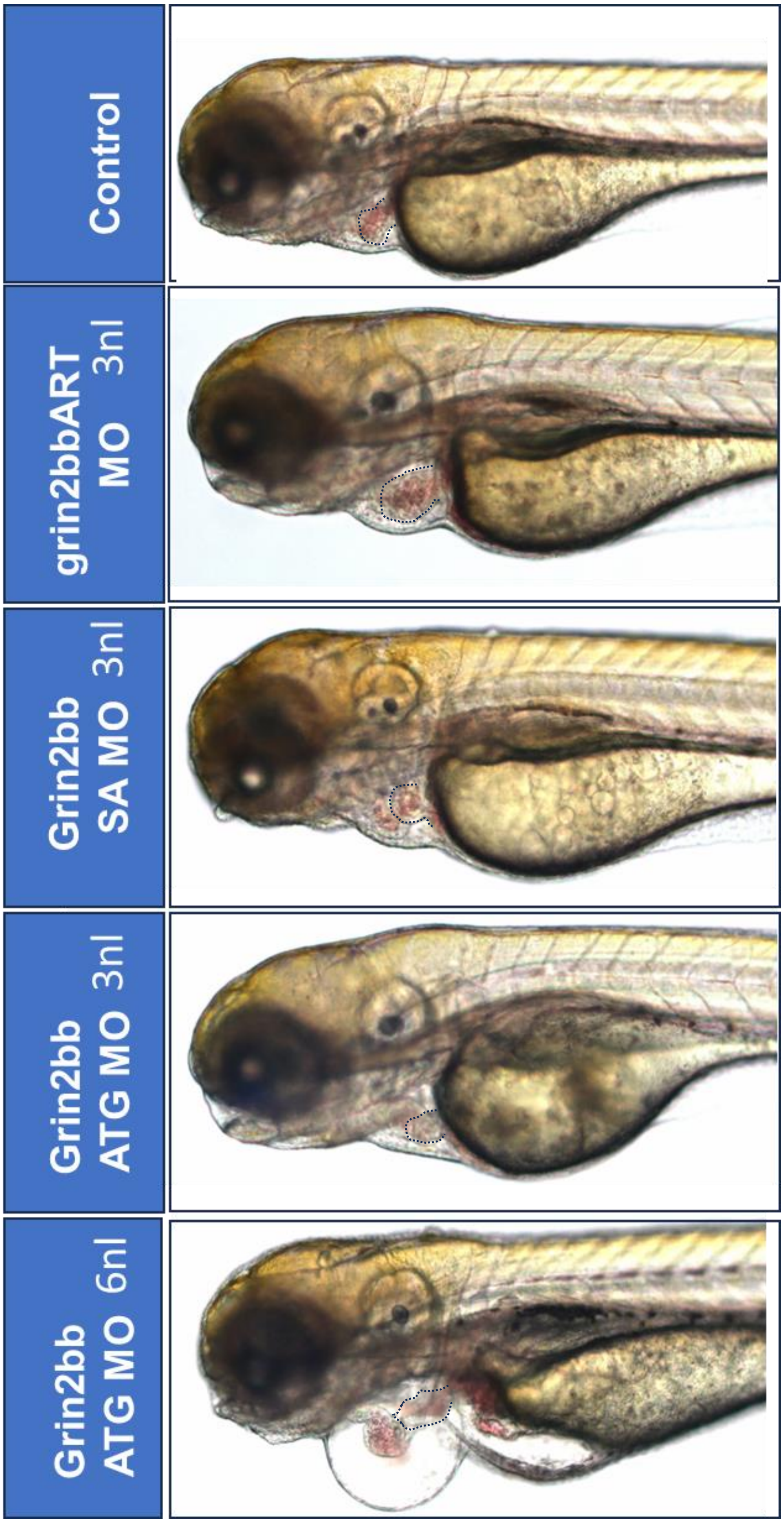

Figure S9. Representative image of 3 dpf zebrafish embryos injected with 100 uM of grin2bbART MO (MO1+MO2), grin2bb splice junction targeted MO (MO1 + MO2) and grin2bb ATG blocking MO. Images were captured using a Zeiss Axioscope microscope with 5X magnification. N=5 embryos were analyzed for each group. Dotted outline represents the atrium.

Fig S10

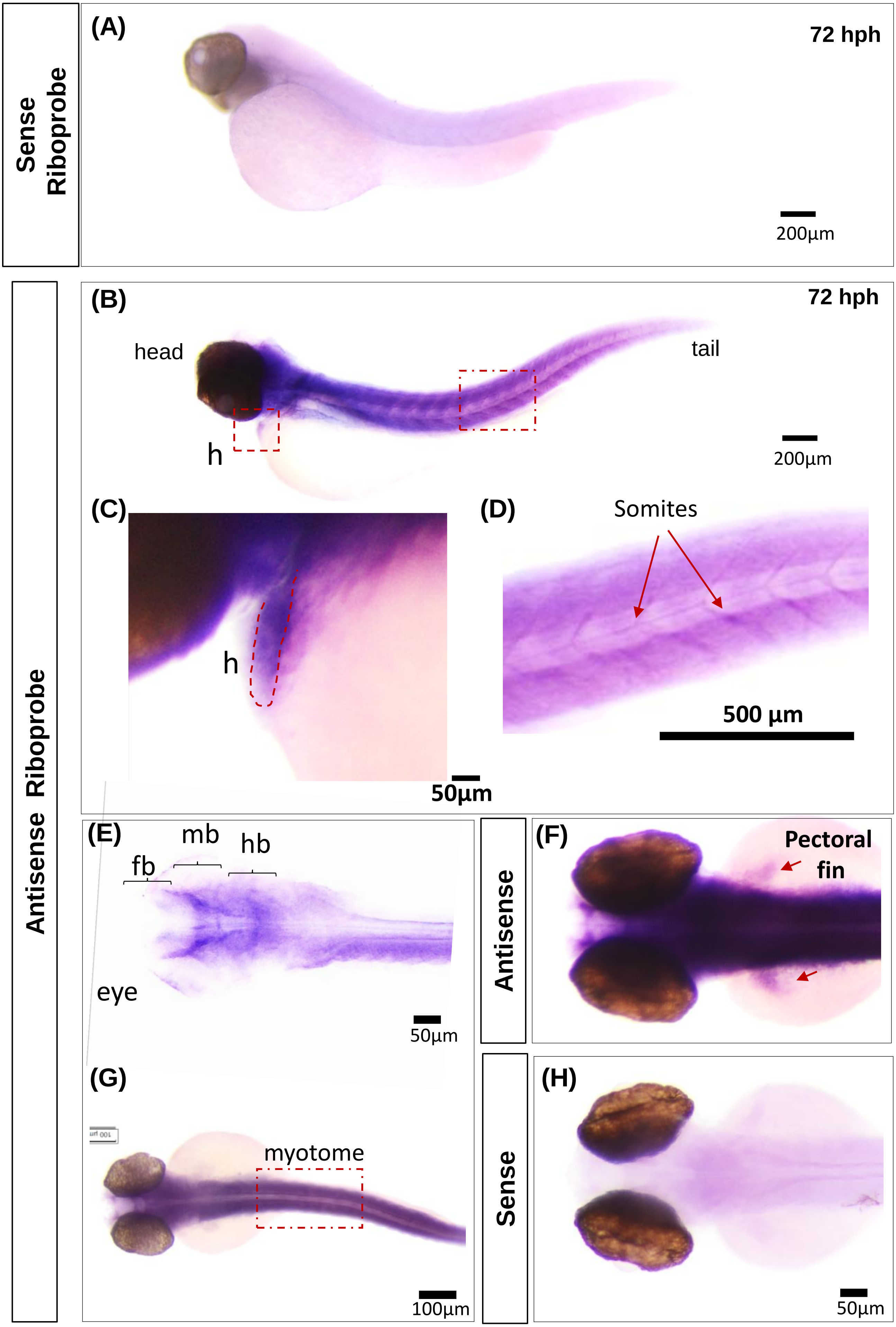

Sense Riboprobe
(A)
72 hph
200µm
Antisense Riboprobe
(B)
72 hph
head
tail
h
200µm
(C)
h
50µm
(D)
Somites
500 µm
(E)
fb
mb
hb
eye
50µm
(F)
Antisense
Pectoral fin
(G)
myotome
100µm
(H)
Sense
50µm

Figure S10. Whole-mount in situ hybridization showing grin2bbART expression in 3 dpf zebrafish embryos. (A) Representative image showing 3 dpf zebrafish embryo labeled with grin2bbART sense (control) probe. (B) Representative image showing 3 dpf zebrafish embryo labeled with grin2bbART specific antisense probe. (C) grin2bbART expression in heart of 3 dpf embryo. (D) grin2bbART expression in the trunk (somite borders. (E) Dorsal view of head showing grin2bbART expression in the brain. (F) Dorsal view of head showing expression of grin2bbART specific probe after extended staining. (G) Dorsal view of the complete 3 dpf zebrafish showing grin2bbART expression in the brain and myotome. (H) Dorsal view of the Dorsal view of head (control). MB: midbrain and brain boundary, fb: forebrain, hb: hindbrain. h: heart. The experiment was repeated two times, and n=10 embryos were analyzed per developmental stage for each probe.

Fig S11

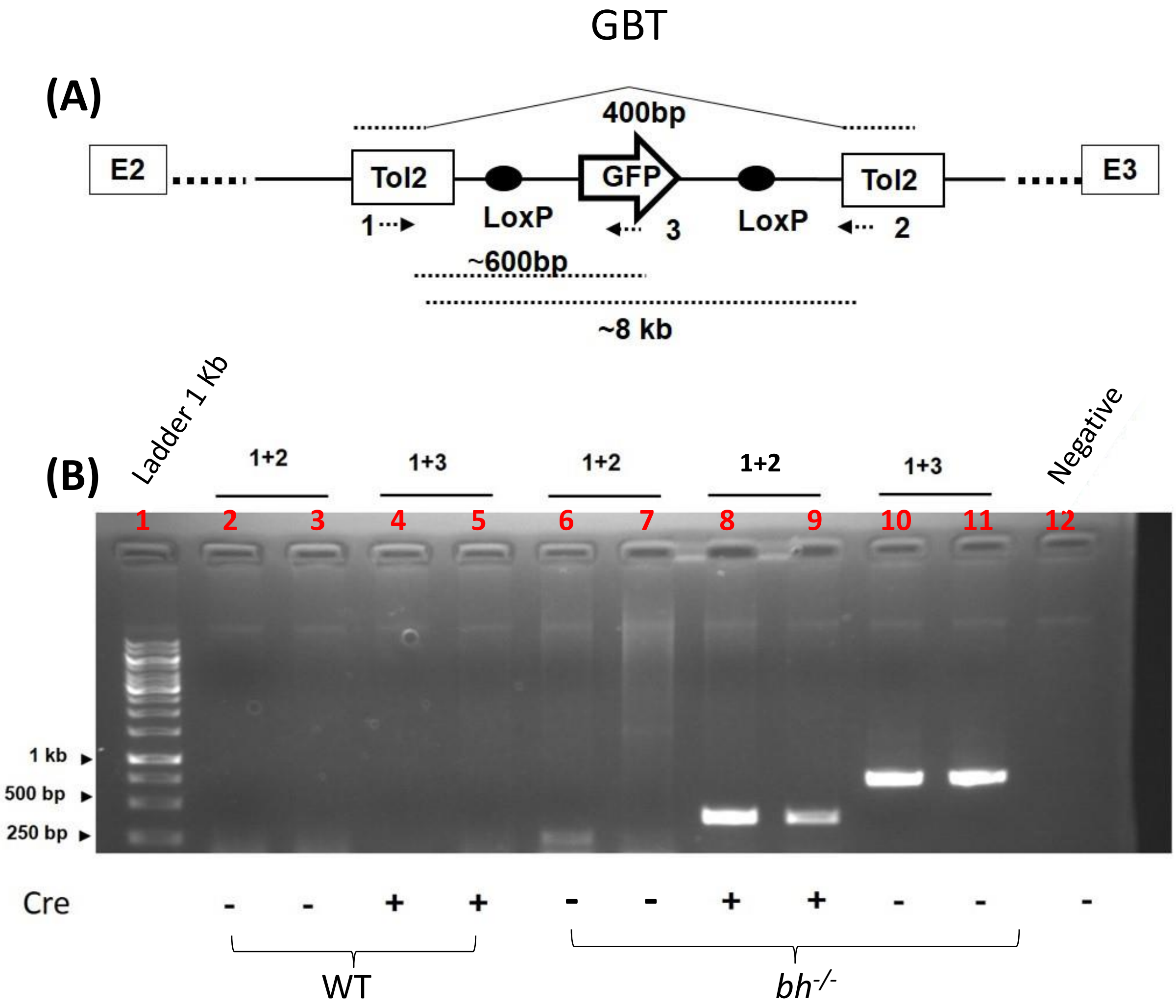

Figure S11. Cre/Lox mediated GBT excision in the *bh-/-* genome. (A). Schematic showing the exons (box) of *grin2bb* transcript and the positions of the primers (arrowheads). (**B)** DNA agarose gel showing the PCR products corresponding to various primer combinations in the samples with or without Cre recombinase. The GBT insertion is absent in wild type (Lane 2-5); hence, there are no PCR bands. In *bh*$^{-/-}$, the Cre/lox mediated excision of GBT causes a smaller band (Lane 8 and 9). In the absence of Cre/lox, a band size of 600 bp is observed.

Fig S12

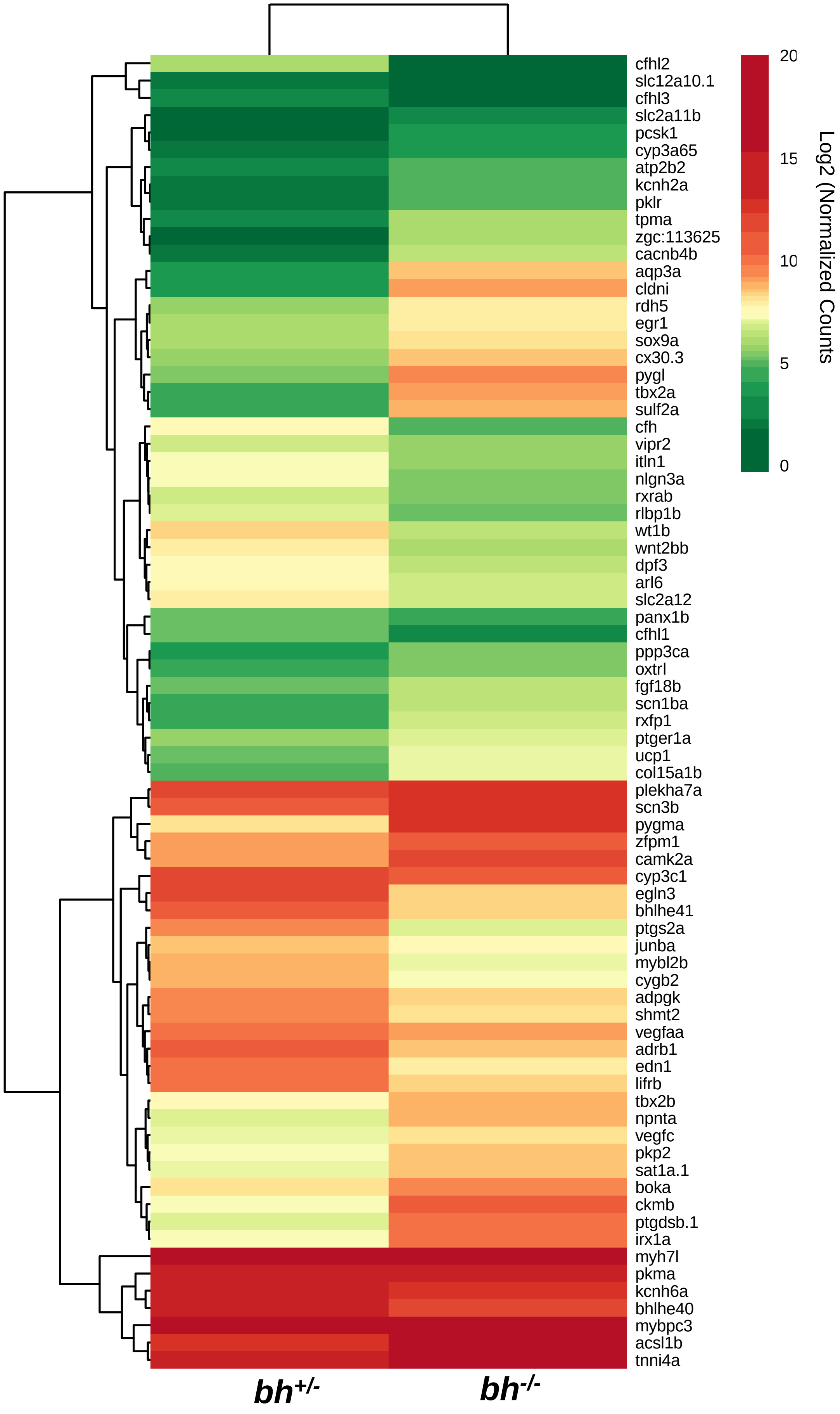

Figure S12: Bigheart shows differentially expressing cardiac transcriptome.
Total 76 cardiac DE genes at FC >2 and Norm. counts >0 in both samples

Fig S13

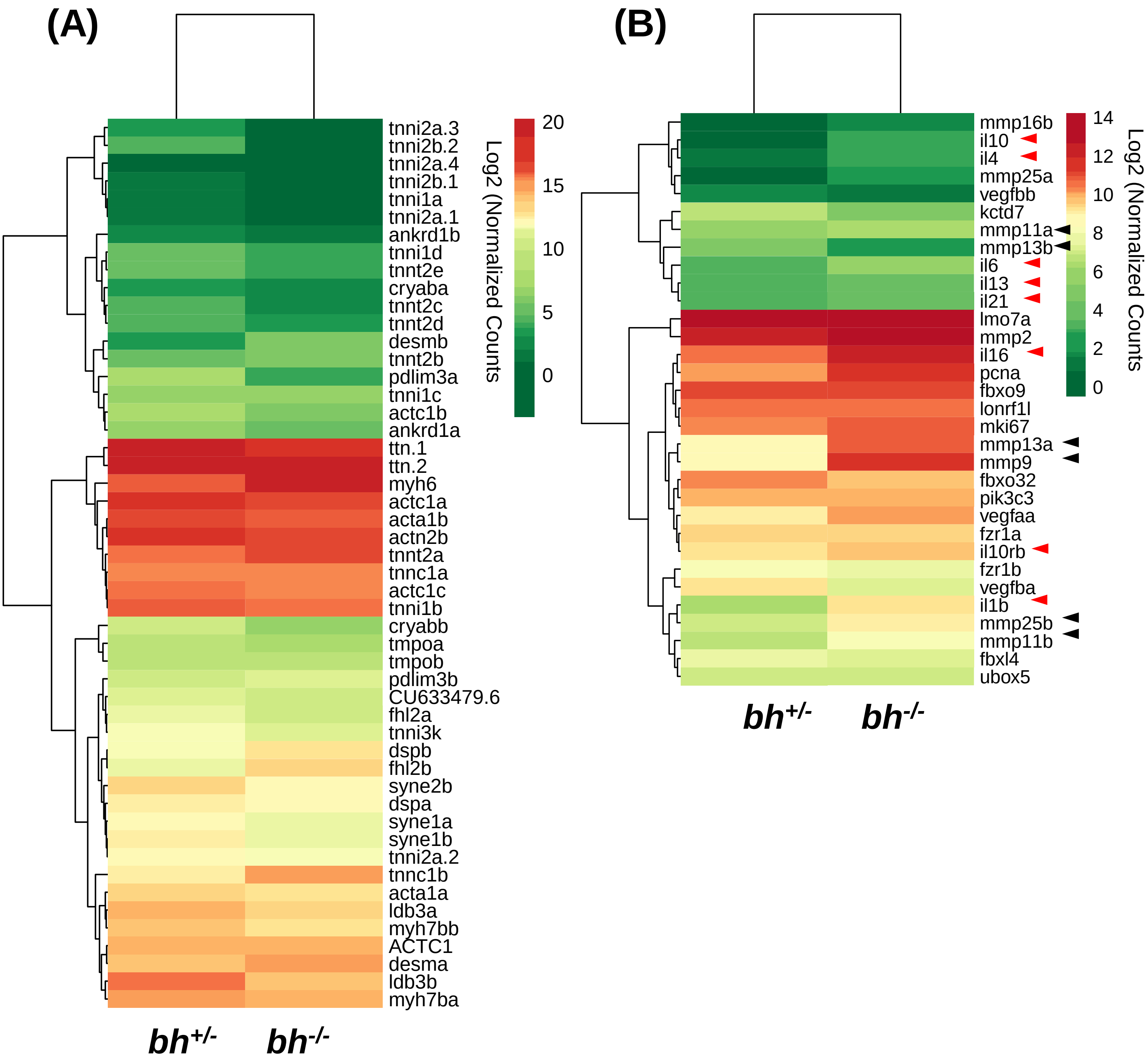

Figure S13: Bigheart shows differentially expressing cardiac transcriptome. (A) Heat map showing differentially expressed gene implicated in cardiac disease including dilated cardiomyopathy and hypertrophy**. (B)** Heat map showing differentially expressed gene implicated inflammation and ECM. Values are presented in log_2fold change (FC >2 and Norm. counts >0 in both samples).

Fig S14

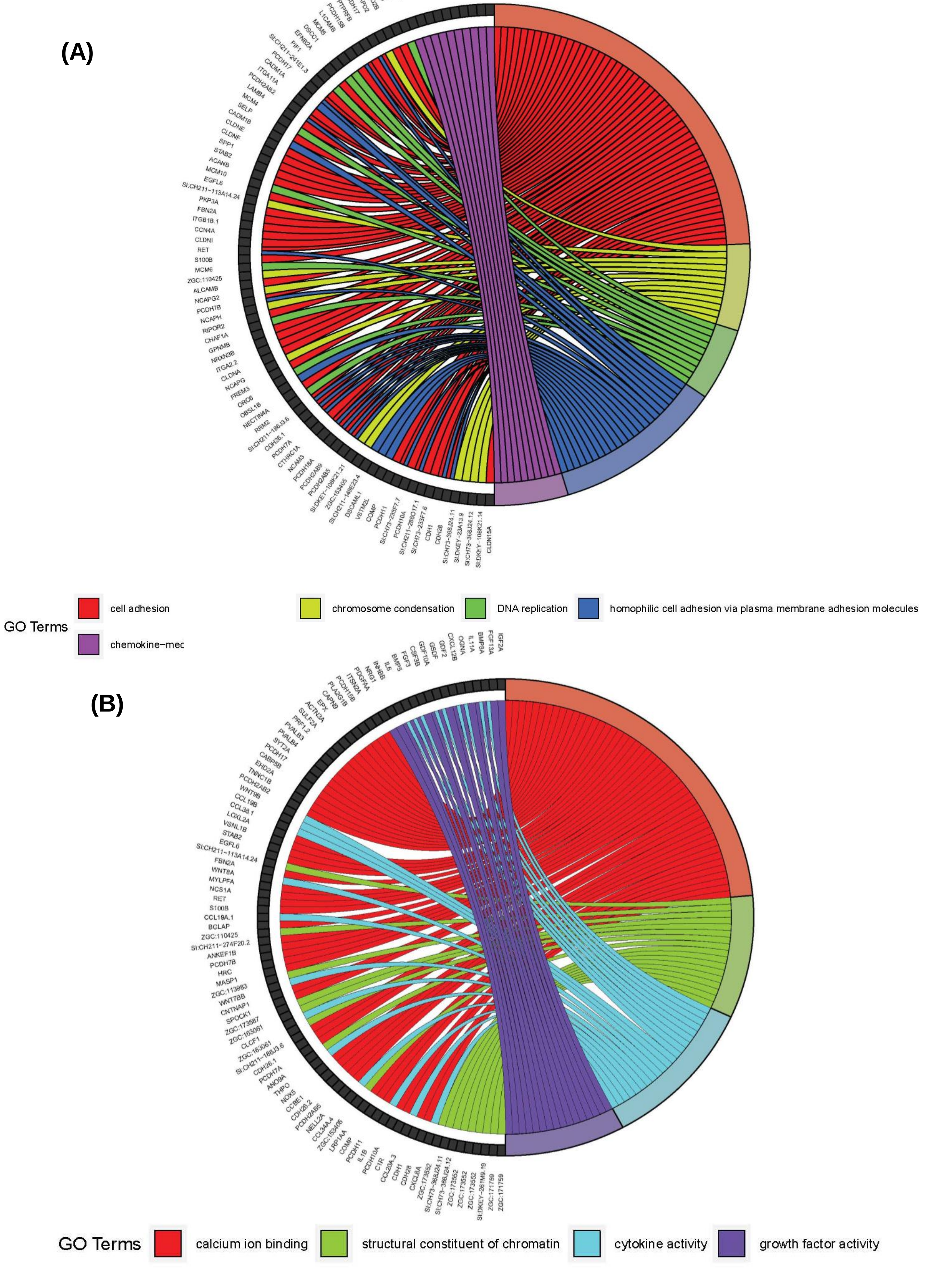
(A)
GO Terms
cell adhesion
chemokine−mec
chromosome condensation
DNA replication
homophilic cell adhesion via plasma membrane adhesion molecules
(B)
GO Terms
calcium ion binding
structural constituent of chromatin
cytokine activity
growth factor activity

Figure S14. Gene ontology enrichment analysis of significantly changed genes between *bh*$^{+/-}$ and *bh*$^{-/-}$. (A) The chord plot presents the linkages of genes and GO biological process terms. (B) The chord plot presents the linkages of genes and GO Molecular function terms.

Fig S15

(A)

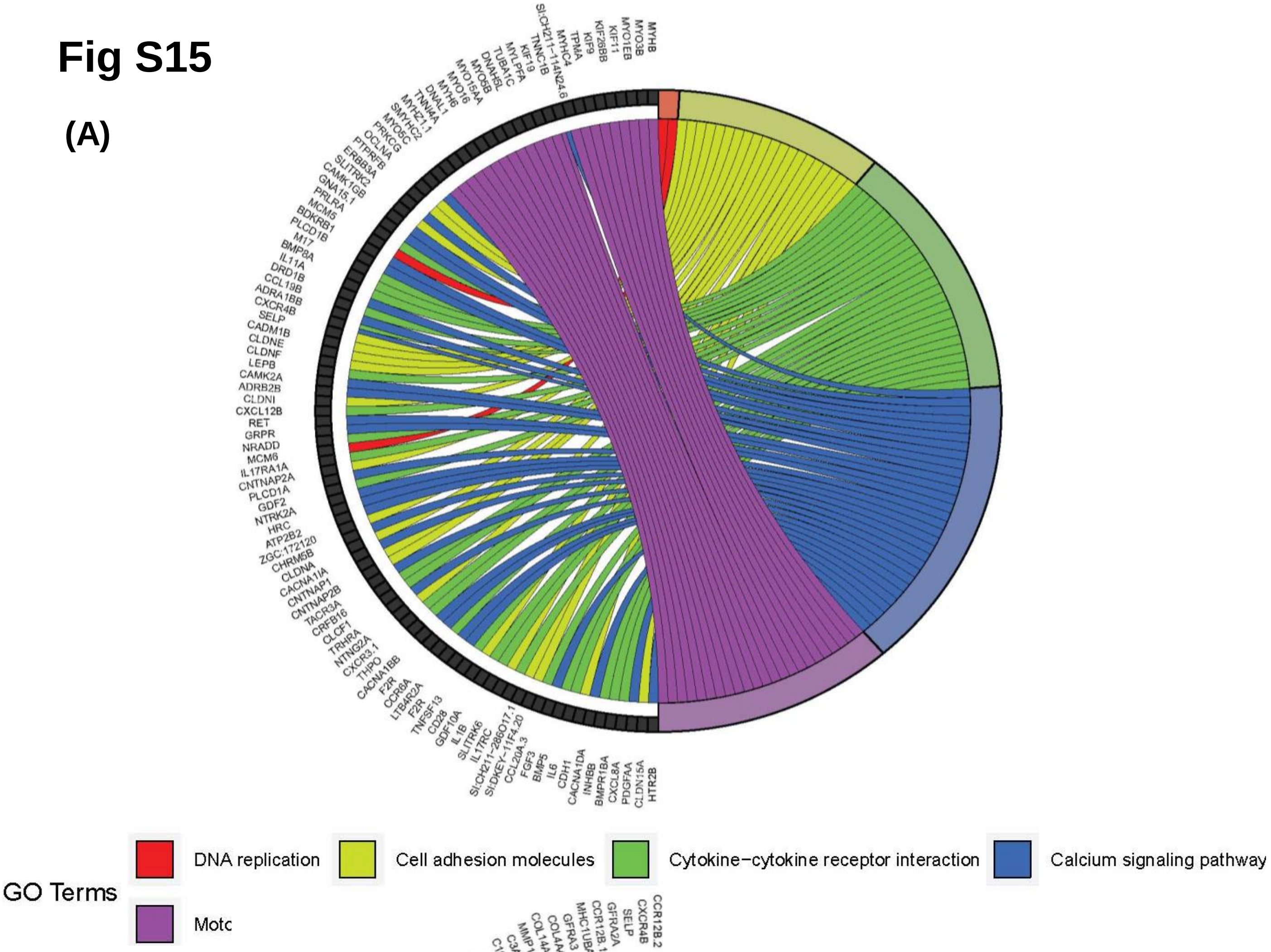
GO Terms
DNA replication
Cell adhesion molecules
Cytokine−cytokine receptor interaction
Calcium signaling pathway
Motc

(B)

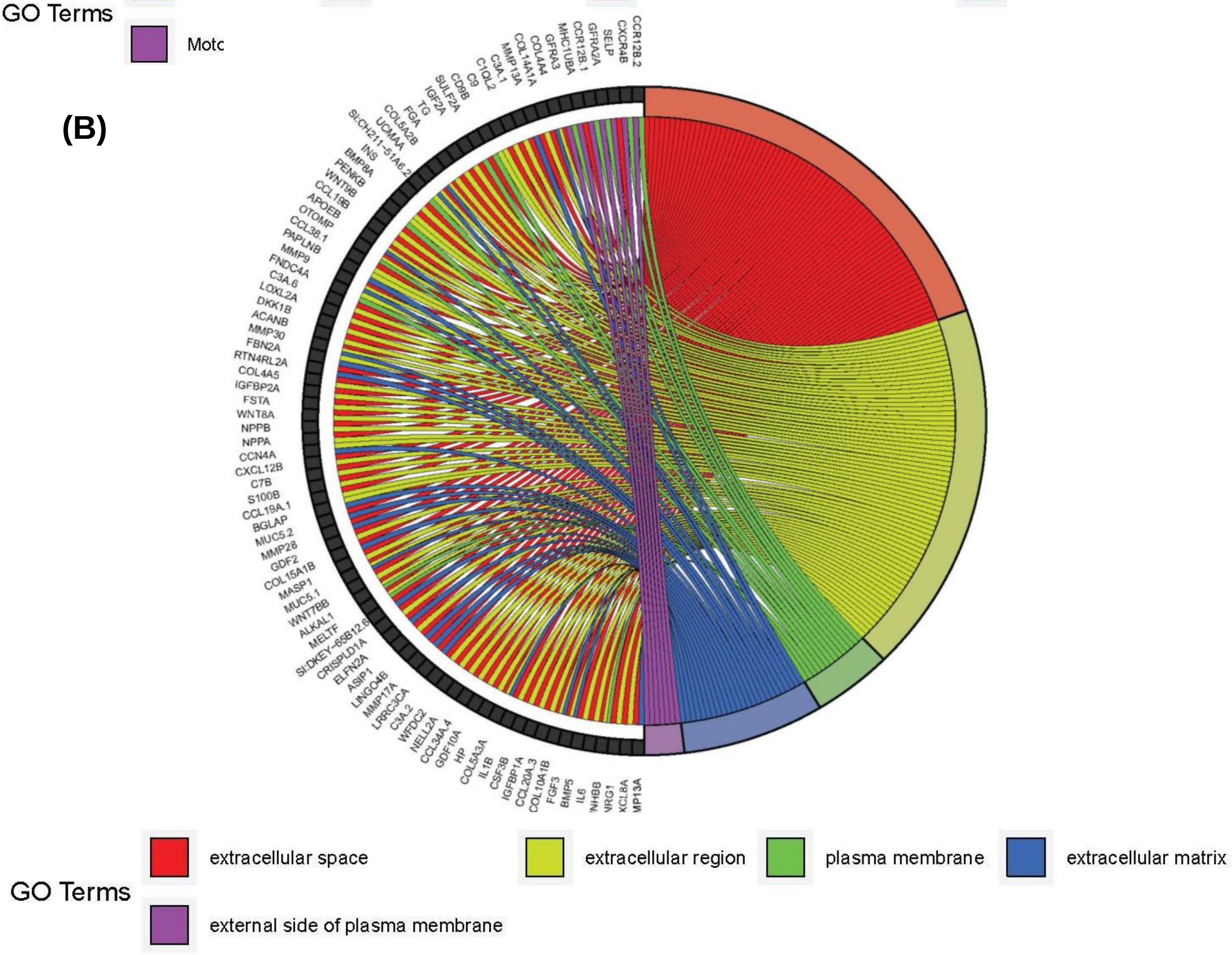
GO Terms
extracellular space
extracellular region
plasma membrane
extracellular matrix
external side of plasma membrane

Figure S15. Gene ontology enrichment analysis of significantly changed genes between *bh*$^{+/-}$ and *bh*$^{-/-}$. The chord plot presents (A) the linkages of genes and KEGG analysis (B) GO terms Cellular components.

Fig S16

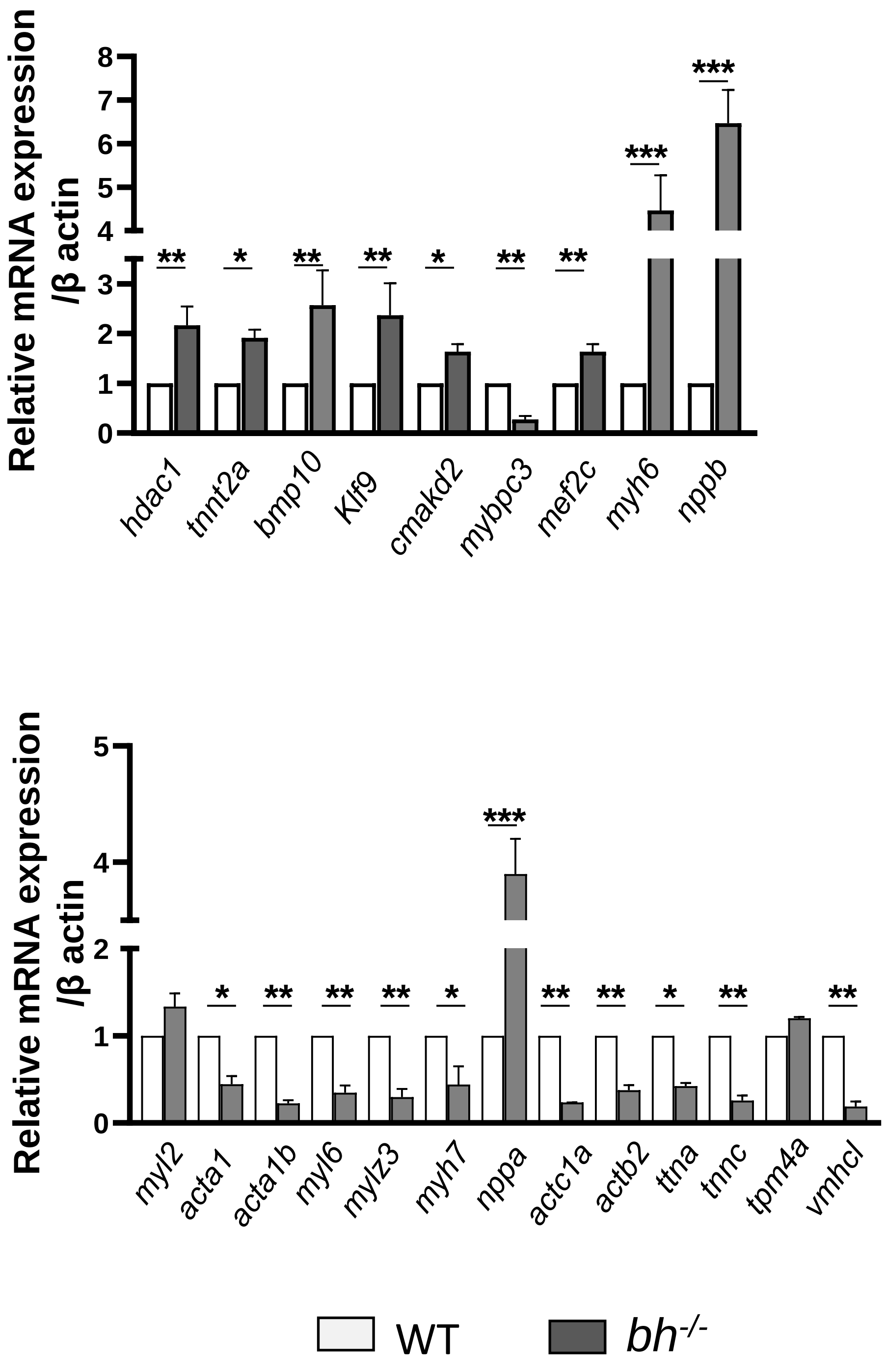

Figure S16. Validation of critical cardiac genes identified in the RNA sequencing by qRT PCR.
