# Supplementary material for: Forward genetic screen using a gene-breaking trap approach identifies a novel role of grin2bb-associated RNA transcript (grin2bbART) in zebrafish heart function
Source: Front Cell Dev Biol. 2024 Mar 8;12:1339292. doi: 10.3389/fcell.2024.1339292 (PMC10964321; doi:10.3389/fcell.2024.1339292)
Supplement: Supplementary file 10 [file DataSheet1.PDF]

# Sequence

## Big heart (GBT trapped PX-Line)

## (A) Inverse PCR sequence

### Sequencing with GFP nest (forward primer)

### AP026/AP_697_SSB

GGGATGGACTTCAATAATGTCCAGACCTGCAGGAATTCGATATCAAGCTTATCGATGAACAAACCCCCCCAAAC
CTCAGGTGAGTTGATCTTTAAGCTTTTTACATTTTCAGCTCGCATATATCAATTCGAACGTTTAATTAGAATGT
TTAAATAAAGCTAGATTAAATGATTAGGCTCAGTTACCGGTCTTTTTTTTCTCATTTACACTAGCTAAGCATGC
TGATAACTTCGTATAGCATACATTATACGAAGTTATTACCCTGTTATCCCTACAAATTAAACTGGGCATCAGCG
CAATTCAATTGGTTTGGTAATAGCAAGGGAAAATAGAATGAAGTGATCTCCAAAAAATAAGTACTTTTTGACTG
TAAATAAAATTGTAAGGAGTAAAAAGTACTTTTTTTTCTAAAAAAATGTAATTAAGTAAAAGTAAAAGTATTGA
TTTTTAATTGTACTCAAGTAAAGTAAAAATCCCCAAAAATAATACTTAAGTACAGTAATCAAGTAAAATTACTC
AAGTACTTTACACCTCTG – **GBT-PX Vector Sequence**
TATAGGTCAGTAGTCTTGAAACGCAGAGAGTTCACTACAAATGGACTTCTTTGAGTCTGTTGAAGAATGGTTCC
AGAAAGCAGCTTTAAGAAAATCTAACCCAAAAAAAAAGAAAACCGTATAACACCTTTGTGAAAGAAGC-
**insert**

### iPCR product (AP039) sequenced with reverse primer SSB-536

AATCTCTCTTTACTCATTTTTTCTACCGGTACCCGGGGATCCTCTAGTAGTTCTAGATCTCTAAATGCATATTTTTA
AATACAATAAAAATATATTGTAATATATATTATCATTTCCTTCATGCTGATTTTCTTTAGAAAAAAAACTATAACAC
TTACACCCCAAAAAAATAAAAGAAAATCAACTAATTATATAACAATAAAAAAACAACAAAATTATATTCTTAAAGTT
TTTTTATATATATAATTATATCATTCATGCTGATTTTCTTTTGCTTTTTTTATTAGTGTGACGGGGTTTTTTTCCCA
AAAATTCCAGAAAATTGTAAATATACGCTTACTCAATTATAGAAATCTTTCTACAACAAATGATTGACTGACTAATT
TTTTGCAGCTTTTGTTTTTGTGAACCTATCAGAGGCCACTGTGTACACTTTTCAAATTTAAAATTTCTCTCGCGAGA
GCCATTCGCACCCGCTGTTCTCGCATGAGGCTGCCATTGACTGACTGACTGACCAATCGACTATCCTAAACCCAACT
AATAATGTTTTTTATAATCACAGATTGAACCACCCACCCAAAAAGCAAAGCCTTTGCCTGATGTTTACCTTAGTTTC
AGATTTTACCTCATTTTCAACATGTTATTTACTTGTTTTTTTTTTTTTTTGGGGCTAAATTTTCCAAAAGGGGGTTT
TTGGGGACCTTTTTTTAAAAAAACAAAAAAAACCCCTTTTGGGGGGAAACCCCCGGGGTTTCAAAAACCCCGACCTT
AAGGGGGGGAAAAGCCCTGGGAAATTTTCCCTGGTTACCGGCCCCTAAGTATTTTTTGGGGGGGTTTTTCTTCTCCT
TGAGAACAAAAAAAACAAAAACTTTACCTTTACCAATTCCTTTTTTAAAAAAAAAAAATCCTTCTCACCCCCCTCAAT
TTTTTTCCATCAAGAGATTATTTTTTTGGAAAACACTCTTCTATTTCCCCGTGCCTACACCACACAATAAGAATGGC
GCTATCCCAATTATATTGTGAGGGTAACGGGGATACACTCGGATATGGCTGCTAACAGATTTTCGCCGTCTTCGTGT
GTGTATTGACAGAAAAACCAGACTAGACACAGTCTTGTGCTGCGTTTATCCATTCTTAACTGAAGAGTAGCGAGGAG
ATGAATGACGTAATA

## (B) 3′RACE sequence

### Sequencing with GFP nest primer

AGACTATTTCACTAAATGTCCAGACCTGCAGGAATTCGATATCAAGCTTATCGATGAACAAACCCCCCCAAACC
TAAGGTGAGTTGATCTTTAAGCTTTTTACATTTTCAGCTCGCATATATCAATTCGAACGTTTAATTAGAATGTT
TAAATAAAGCTAGATTAAATGATTAGGCTCAGTTACCGGTCTTTTTTTTCTCATTTACACTAGCTAAGCATGCT

GATAACTTCGTATAGCATACATTATACGAAGTTATTACCCTGTTATCCCTACAAATTAAACTGGGCATCAGCGC
AATTCAATTGGTTTGGTAATAGCAAGGGAAAATAGAATGAAGTGATCTCCAAAAAATAAGTACTTTTTGACTGT
AAATAAAATTGTAAGGAGTAAAAAGTACTTTTTTTTCTAAAAAAATGTAATTAAGTAAAAGTAAAAGTATTGAT
TTTTAATTGTACTCAAGTAAAGTAAAAATCCCCAAAAATAATACTTAAGTACAGTAATCAAGTAAAATTACTCA
AGTACTTTACACCTCTG – **GBT-PX vector sequence**
TATAGGTCAGTAGTCTTGAAACGCAGAGAGTTCACTACAAATGGACTTCTTTGAGTCTGTTGAAGAATGGTTCC
AGAAAGCAGCTTTAAGAAAATCTAAGCCAACAGAAAAAAAAAGATAATTTTTCTGCGGGGGCAACAACCCGGGA
GAGGCCATGTGGTTCTTTTGTGAGAGAAAAAAAGAGACGCTCATA – **insert**

# 3′RACE sequence cloned in Topo TA vector

## Sequencing with M13 universal primers

## Forward seq/AP03/AP_676_SSB

CGCATTCTACGTATACGGCGATTGATTTAGCGGCCGCGAATTCGCCCTT ---- **Topo TA vector**

**Insert-**
GGCCACGCGTCGACTAGTACTTTTTTTTTTTTTTTTTGGCTTAGATTTTCTTAAAGCTGCTTTCTGGAACCATT
CTTCAACAGACTCAAAGAAGTCCATTTGTAGTGAACTCTCTGCGTTTCAAGACTACTGACCTATA

CAGAGGTGTAAAGTACTTGAGTAATTTTACTTGATTACTGTACTTAAGTATTATTTTTGGGGATTTTTACTTTA
CTTGAGTACAATTAAAAATCAATACTTTTACTTTTACTTAATTACATTTTTTTAGAAAAAAAAGTACTTTTTAC
TCCTTACAATTTTATTTACAGTCAAAAAGTACTTATTTTTTGGAGATCACTTCATTCTATTTTCCCTTGCTATT
ACCAAACCAATTGAATTGCGCTGATGCCCAGTTTAATTTGTAGGGATAACAGGGTAATAACTTCGTATAATGTA
TGCTATACGAAGTTATCAGCATGCTTAGCTAGTGTAAATGAGAAAAAAAAGACCGGTAACTGAGCCTAATCATT
TAATCTAGCTTTATTTAAACATTCTAATTAAACGTTCGAATTGATATATGCGAGCTGAAAATGTAAAAAGCTTA
AAGATCAACTCACCTTATGTTTGGGGGGGTTTGTTCATCGATAAGCTTGATATCGAATTCCTGCCGGTCTGGAC
ATTTATTTGTATAGTTCATCCATGGCCATGTGTAATCCCAGCCAGCTGA
**GBT vector Sequence**

**Topo TA vector**
AAGGGCGAATTCGTTTAAACCTGCAGGACTAGTTCCTTTAGTGGAGAGGTTAATTCTGAGCTTGGCGTAATCAT
GGTCATAGCTGTTTCCTGTGTGAAATTGTTATCCCGCTCACAATTCACACAACCATACGAGCCGGAAAGCA
TAAAGTGTAAAGCCTGGGGGTGCCTAAAGAGTTGAACTTACCTCACATTTAATTGCGTTGCGTCCACGGCCCGC
CTTTCCAGTGAGGAAACCTTGTTCTGGCCGCCTGCATTAAATTGAACTCGCCAACGCCGCGGGGAGAGGCGGTT
TTGTCTATATGGG

## **Reverse seq /Ap03/AP_676_ssb**

GCCCAACCAGGAATTACCCCTCACTAAAGGGACTAGTCCTGCAGGTTTAAACGAATTCGCCCTT—**Topo TA vector**

CAGCTGCTGGGATTAC
ACATGGCATGGATGAACTATACAAATAAATGTCCAGACCGGCAGGAATTCGATATCAAGCTTATCGATGAACAA
ACCCCCCCAAACCTAAGGTGAGTTGATCTTTAAGCTTTTTACATTTTCAGCTCGCATATATCAATTCGAACGTT
TAATTAGAATGTTTAAATAAAGCTAGATTAAATGATTAGGCTCAGTTACCGGTCTTTTTTTTCTCATTTACACT
AGCTAAGCATGCTGATAACTTCGTATAGCATACATTATACGAAGTTATTACCCTGTTATCCCTACAAATTAAAC
TGGGCATCAGCGCAATTCAATTGGTTTGGTAATAGCAAGGGAAAATAGAATGAAGTGATCTCCAAAAAATAAGT
ACTTTTTGACTGTAAATAAAATTGTAAGGAGTAAAAAGTACTTTTTTTTCTAAAAAAATGTAATTAAGTAAAAG
TAAAAGTATTGATTTTTAATTGTACTCAAGTAAAGTAAAAATCCCCAAAAATAATACTTAAGTACAGTAATCAA
GTAAAATTACTCAAGTACTTTACACCTCTG—**GBT vector sequence**
**Insert-**
TATAGGTCAGTAGTCTTGAAACGCAGAGAGTTCACTACAAATGGACTTCTTTGAGTCTGTTGAAGAATGGTTCC
AGAAAGCAGCTTTAAGAAAATCTAAGCCAAAAAAAAAAAAAAAAAAAGTACTAGTCGACGCGTGGCCC

AAGGGCGAATTCGCGGCCGCTAAATTCAAGTTCGCCCTATAAGTGAGTCGTATTACAATTCACTGGCCGGTCGT
TTTAACGACGTCGTGACTGGGAAAACCCTGGCGTTTACCCAAACTTAATCGCCTTGCAGCACATTCCCCCTTTC
CGCCAGCTGGCGGTAATAGCGAAGAGGCCCGCCACCGATCGCCCTTTCCACAGGTTGGCGCAGCCTATAACGGT
ACGGCAGGTTGAAGGTTTACACCTTATCAAGAAGAGAGAGCCGTATTTCGTTTTGTTTGTGGATGTACAAGAGT
GATATTTATTGGACACGGCGGGGCGAACGAATGTGAATCCCCTTGGCCCGAGGCACGTCTCGTCTGTC –
**Topo TA vector sequence**

### (C) 5’ RACE PCR Product

5’-
ATATTATTATATAGCCATTTTGAATTTTATGAAAAGAAATTAAGGAAACTTTTATGCTTTGTAGATTCACATTCAATCCAGT
GCTTTGTAATTATTTGTGAAATCTACTCACAACTGCAACACCGGTTCATTTGGAAAACGTAGCCCCATATACATTTCTGGAG
ATCACAAATTAAGTAGCCAGAAGTACGTACACTGTATGGCTGCATTTGTCTTTAAAATGAACGGGCGGTATGATGATGTTCC
TTATGTGCCTTTACTCACTTACTGCTTACCTCTGTACGGCTCCGTAAAGCTCTGTACGGCTTTTCCGCTGTTAT
